# A genomic catalog of the mouse gut virome reveals features associated with ageing

**DOI:** 10.1038/s41467-026-75836-6

**Published:** 2026-07-21

**Authors:** Han-June Kim, Nayeon Kim, Jun Hyung Cha, Wonjong Kim, Junyeong Ma, Jungyeon Kim, Yerin Kim, Sunmo Yang, Sanguine Byun, Eunjung Lee, Martin Hemberg, Insuk Lee

**Affiliations:** 1 Department of Biotechnology, College of Life Science and Biotechnology, Yonsei University, Seoul, Republic of Korea; 2 DECODE BIOME Co. Ltd, Incheon, Republic of Korea; 3 Fermentation Convergence Research Group, Korea Food Research Institute, Wanju, Republic of Korea; 4 Gene Lay Institute of Immunology and Inflammation, Brigham and Women’s Hospital and Harvard Medical School, Boston, MA USA

**Keywords:** Metagenomics, Genome informatics, Microbiome

## Abstract

The gut virome is emerging as a key contributor to host physiology, yet it remains underexplored relative to the gut bacteriome. Despite comprehensive viral catalogs for the human gut, comparable resources for mice, the principal model organism in biomedical research, remain lacking, limiting our understanding of virome-host interactions and their implications for host health and disease. Here, we present Mouse Reference Gut Virome (MRGV), a comprehensive catalog comprising 109,778 viral genomes with ≥50% completeness and representing 28,824 species, expanding known mouse gut viral diversity by ~67.6%. Viral genome binning accounted for ~36% of cataloged genomes and improved average completeness by ~60% relative to contig-level assemblies. Structure-based annotation assigned functions to nearly half of 8.2 million viral proteins. Comparison with human gut virome revealed pronounced taxonomic and functional divergence. MRGV enabled host linkage for 88% of viral genomes and validated diverse mouse-associated crAss-like lineages with lineage-specific host-range and lifestyle strategies. Finally, mouse gut virome features harbored host ageing-associated signals distinct from those captured by bacterial features, with most ageing markers derived from binned genomes, highlighting the importance of viral binning for elucidating virome-host interactions.

## Abstract (web summary)

Here, the authors present a comprehensive mouse gut virome catalog and show that it features host ageing-associated signals distinct from those of the bacteriome, with most ageing markers recovered through viral genome binning.

## Introduction

The gut microbiome is a critical modulator of host health, yet decades of research have focused predominantly on bacterial communities^1,2^. This bacterial-centric view overlooks bacteriophages, whose abundance in the human gut varies substantially from approximately 1:100 to 4:1 relative to bacteria, reflecting the balance between lytic and lysogenic lifestyles^3^. These viruses shape microbial community structure through predation, lysogeny, and horizontal gene transfer^4^, and they hold substantial therapeutic potential for addressing antibiotic resistance, inflammatory bowel disease, and metabolic disorders^5^.

Laboratory mice are central to translational microbiome research, enabling controlled investigation of host-microbiota interactions^6^. While comprehensive genomic catalogs of human and mouse gut bacteriomes have enabled systematic cross-species comparisons^7,8^, equivalent resources for the mouse gut virome remain limited. Although the Unified Human Gut Virome (UHGV)^9^ has cataloged extensive human gut viral diversity, existing mouse-specific resources such as the high-confidence Mouse Gut Virome (hcMGV)^10^ capture only a fraction of the viral diversity in mice, constraining translational studies^11^.

Here, we present Mouse Reference Gut Virome (MRGV), the most comprehensive genomic catalog of the mouse gut virome to date. MRGV comprises 109,778 viral metagenome-assembled genomes (vMAGs) with ≥50% completeness, including 3810 complete genomes and 32,618 high-quality genomes (>90% completeness), representing 28,824 species. By incorporating viral genome binning, MRGV rescued over 280,000 contigs that would otherwise have been discarded; binned genomes account for ~36% of the catalog and increase average genome completeness by ~60%. Consequently, MRGV expands known mouse gut viral diversity by ~67.6% at the species level compared to hcMGV. Functional annotation of 8.2 million viral protein-coding sequences using structure-based homology assigned functions to nearly half. Comparison between MRGV and UHGV revealed pronounced taxonomic and functional divergence between the mouse and human gut virome.

MRGV further enabled systematic characterization of virus-host interactions and viral lifestyles in the mouse gut. Integrating CRISPR spacer matches and prophage signals linked 88% of viral genomes to bacterial hosts, uncovering lineage-specific infection strategies and broad host ranges that often transcend species boundaries. Using phylogenetic and genomic criteria, we confirmed diverse mouse-associated crAss-like lineages and revealed lineage-specific host-range and lifestyle strategies shaped by their functional genomic architectures.

Finally, leveraging MRGV, we demonstrate that gut virome features encode host ageing-associated signals distinct from those of bacterial features in mice. Most viral ageing markers were derived from binned genomes, highlighting the critical importance of viral binning for constructing informative virome references and for elucidating viral contributions to host health and disease.

## Results

### Construction and overview of the MRGV

**Fig. 1:**
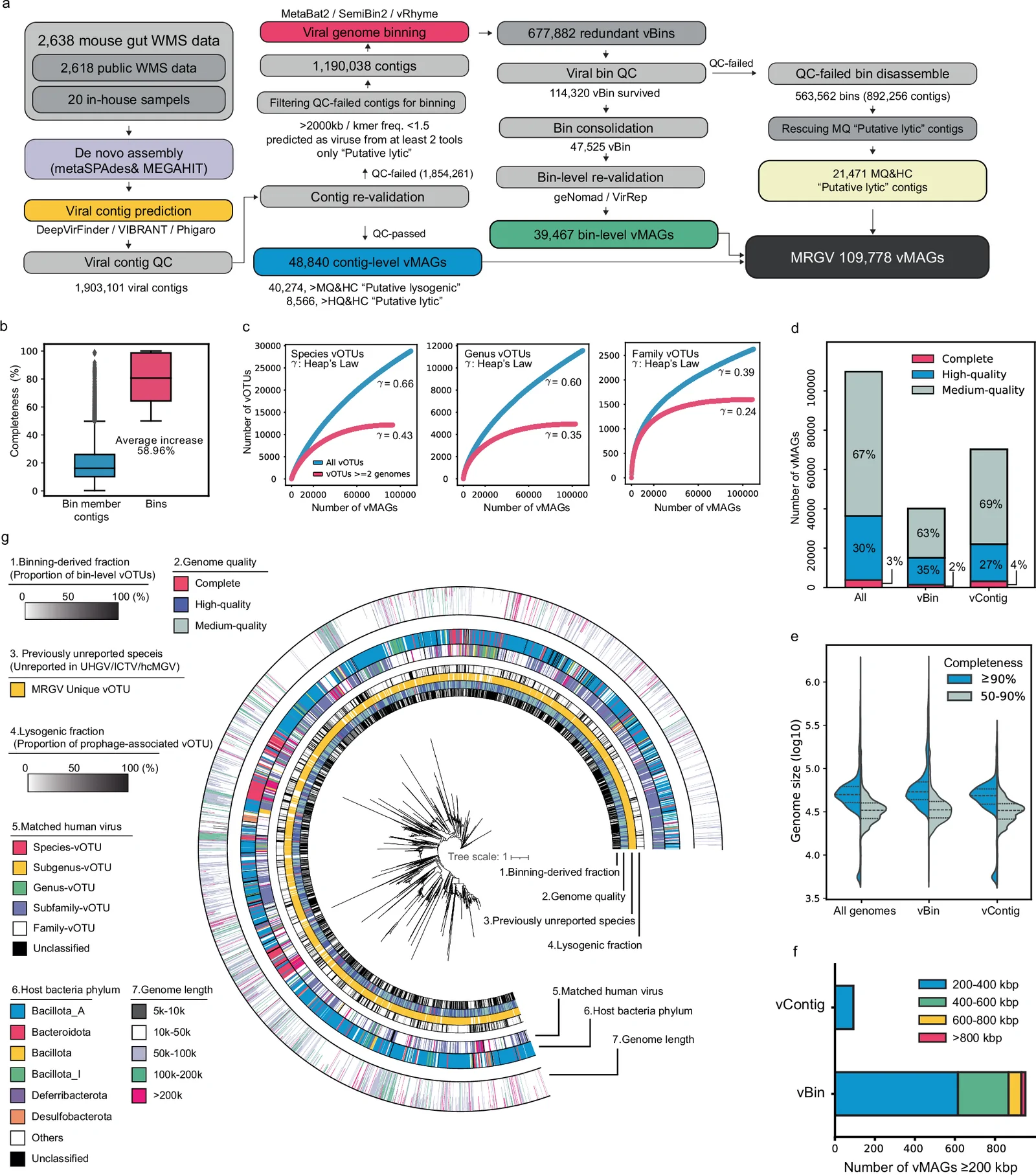
Overview of mouse reference gut virome (MRGV). **a** Summary of the MRGV construction workflow. **b** Completeness of 39,467 bin-level genomes and their member contigs. For boxplots, box lengths represent the interquartile range of the data (IQR), and whiskers extend to 1.5x IQR. The center bar represents the median. **c** Rarefaction analysis at 28,824 species-level, 11,578 genus-level, and 2629 family-level vOTUs. Heaps’ law coefficient reflects the rate of new vOTU discovery, with lower values indicating saturation and higher values indicating continued accumulation of rare taxa. **d**. Genome quality distribution of 109,778 all viral genomes, 39,467 bin-level genomes (vBin), and 70,311 contig-level genomes (vContig). **e** The length distribution of high-quality (blue) and medium-quality (gray) genomes. **f** The length distribution of genomes with ≥ 200 kbp. **g** Phylogenetic tree of 22,679 out of 28,824 MRGV species-level vOTUs based on TerL sequences.

A total of 2638 mouse gut whole metagenome sequencing (WMS) datasets, comprising 20 newly generated samples and 2618 publicly available datasets (Supplementary Data 1), were processed using an in-house workflow to construct viral metagenome-assembled genomes (vMAGs) (Fig. 1a**;** Supplementary Fig. 1**; Methods**). This workflow was designed with conservative criteria and thresholds informed by previous large-scale virome studies^12–17^, prioritizing high confidence and minimizing false discovery.

High-quality sequencing reads were assembled into contigs using MetaSPAdes^18^ as the primary assembler and MEGAHIT^19^ as an alternative. Putative viral contigs were identified using three complementary predictors, DeepVirFinder^20^, VIBRANT^21^, and Phigaro^22^, followed by sample-wise deduplication and stringent decontamination to exclude bacterial DNA and mobile genetic elements. Candidate viral contigs were further revalidated using two additional neural network-based classifiers, geNomad^23^ and VirRep^24^, together with additional quality filters, yielding 48,840 high-confidence contig-level vMAG that consist of 8566 high-quality (completeness ≥ 90%) ‘lytic’, and 13,517 high-quality and 26,757 medium-quality (90% > completeness ≥ 50%) ‘lysogenic’ vMAGs in this initial contig curation step.

Because most viral contigs were fragmented and failed to meet contig-level vMAG criteria, they would otherwise have been discarded. To rescue these genomes, we performed viral binning on ~1.19 million high-confidence lytic viral contigs, while explicitly excluding contigs predicted as putative prophages to avoid algorithmic ambiguity and facilitate biological interpretation of the resulting bins. Three independent binning tools, MetaBat2^25^, SemiBin2^26^ and vRhyme^27^, were applied, followed by stringent bin-level quality assessment and sample-wise bin consolidation to eliminate redundancy. This process produced 39,467 high-confidence bin-level vMAGs, rescuing 287,782 contigs. Furthermore, viral genome binning increased average genome completeness by ~60% relative to member contigs, underscoring the value of incorporating bin-level vMAGs into viral genome catalogs (Fig.1b).

To further recover moderately complete viral genomes, bin-level assemblies that failed to meet vMAG criteria (563,562 bins comprising 892,256 contigs) were disassembled into individual contigs, and contig-level vMAG criteria were reapplied. This rescue step yielded an additional 21,471 medium-quality contig-level lytic vMAGs (90% > completeness ≥ 50%).

Collectively, these efforts resulted in the MRGV, a comprehensive genomic catalog of the mouse gut virome comprising 109,778 high-confidence vMAGs. This catalog includes 70,311 contig-level vMAGs and 39,467 bin-level vMAGs (Supplementary Data 2, 3).

Species-level viral operational taxonomic units (vOTUs) were defined by clustering vMAGs at 95% average nucleotide identity (ANI) and 85% alignment fraction (AF), resulting in 28,824 species-level vOTUs. Higher-rank vOTUs at the genus and family levels were delineated using average amino acid identity (AAI) and gene-sharing thresholds calibrated against reference genomes from the International Committee on Taxonomy of Viruses (ICTV)^28^, yielding 11,578 genus-level vOTUs (vGENs) and 2629 family-level vOTUs (vFAMs) (Supplementary Data 4). Rarefaction analysis indicated that family-level vOTU accumulation curves approached saturation (Heaps’ Law coefficient = 0.39), whereas species- and genus-level curves remained unsaturated (Heaps’ Law coefficient = 0.66 and 0.60, respectively) (Fig. 1c**;** Supplementary Data 5), reflecting a pronounced long tail of rare taxa, including a substantial fraction of singletons (16,663 of 28,824 vOTUs; 57% and 6636 of 11,578 vGENs; 57%).

All 109,778 vMAGs in MRGV met at least medium-quality criteria. Approximately 30% (32,618) were classified as high-quality, and ~3% (3810) represented complete genomes (100% completeness and terminal repeat sequences are detected) (Fig. 1d). Overall genome-quality profiles were comparable between contig-level and bin-level vMAGs, although bin-level vMAGs contained a slightly higher proportion of high-quality genomes. Genome-size distributions were broadly similar between the two groups; however, viral binning disproportionately enabled recovery of large genomes (Fig. 1e). Notably, the vast majority of genomes ≥200 kbp were reconstructed through bin-level vMAGs (952 of 1044; 91.2%; Fig. 1f), indicating that binning is particularly effective for recovering jumbo and other large-genome phages that are difficult to assemble as single contigs.

We identified many previously unreported mouse gut viral species in MRGV. Approximately 67.6% (19,473 of 28,824) species-level vOTUs did not cluster with any species from the former mouse virome catalog^10^ (Fig. 1g), indicating substantial unexplored viral diversity in the mouse gut. In addition, only ~22% (24,077 of 109,778) of the viral genomes could be assigned to an existing viral taxonomy at least at the family rank (Supplementary Data 6). The lysogenic lifestyle of viral genomes was inferred based on multiple lines of lysogeny-associated evidence. Using the criteria described in the **Methods**, 71,978 viral genomes were classified as lysogenic, while the remaining 37,800 genomes were classified as lytic (Supplementary Data 7).

### Mouse-human gut viromes show species divergence but higher-rank compositional conservation

**Fig. 2:**
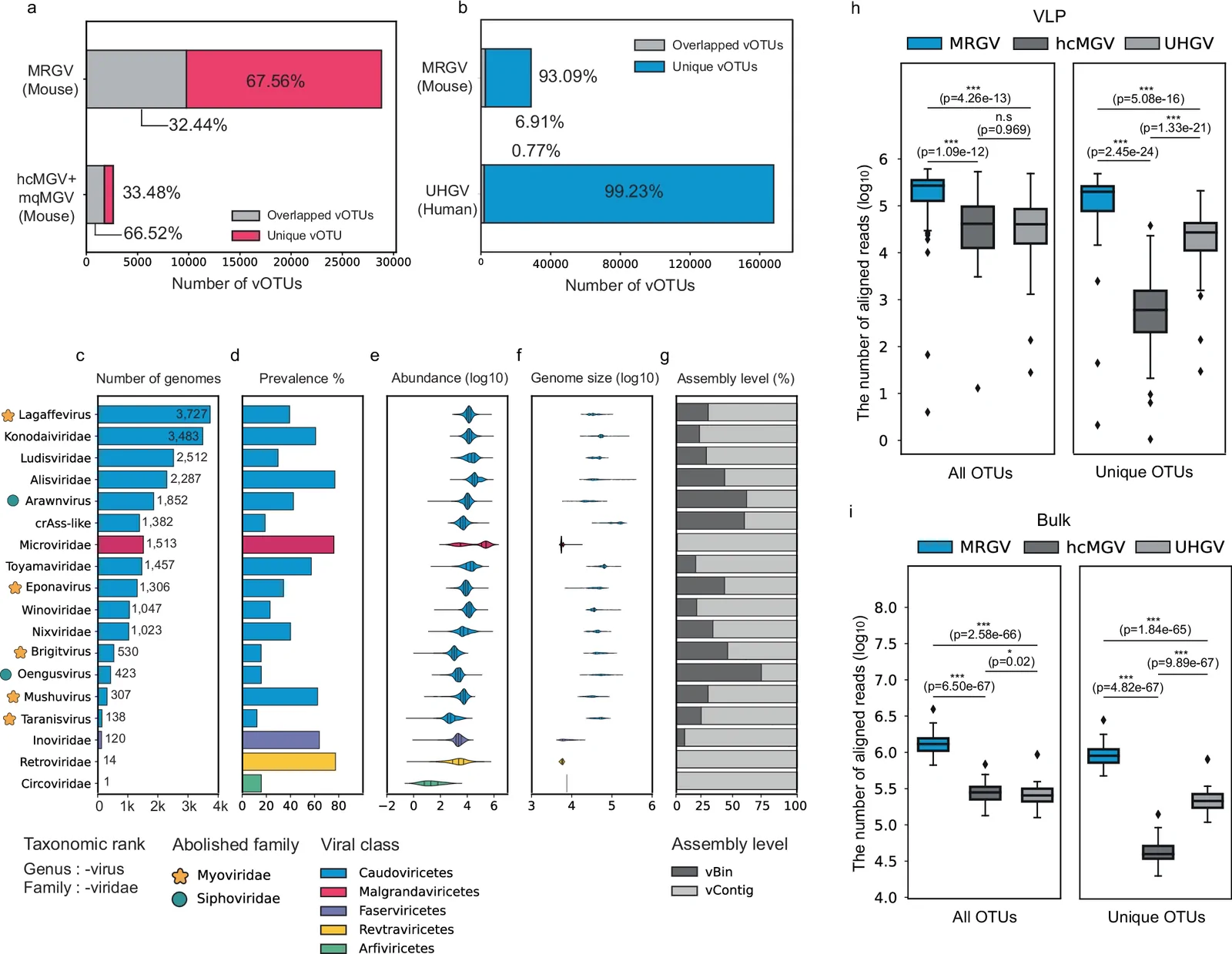
Taxonomic analysis of MRGV genomes and their comparison with other catalogs. **a** Overlap analysis of species-level vOTUs between MRGV and hcMGV (95% ANI and 85% AF). **b** Overlap analysis of species-level vOTUs between MRGV and UHGV (95% ANI and 85% AF). **c**–**g** Taxonomic landscape of MRGV at family- and genus-level. ICTV taxonomically annotated viral genus and families. Viral genus marked with a yellow star or a turquoise circle indicates ICTV legacy taxonomy. The number of recovered genomes grouped by family or genus rank (**c**). Prevalence was measured by counting viral genomes with >50% alignment coverage calculated using CoverM (**d**). The abundance of each viral family or genera at log10 scale (**e**). The distribution of genome size of each viral family or genera at log10 scale (**f**). Proportion of bin-derived genomes (vBin) belonging to each viral family or genera (**g**). **h**, **i **The number of reads aligned to each catalog, measured using 79 VLP-enriched (**h**) and 50 whole metagenomic sequencing datasets (**i**) from mouse fecal samples with Minimap2. For boxplots, box lengths represent the interquartile range of the data, and whiskers extend to the lowest and highest values within 1.5 times the interquartile range from the first and third quartiles, respectively. The center bar represents the median. Statistical significance was assessed using a two-tailed Mann-Whitney U test (n.s. non-significant, * *P* < 0.05, *** *P* < 0.001).

A recent mouse gut virome resource^10^, reported 977 high-confidence (hcMGV) and 1645 medium-quality (mqMGV) species-level vOTUs derived from 407 mouse WMS datasets. We quantified overlap between hcMGV+mqMGV and MRGV at the species level using the standard demarcation criteria (95% ANI, 85% AF). Although 67.56% (19,473 of 28,824) of MRGV species-level vOTUs were not represented in hcMGV+mqMGV, 66.52% (1744 of 2622) of hcMGV+mqMGV species-level vOTUs were recovered by MRGV (Fig. 2a), reflecting the broader phylogenetic coverage of MRGV.

Cross-host comparison revealed limited overlap between mouse and human gut virome: only 1989 of 28,824 MRGV species-level vOTUs (6.91%) matched 0.77% of the 168,536 species-level vOTUs in UHGV (Fig. 2b). This minimal overlap parallels the previously reported low overlap of gut bacterial species between mice and humans^7^ and supports the notion that gut phage communities closely track host-associated bacterial communities. Together, these results suggest distinct host- and habitat-specific diversification of gut virome driven by phage-host coevolutionary dynamics operating at shallow evolutionary timescales^7,29^.

Consistent with broad intestinal virome patterns, the mouse gut virome was dominated by bacteriophages, particularly double-stranded DNA (dsDNA) tailed phages within Caudoviricetes^13^. Among the most prevalent lineages, several correspond to phage groups that are also prominent in human gut studies under the updated ICTV taxonomy, including genera reassigned from legacy Myoviridae and Siphoviridae, indicating partial conservation of higher-rank taxonomic structure across hosts^30^ (Fig. 2c). In addition to these dominant tailed phage groups, families originally described from bacteriophages infecting the oral pathogen *Porphyromonas gingivalis*, including Ludisviridae, Alisviridae, and Nixviridae^31^, were also prevalent and abundant in the mouse gut. This observation suggests that lineages first recognized in oral-associated niches may be more broadly distributed across mammalian gut microbiomes than previously appreciated (Fig. 2d, e). CrAss-like phages are widely recognized as major components of the human gut virome, in some cases accounting for up to 90% of total viral loads^32,33^. In the mouse gut, we recovered 1382 crAss-like viral genomes with relatively large genome sizes (mean genome size in MRGV: 132 kbp; Fig. 2f). These viruses constituted a highly abundant component of the mouse gut virome and were detected in ~20% of mice, consistent with prevalence estimates reported in a previous human metagenomic study^15^. Beyond Caudoviricetes, single-stranded DNA (ssDNA) Microviridae constituted a common and prevalent component of the mouse gut virome, filamentous Inoviridae were also frequently detected, and Retroviridae-like genomes were recovered across most mice^34,35^ (Fig. 2d-e).

Genome recovery exhibited a clear dependence on genome size. Small-genome families were largely captured as single contigs, with 98% of Microviridae, Inoviridae, Retroviridae-like, and Circoviridae genomes assembled at the contig level, whereas larger-genome groups were preferentially reconstructed through binning, with 61% of Arawnvirus, crAss-like, and Oengusvirus genomes derived from bin-level assemblies **(**Fig. 2g**)**. These results indicate that viral binning is essential to avoid systematic loss of large and fragmented genomes and to recover both the conserved higher-rank taxonomy structure and the mouse-specific components of the gut virome. Collectively, these findings establish MRGV as a comprehensive, mouse-specific reference virome that preserves the shared higher-rank architecture of intestinal phage communities while capturing marked species-level divergence from humans. Moreover, they underscore that incorporating viral binning is critical for the comprehensive reconstruction of large and fragmented viral genomes that would otherwise be systematically missed.

### MRGV enhances taxonomic profiling of the mouse gut virome

A major goal of constructing a comprehensive reference viral genomic catalog is to improve taxonomic profiling of the corresponding virome. Given the expansion of species-level vOTU diversity in MRGV, we anticipated increased alignment of metagenomic sequencing reads to MRGV compared with existing gut viral genome catalogs, including hcMGV and UHGV. To evaluate taxonomic profiling performance, we assessed read alignment against each catalog using both WMS data from 50 mouse fecal samples^36^ and viral-like particle (VLP)-enriched sequencing data from 79 mouse gut samples^37^ (Supplementary Data 8).

Across both data types, MRGV consistently achieved higher alignment rates than hcMGV and UHGV, with particularly significant gains for MRGV-unique vOTUs (Fig. 2h–i). Notably, the human gut viral genome catalog UHGV showed markedly lower alignment rates in both datasets. Given that UHGV is substantially larger than hcMGV and encompasses broad human gut viral diversity, its poor performance on mouse metagenomic data further supports the conclusion that mouse gut viral species are largely distinct from those in humans. Collectively, these results demonstrate that MRGV enhances taxonomic profiling capacity for mouse gut virome analyses across metagenomic sequencing modalities.

### MRGV reveals host-specific functional divergence of gut virome

**Fig. 3:**
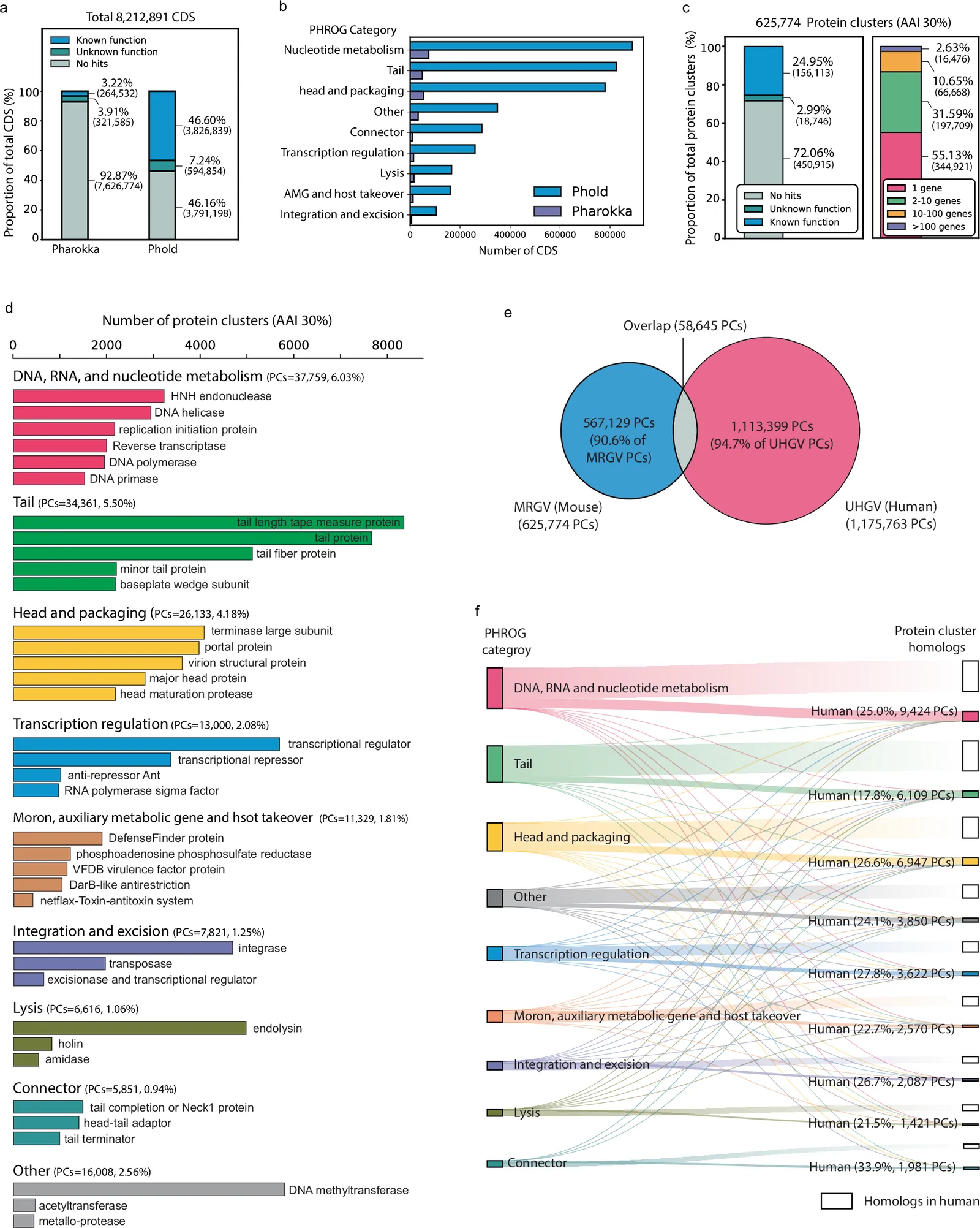
Protein functional landscape of MRGV and divergence between mouse and human gut virome. **a** Functional annotation of CDS from MRGV genomes using sequence-based (Pharokka) and structure-based (Phold) approaches. **b** Distribution of annotated CDSs across PHROG functional categories based on sequence (Pharokka) and structure (Phold) alignment, highlighting improved recovery of core viral functions by structure-based annotation. **c** Composition of MRGV protein clusters (PCs) with 30% AAI, showing proportions of clusters with known/unknown/no-hit annotations (left) and cluster size distribution (right). **d** Most abundant PCs within major PHROG functional categories. **e** Overlap of MRGV and UHGV PCs, demonstrating limited sharing of viral protein families between mouse and human. **f** Sankey diagram showing category-specific overlap of PCs between MRGV and UHGV. Percentages indicate the fraction of MRGV PCs with detectable homologs in UHGV for each PHROG category, revealing pervasive functional divergence across both core and accessory viral modules.

We predicted 8,212,891 protein-coding sequences (CDSs) from 109,778 MRGV genomes. Of these, only 586,117 CDSs (7.14%) showed detectable sequence homology to reference databases, and 264,532 (3.22%) could be functionally annotated by sequence-based methods (**Methods;** Fig. 3a). Because protein structure is more conserved than primary sequence over long evolutionary distances^38^, we further applied structure-informed annotation using Phold^39^ with 3Di structural representation alignment, which assigned PHROG^40^ functions to 3,826,839 CDSs (46.60%), substantially expanding functional coverage across diverse categories (Fig. 3b).

Clustering proteins at 30% amino-acid identity (AAI) yielded 625,774 protein clusters (PCs), of which 156,113 (24.95%) were assigned to known PHROG functions (**Methods;** Fig. 3c). The cluster size distribution was highly skewed: 55.13% were singletons (344,921 PCs), 31.59% contained 2–10 proteins (197,709 PCs), and only 2.63% exceeded 100 members (16,476 PCs). Among annotated PCs, core viral functions dominated, with DNA/RNA/nucleotide metabolism (37,757 PCs, 6.03%), tail proteins (34,361, 5.50%), and head and packaging (26,133, 4.18%) being the most prevalent (Fig. 3d). The largest PCs comprised canonical phage modules, including HNH endonucleases, helicases, replication initiators, tail tape-measure and tail proteins, portal proteins, and major capsid proteins.

Importantly, MRGV also captures diverse non-core functional diversity central to virus–host ecology in mice. A marked representation was observed for transcription regulation (13,000 PCs, 2.08%), integration and excision (7821, 1.25%), and lysis (6616, 1.06%), as well as diverse host-takeover and auxiliary functions, including toxin–antitoxin systems, anti-defense proteins, and auxiliary metabolic enzymes. These functions are typically lineage-specific and poorly recoverable by sequence-only annotation; their inclusion in MRGV markedly enhances the interpretability of mouse virome datasets for studying temperate-lytic strategies, host defense evasion, and phage-mediated modulation of bacterial physiology.

We next quantified functional overlap between mouse and human gut virome at the PC level. Only 9.4% of MRGV PCs (58,645 of 625,774) showed detectable homology to UHGV PCs (**Methods;** Fig. 3e). Stratification by PHROG category revealed pervasive divergence across both core and non-core modules, with overlaps ranging from 17.8% to 33.9% (Fig. 3f). Core replication and morphogenesis functions showed modest sharing, for example, nucleotide metabolism (25.0%; 9424 PCs) and head and packaging (26.6%; 6947 PCs), while tail proteins exhibited the lowest overlap (17.8%; 6109 PCs), consistent with rapid diversification of host-range determinants shaped by phage–host arms races^41,42^. In contrast, connector proteins showed the highest overlap (33.9%; 1981 PCs), reflecting more conserved structural constraints on head–tail junction and tail-completion components essential for virion assembly and genome delivery^43,44^.

Collectively, the limited protein-family sharing indicates that the mouse and human gut virome are functionally divergent at the protein-family level despite broadly conserved phage architecture. These findings underscore the necessity of a mouse-specific reference framework for rigorous functional profiling and ecological interpretation of mouse gut virome, as human-derived catalogs do not adequately represent the underlying protein-family diversity.

### Host-resolved structure and broad host range of the mouse gut virome

To infer bacterial hosts for uncultivated mouse gut viruses, we integrated two orthogonal linkage signals reflecting distinct biological processes: CRISPR spacer matches, which record adaptive immune memory of prior viral exposure, and near-identical virus-bacterium alignments, which indicate integrated prophages (**Methods**). We prioritized these alignment signals to rigorously confirm persistent infections, addressing the limitations of conventional tools that frequently miss integrated prophages and are predicted to be virulent^45^. Combining concordant evidence from both signals increases confidence in host assignment^46^. For the analysis, we utilized bacterial genomes from the mouse reference gut microbiome (MRGM)^7^, the most comprehensive catalog of mouse gut prokaryotic genomes. Using this framework, 96,850 of 109,778 viral genomes (88.2%), corresponding to 22,758 of 28,824 species-level vOTUs (78.9%), were linked to at least one bacterial host (Supplementary Data 9). Among these, 44,791 genomes were supported by both CRISPR and integration evidence, 31,581 by CRISPR alone, and 20,478 by integration alone.

**Fig. 4:**
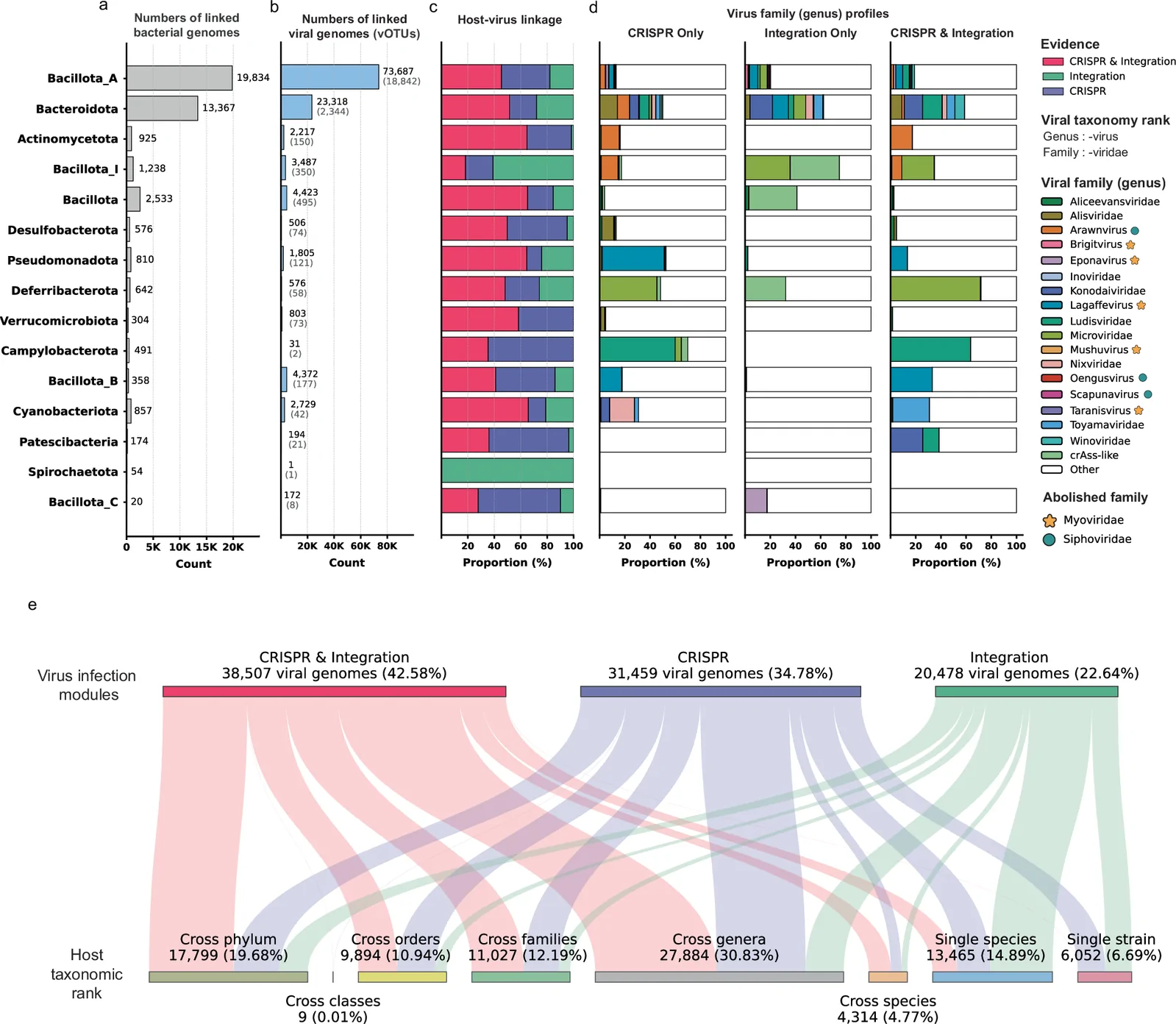
Host linkage and host breadth of mouse gut virome. **a** Number of linked host bacterial genomes of MRGM per phylum. **b** Numbers of linked viral genomes and corresponding vOTUs in parentheses per phylum. **c** Composition of host-virus linkage evidence by host phylum. **d** Family/genus-level profiles of the linked viruses per phylum across linkage evidence types. Top 20 viral genus- or family-level taxonomies with the largest host-virus connection were shown. ICTV taxonomically unclassified and other than the Top 20 viral taxa were treated as ‘Other’ groups. **e** Sankey summary of inferred host breadth for host-linked viral genomes, showing the distribution of links across host taxonomic ranks (single strain/species vs cross-taxon links) across evidence types.

Host-linked viruses predominantly tracked the two major bacterial lineages of the mouse gut (Fig. 4a–b). Bacillota_A accounted for the largest number of linked bacterial genomes (19,834 of 42,245 MRGM genomes) and the greatest number of linked viruses (73,687 MRGV genomes spanning 18,842 vOTUs), followed by Bacteroidota (13,367 MRGM genomes and 23,318 MRGV genomes across 2344 vOTUs). This phylum-level skew is biologically plausible, as these phyla comprise the majority of bacterial species in the mouse gut.

The relative contribution of CRISPR and integration evidence differed markedly among host phyla (Fig. 4c), consistent with uneven CRISPR–Cas distribution and differences between temperate and lytic viral populations^47,48^. Actinomycetota showed high concordance, with 64.8% of associations supported by both signals, consistent with frequent coexistence of CRISPR-Cas systems and prophages in gut-associated Actinomycetota such as Bifidobacterium^49^. In contrast, Bacillota_I exhibited an integration-skewed profile (60.8% integration-only), indicative of prevalent temperate phage lifestyles^48^. Bacillota_B was notable for a high density of host-linked viruses despite limited host representation: 358 bacterial genomes were linked to 4372 viral genomes (~12.2 viruses per host bacterial genome), with balanced support from CRISPR and integration signals. This pattern is consistent with compact host groups experiencing frequent phage exposure and carrying substantial temperate phage burdens^47,48^.

Analysis at the family and genus levels further showed that host-linked viruses are unevenly distributed across viral lineages and that linkage mode is informative of viral group identity (Fig. 4d). Integration-only associations revealed distinct lineage structure; for example, in Bacillota_I they were enriched for crAss-like and Microviridae-related viruses^50^. Although crAss-like phages are often described as lytic, integrated signals in Bacillota and Deferribacterota are consistent with reports of crAss-like homologs in bacterial genomes, potentially reflecting prophage-like insertions or recent gene exchange^51,52^.

Across the 96,850 host-linked viral genomes, we filtered 90,444 host-linkage genomes based on evidence support filtering **(Methods)**. The inferred host range of 90,444 was frequently broader than a single taxon (Fig. 4e). Only 6052 genomes (6.69%) were linked to a single strain and 13,456 (14.89%) to a single species, whereas many spanned multiple genera (30.83%), families (12.19%), orders (10.94%), or even phyla (19.68%). This apparent broad host range is consistent with recent observations from long-read metagenomics and proximity-ligation (Hi-C) studies^53,54^.

Collectively, these results establish MRGV as a host-resolved reference for the mouse gut virome, enabling more reliable virus-host assignment, comparative analysis of host-range structure, and prioritization of ecologically important virus-host systems for downstream validation and mechanistic studies.

### Phylogenetic validation and tRNA-driven stratification of mouse gut crAss-like phages

Crassvirales (crAss-like phages) are among the most prevalent and abundant dsDNA bacteriophages in the human gut^51,55^, and large cohort studies have linked their abundance to multiple host phenotypes, including depletion in inflammatory bowel disease (IBD)^56^. Recent large-scale cataloging efforts, particularly UHGV^9^, have substantially expanded the known diversity of human-associated Crassvirales, reporting over 5200 vOTUs. In contrast, the diversity and ecological landscape of crAss-like phages in other mammalian guts, including mice, remain comparatively underexplored. Although hcMGV^10^ recently recovered Crassvirales genomes from multiple hosts (6, 276, and 33 vOTUs from mouse, pig, and cynomolgus macaque, respectively), its limited cohort size constrained comprehensive characterization.

**Fig. 5:**
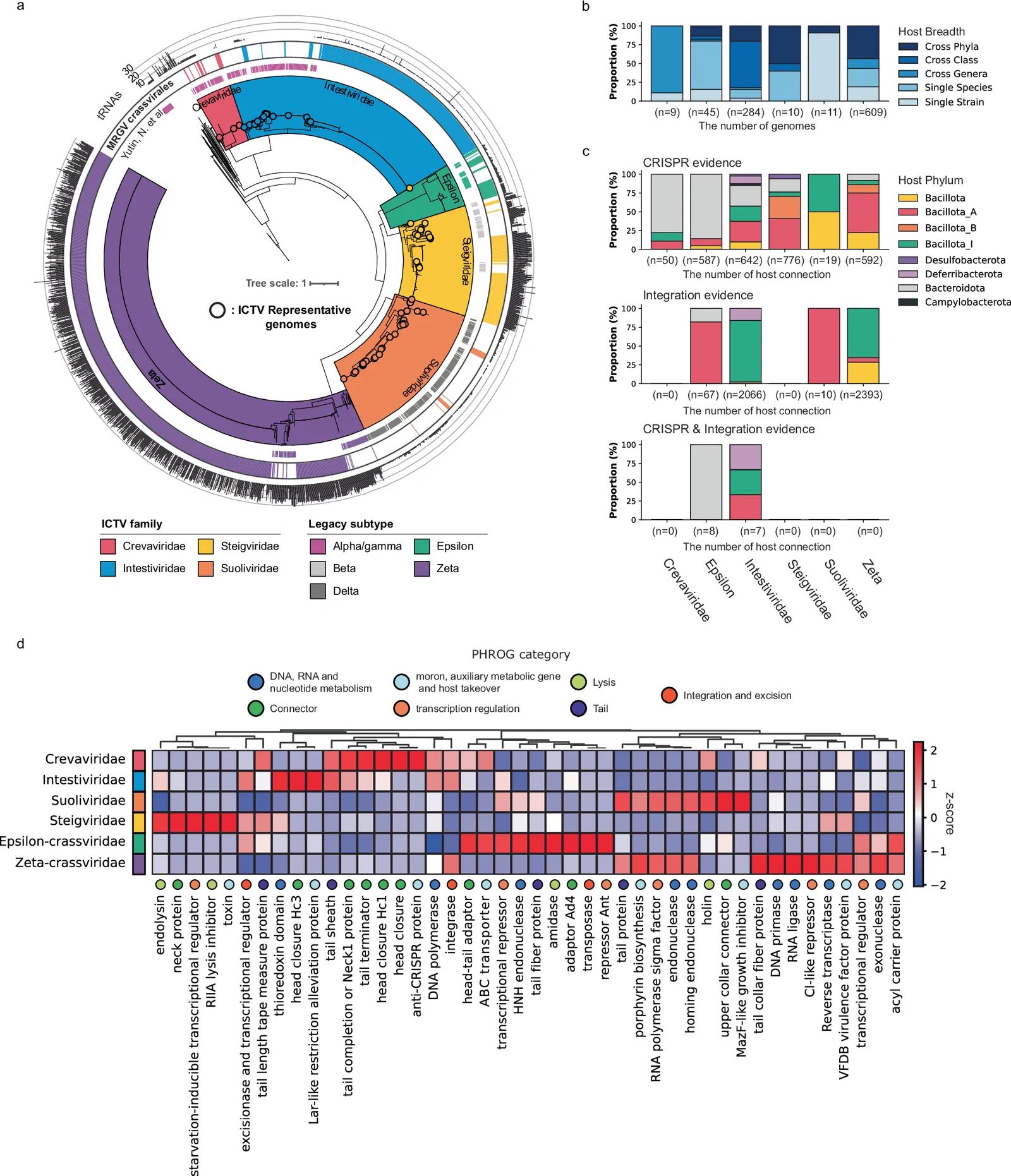
Analysis of crAss-like viruses in the mouse gut. **a** TerL marker gene-based phylogenetic analysis of 1382, 631, and 76 crAss-like virus genomes from MRGV, Yutin, N. et al., and ICTV VMR_MSL40.v1, respectively. ICTV families and legacy subtypes of crAss-like viruses are represented by color codes. Most outer rings show the number of tRNA genes in each viral genome, and the middle and inner rings show crAss-like viral genomes from MRGV and Yutin, N. et al., respectively. **b** Host breadth of crAss-like viruses. **c** Distribution of the number of subtype-resolved host connectivity profiles across host phyla for each evidence type. **d** Heatmap of the functional profiles for 1382 crAss-like viruses from MRGV. The genes were hierarchically clustered on the Euclidean metric by seaborn clustermap. The top 3 most abundant genes in each subtype belonging to 7 PHROG functional categories were selected.

To address this gap, we applied the *uhgv-classify* module to enable additional subtype-level annotation of Crassvirales, including the Epsilon and Zeta subtypes, and initially flagged 1569 MRGV genomes as putative crAss-like phages. To place these assignments within an ICTV-consistent framework, we reconstructed a terminase large subunit (TerL) phylogeny using the 1569 candidates together with 76 ICTV Crassvirales representatives and 631 curated crAss-like genomes from Yutin et al.^51^. Phylogenetic inference validated 1382 (215 vOTUs) of the 1569 candidates, spanning Crevaviridae (18 genomes, 11 vOTUs), Intestiviridae (333, 58), Steigviridae (142, 39), Suoliviridae (19, 5), and the Epsilon (49, 12) and Zeta (821, 90) subtypes (Fig. 5a).

This analysis, which leverages TerL as a standard phylogenetic marker for tailed dsDNA phages, resolved the four ICTV-recognized families as coherent family-level backbones while clarifying how legacy alpha–zeta subtype labels partially, but not fully, map onto official ICTV lineages. Such imperfect correspondence is consistent with the highly mosaic nature of crAss-like genomes^57^ and limitations of earlier heuristic subtype assignments^9^.

Notably, auxiliary translation genes were strongly lineage-stratified across Crassvirales. Viral-encoded tRNAs were enriched in specific clades, particularly Steigviridae (historical Beta subtype) and the Zeta subtype, whereas other lineages carried sparse or no tRNA complements (Fig. 5a). This pattern suggests divergent coding strategies, with tRNA-rich lineages exhibiting greater translational autonomy^58^ that may facilitate efficient protein synthesis across hosts with differing codon usage and GC content^59,60^.

### Lineage-specific host-range strategies of mouse gut crAss-like phages

Host-range inference across crAss-like lineages revealed pronounced family-level heterogeneity that broadly mirrored the tRNA-stratified TerL phylogeny (Fig. 5a, b). Suoliviridae exhibited the narrowest host range, with most genomes linked at the single-strain level (90.1%), whereas Crevaviridae predominantly showed multi-genus associations (88.9%). In contrast, Intestiviridae, Steigviridae, and the Zeta subtype displayed the broadest host ranges, with frequent cross-class to cross-phyla linkages (81.6%, 50.0%, and 43.7%, respectively; Fig. 5b). The Epsilon subtype was comparatively host-restricted, with most associations at the single-species or strain level, although a minority showed broader host breadth.

Linkage evidence further distinguished host-association strategies among lineages (Fig. 5c). Steigviridae showed broad CRISPR-supported host exposure despite limited genome recovery, consistent with dynamic tRNA repertoires and expanded host compatibility. Intestiviridae and Zeta subtype exhibited the largest number of host connections, supported by both CRISPR-only and integration-only evidence, with integration signals significantly enriched (Chi-square goodness-of-fit test, *P* < 7.28e−165 (*n* = 2066) and *P* < 2.65e−238 (*n* = 2393) respectively), suggesting flexible lifestyle strategies across host niches.

Host-phylum profiles differed by linkage evidence and lineage (Fig. 5c). CRISPR-supported associations for Crevaviridae and Epsilon subtype were concentrated in Bacteroidota, consistent with predominantly lytic lifestyles^59^. In contrast, Intestiviridae and Zeta subtype showed broad CRISPR-based host exposure across multiple phyla but preferential integration into Bacillota lineages, particularly Bacillota_I, indicating selective lysogeny in high-fitness hosts despite broad lytic interactions^61,62^.

Collectively, crAss-like phages exhibit lineage-specific host-range architectures. While Suoliviridae and Epsilon subtypes behave as host specialists, Intestiviridae, Steigviridae and Zeta subtypes function as ecological generalists, combining broad lytic host exposure with selective integration into specific bacterial niches. This distinction highlights a sophisticated survival strategy that reconciles broad ecological reach with lineage-specific stable maintenance.

### Functional gene repertoires differ across mouse gut Crassvirales lineages

Functional profiling revealed marked lineage-specific differences in accessory gene content **(**Fig. 5d**)**. Steigviridae showed enrichment of lytic- and host-impact-associated annotations, including endolysin, toxin, and transcriptional regulator genes, whereas integrase-related modules were rarely detected. This pattern contrasted with Intestiviridae, which displayed enrichment of integrase, excisionase, and repressor modules, together with anti-restriction, replication-support, and structural genes, consistent with a stronger representation of temperate-associated functions.

The Zeta subtype showed a similar enrichment of lysogeny-associated and regulatory modules, including CI-like repressors, integrase, transcriptional regulators, and an RNAP sigma factor. In addition, Zeta genomes were enriched for replication and nucleic-acid processing genes, including DNA primase, RNA ligase, endonuclease/exonuclease, homing endonuclease, and reverse transcriptase annotations, suggesting greater representation of genome-remodeling functions in this subtype. By contrast, the Epsilon subtype showed a host-specialist-associated profile, with enrichment of host-recognition modules, particularly tail fiber genes, together with amidase, transcriptional repressor, and nuclease annotations, but fewer genome-plasticity-associated functions.

Together, these results show that mouse gut Crassvirales lineages differ substantially in functional gene repertoires related to lysis, lysogeny, host recognition, replication, and genome remodeling. These differences provide genomic evidence consistent with lineage-specific variation in host breadth and predicted lifestyle across mouse gut crAss-like phages.

### The gut virome encodes ageing-associated signals

Age-related alterations of the gut microbiome have been proposed as targets for dietary-mediated lifespan extension^63^, and age-dependent changes in gut viral diversity have been reported using early human gut virome catalogs^12^. However, a quantitative model that estimates chronological age based on the gut virome has not yet been established, and it remains unclear whether virome dynamics can reliably capture ageing processes. Recently, age-associated gut bacteriome features were characterized in mice using longitudinal WMS data from 2997 fecal samples collected from 913 Diversity Outbred (DO) mice^36^ (Supplementary Data 10).To examine age-associated dynamics of the virome in mice, we profiled viral taxonomic abundances from the same cohort using the MRGV custom database with Kraken2/Bracken software^64,65^, applying a stringent ≥10% genome-coverage filter to minimize false positives.

**Fig. 6:**
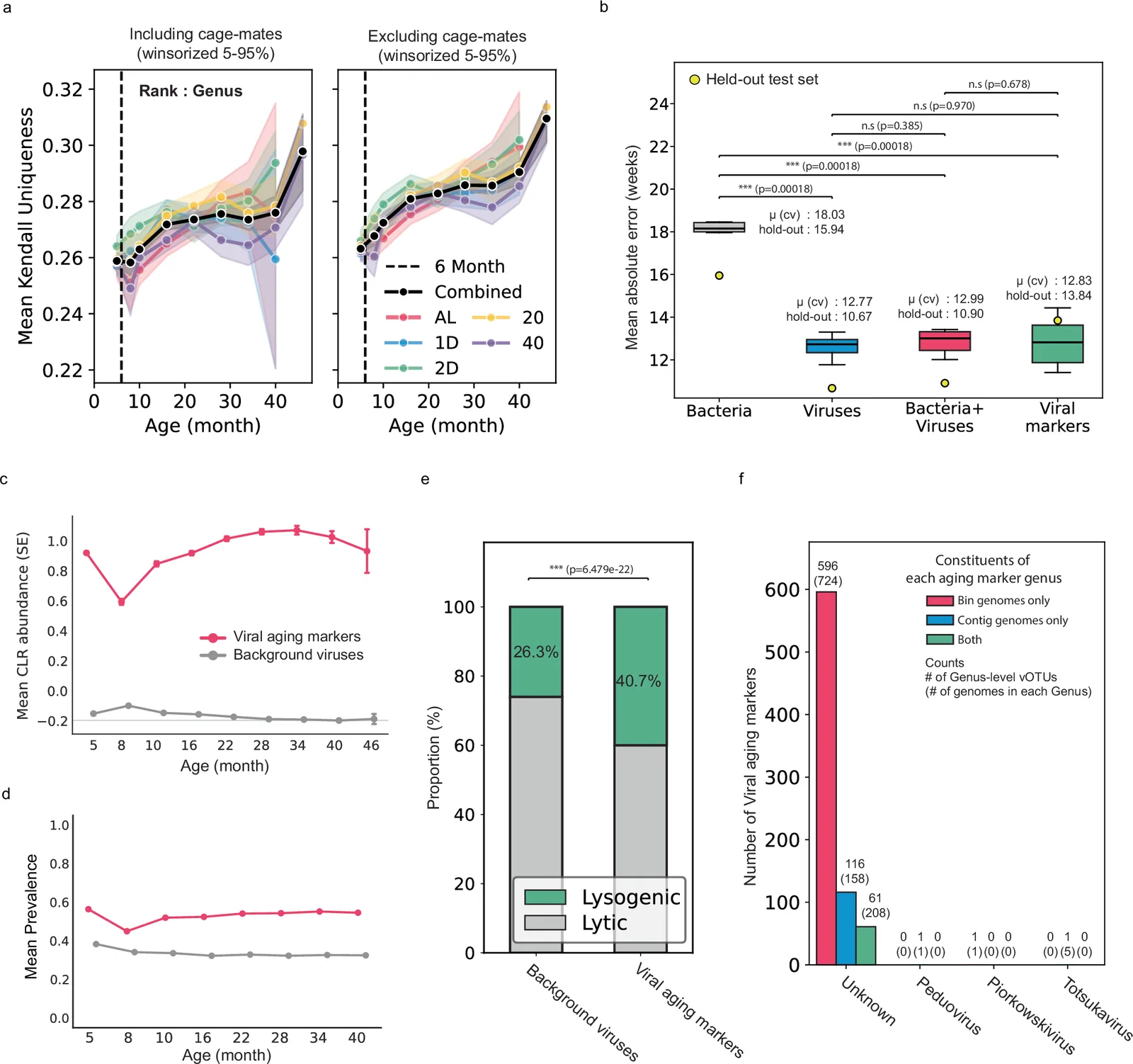
Gut viral ageing clock in mice. **a** Kendall uniqueness on samples including cage-mates (left) and excluding cage-mates (right). Shaded bands indicate 95% confidence intervals from 1000 bootstrap resamples. **b** Mice age predictions by 162 bacterial genera from the MGBC pangenome catalog (Bacteria), 5576 viral genera from MRGV (Viruses), 5738 bacterial and viral genera (Bacteria + Viruses), and 776 diet-independent ageing-associated viral genera (Viral markers) using XGBoost Regressor models. For boxplots, box lengths represent the interquartile range of the data (IQR), and whiskers extend to 1.5x IQR. The center bar represents the median. Statistical significance was assessed using a two-tailed Mann-Whitney U test (n.s. non-significant, *** *P* < 0.001). **c**, **d** Mean CLR abundance (**c**) and Mean prevalence (**d**) distribution across mouse age groups between 776 viral ageing markers and 4800 non-significant (Background) viral genera. Error bars indicate mean ± SE across samples within that age group. **e** Proportional difference of lysogenic and lytic lifecycle between 776 viral ageing markers and 4800 background viruses. Significance of difference was assessed using two-tailed Fisher’s Exact Test (*** *P* < 0.001). **f** Proportions of 776 viral marker genera composed of bin-level, contig-level, or both types of genomes. Unknown group includes all viral genera that could not be assigned to the current ICTV-ratified taxonomy.

We first assessed whether the gut virome exhibits progressive individualization during ageing. Kendall uniqueness (**Methods**) increased steadily across the lifespan (Fig. 6a), indicating growing virome individuality with age. This pattern became more pronounced after excluding cage-mates, confirming that divergence reflects intrinsic biological ageing rather than shared environment or batch effects. Notably, a similar age-associated increase in uniqueness was previously observed for the gut bacteriome in the same cohort, suggesting coordinated diversification of viral and bacterial communities during ageing. This synchronous divergence indicates a fundamental coupling between host ageing and coordinated restructuring of the gut microbiome.

We next evaluated age prediction using genus-level vOTUs with an XGBoost regression framework (**Methods**). The virome-based model yielded a significantly lower prediction error than the bacteriome-based age prediction model (MAE: 10.67 vs. 15.94 weeks; p = 0.00018) (Fig. 6b; Supplementary Data 11). Integrating bacterial and viral features yielded negligible improvement (MAE = 10.90 weeks), indicating that the dominant ageing signal is captured by the viruses. Moreover, a model based on 776 diet-independent viral ageing markers (**Methods;** Supplementary Data 12) retained the majority of age-predictive signal (MAE = 13.84 weeks), demonstrating that specific viral lineages serve as robust, diet-independent biomarkers of biological ageing and can function as a non-invasive viral ageing clock.

### Temperate phages recovered by viral binning drive age prediction

To characterize the ecological identity of viruses driving age prediction, we analyzed the longitudinal dynamics of the 776 viral genera identified as ageing markers. Tracking their prevalence and abundance across the lifespan revealed robust, monotonic trajectories rather than random fluctuations (Fig. 6c-d). A substantial subset showed consistent increases in both prevalence and relative abundance with age, effectively blooming in the older gut environment^36,66^. These directional patterns suggest that age-associated physiological changes, such as altered motility, immune senescence, or shifts in bacterial substrates, create ecological niches that favor specific viral lineages^67,68^. The reproducibility of these trends across individuals indicates deterministic ageing-related forces rather than stochastic drift.

We next assessed whether this expansion reflects a specific viral life-history strategy. Ageing markers were significantly enriched for temperate signatures compared with the background virome (40.7% vs. 26.3%; Fisher’s exact test, *P* < 6.48e-22; odds ratio = 1.92; Fig. 6e).

Given that diverse bacterial lineages undergo compositional shifts with age due to the loss of colonization resistance and broader ecological remodeling of the gut microbiota^66,69^, their resident prophages may concomitantly expand. This pattern is consistent with “Piggyback-the-Winner” dynamics, whereby high host density favors temperate phage propagation through lysogen expansion or density-dependent induction^61^. Accordingly, the viral ageing clock likely captures amplified prophage signals from multiple age-shifting bacterial lineages, potentially serving as a sentinel of declining mucosal homeostasis.

Notably, 76.8% (596/776) of these predictive lineages were recovered exclusively through viral binning and would have been missed by single-contig assembly (Fig. 6f). Most ageing markers represented previously unclassified viral genera, underscoring that the viral ageing clock is largely driven by previously uncharacterized viral diversity comprising genomes inaccessible to single-contig approaches. Together, these results highlight the importance of metagenomic viral binning for recovering biologically informative viral genomes and characterizing viral dynamics linked to host ageing.

## Discussion

In this study, we present MRGV, the comprehensive genomic catalog of the mouse gut virome to date, comprising 109,778 vMAGs with ≥50% completeness, including 3810 complete and 32,618 high-quality genomes (>90% completeness), representing 28,824 species-level vOTUs. Approximately 67.6% of these species-level vOTUs have not been reported in previous mouse gut viral genome databases, demonstrating that MRGV markedly expands the known taxonomic diversity of the mouse gut virome. Nevertheless, rarefaction analyses indicate that species- and genus-level viral diversity remains far from saturation, highlighting the vast unexplored mouse gut virosphere.

We observed limited overlap between mouse and human gut viromes, with only 6.91% of species-level vOTUs and 9.4% of protein clusters shared with UHGV, indicating pronounced taxonomic and functional divergence. This divergence mirrors recent observations in the gut bacteriome, where bacterial community composition also differs substantially between mice and humans^7^. In this study, the parallel age-associated increase in individuality observed for both viral and bacterial communities further supports the notion that bacteriomes and viromes co-evolve within host-specific ecological contexts. Together, these results suggest that, just as caution is required when extrapolating gut bacterial findings from mouse models to humans, similar care is necessary when translating observations of the gut virome across host species.

A key methodological advance of this study is the systematic incorporation of viral genome binning. By rescuing over 280,000 contigs that would otherwise have been discarded, viral binning contributed 39,467 additional vMAGs, accounting for ~36% of the catalog and increasing average genome completeness by nearly 60%. This approach enabled effective recovery of large and jumbo phage genomes exceeding 200 kb, demonstrating that viral binning is essential for capturing the full ecological and genomic diversity of the virosphere. The biological importance of viral genome binning was further exemplified by the viral ageing clock analysis, in which the vast majority of predictive viral ageing markers were derived from binned genomes.

Despite extensive functional annotation using structure-based approaches, more than half of the MRGV proteins remain unannotated, reflecting a substantial reservoir of functional “dark matter” in the gut virosphere. This highlights the limitations of homology-based annotation and underscores the need for complementary strategies, such as interaction-based or context-aware approaches, to resolve the functions of orphan viral proteins.

Despite this functional dark matter, the subset of annotated MRGV proteins revealed lineage-specific functional signatures within mouse gut Crassvirales, providing a genomic framework for interpreting variation in host breadth and predicted lifestyle among its lineages. Steigviridae was enriched for lysis- and host-impact-associated functions, including endolysin, toxin, and transcriptional regulator annotations, while showing little evidence of integrase-related modules. This pattern is consistent with a predominantly lytic genomic profile and agrees with previous observations that Steigviridae are frequently recovered under plaque-based experimental conditions and often encode elevated tRNA repertoires, which may support productive infection and partial translational autonomy during replication^70–72^.

In contrast, Intestiviridae and the Zeta subtype showed enrichment of integrase, excisionase, CI-like repressor, and transcriptional regulatory modules, suggesting a stronger association with temperate infection strategies. Such modules are central to lysogeny establishment, maintenance, and induction in diverse phage systems^73–76^. The concurrent enrichment of anti-restriction, replication-support, and structural genes in Intestiviridae may help explain its broad host range by supporting persistence across diverse host backgrounds while retaining the capacity for productive replication. Similarly, the enrichment of nucleic-acid processing genes, homing endonucleases, and reverse transcriptase in the Zeta subtype suggests increased genome-remodeling potential, which may contribute to lineage diversification and host-range flexibility^77,78^.

The Epsilon subtype showed a contrasting pattern, with enrichment of host-recognition genes, particularly tail fiber annotations, together with fewer genome-plasticity-associated functions. Because tail fiber proteins are major determinants of host adsorption and specificity in tailed phages^79^, this pattern is compatible with a more host-specialized ecological profile. The presence of amidase, transcriptional repressor, and nuclease annotations further suggests that Epsilon phages may retain efficient infection and replication functions within a narrower host niche^80^. Together, these findings support a model in which mouse gut Crassvirales lineages differ along a continuum from lytic-associated specialists to temperate broad-host-range lineages and host-specialized subtypes. Nevertheless, these lifestyle assignments are based on genome-derived functional signatures and should be validated in future studies using isolate-based infection assays, prophage induction experiments, or in situ virus–host linkage approaches.

This study has several limitations. First, although conservative criteria were applied, viral binning inherently carries the risk of generating chimeric genomes, particularly for jumbo phages that are highly fragmented and lack close reference genomes. Currently, no standardized framework exists to rigorously assess chimerism in viral bins, emphasizing the need for improved validation methodologies. Second, while we controlled host genetics, cage, batch effects, and diet-specific signals in ageing analyses, the complex interplay between host factors and virome restructuring remains incompletely resolved. Future studies should systematically examine how longevity-promoting interventions, such as dietary restriction, modulate host–virus interactions and whether attenuating age-associated viral shifts contributes causally to lifespan extension. Finally, MRGV primarily represents the gut virome of laboratory mice; expanding sampling to wild mice with diverse diets and environments will be essential to obtain a more comprehensive view of the mouse gut virosphere.

In conclusion, we present MRGV, a comprehensive mouse gut virome catalog that substantially expands known viral diversity through viral genome binning. The improved prediction of host ageing using gut phage features highlights the potential of virome-informed models for metagenome-based inference of host phenotypes. Collectively, MRGV establishes a robust reference framework that will facilitate future studies of mammalian gut virome and their interactions with host biology and disease.

## Methods

### Whole-metagenome sequencing (WMS) data

A total of 2638 WMS datasets from mouse fecal samples (20 newly generated and 2618 retrieved from public repositories) were used for metagenomic assembly and subsequent putative viral contig identification. The collated public datasets are summarized in Supplementary Data 1. In addition to the publicly available data, 20 fecal samples from C57BL/6J mice (6 weeks old; Orient Bio, Korea) treated with polystyrene nanoplastics were newly analyzed in this study. Mice were housed under controlled conditions (22 ± 2 °C, 55 ± 5% humidity, 12 hours light/dark cycle) and provided a standard chow diet (Harlan Teklad 2019S; Harlan Teklad, USA) and drinking water ad libitum, as described in the original study^81^. As all animals were male, sex was not considered as a variable in the study design or analysis. All animal experiments were reviewed and approved by the Institutional Animal Care and Use Committee of the Korea Food Research Institute (approval number: KFRI-M-22046). For each sample, 100 ng of DNA was fragmented using a Covaris LE200 focused ultrasonicator to generate blunt-ended DNA fragments. Fragments of approximately 350 bp were size-selected, ligated with indexing adapters, and amplified by eight cycles of PCR. Library quality and size distribution were assessed using D1000 ScreenTape on an Agilent TapeStation 4200. Libraries were prepared according to the TruSeq Nano DNA library Preparation Guide and quantified using KAPA Library Quantification Kits for Illumina sequencing platforms, following the qPCR Quantification Protocol Guide (Kapa Biosystems, catalog #KK4854). The libraries were sequenced on an Illumina HiSeq 4000 system, generating 12-47 Gbp of sequence data per sample.

### Metagenomic contigs assembly and putative viral contig identification

Adapters and low-quality bases were removed from raw sequence reads using Trimmomatic (v0.39)^82^, followed by host genome decontamination by aligning reads to the mouse reference genome GRCm39 (GenBank assembly accession GCA_000001635.9) with bowtie2^83^. High-quality reads were assembled into contigs primarily using MetaSPAdes (v3.15.3)^18^, with MEGAHIT (v1.2.9)^19^ employed as an alternative assembler. Putative viral sequences were identified from the assembled contigs using DeepVirFinder (v1.0)^20^ and VIBRANT (v1.2.1)^21^, together with Phigaro (v2.3.0)^22^, a tool specially designed for prophage sequence detection. Putative viral contigs predicted by DeepVirFinder were further filtered using a virus score ≥0.95 and a *P*-value ≤ 0.01. This filtering yielded 1,643,931, 1,707,417, and 96,944 putative viral contigs identified by DeepVirFinder, VIBRANT, and Phigaro, respectively. All predicted viral contigs were then deduplicated within each sample by clustering contigs using the UCLUST algorithm at 100% average nucleotide identity (ANI) and 100% alignment fraction (AF) with Vclust (v1.3.0)^84^, resulting in a total of 3,005,381 non-redundant putative viral contigs.

### Filtering and validation of high-confidence viral contigs

The quality of the 3,005,381 non-redundant viral contigs was assessed using CheckV (v1.0.1; database v1.5)^85^, followed by systematic decontamination. Host-flanking regions were first removed, and contigs encoding more host-derived genes than viral genes were excluded based on CheckV annotations. To further remove cellular contamination, BUSCO was searched against bacterial BUSCO (v5)^86^ HMM profiles (bacteria_odb10) using hmmsearch, and contigs with a BUSCO ratio (number of BUSCO hits / total predicted genes) > 5% were discarded. In addition, integrative and conjugative element (ICE) sequences were removed using a neural network classifier adapted from Camarillo-Guerrero et al.^87^, which distinguishes phage sequences from ICEs based on gene count, proportion of hypothetical proteins, and 5-mer relative frequency. After these filtering steps, 1,903,101 viral contigs were retained.

These contigs were subsequently reclassified using geNomad (v1.5.0)^23^ and VirRep (v1)^24^. Contigs were retained if they satisfied both a viral score >0.7 and FDR < 0.05 in geNomad, or a viral score >0.7 in VirRep. Viral contigs meeting the following additional criteria were designated as contig-level viral metagenome-assembled genomes (vMAGs): (1) genome length > 5 kbp; (2) k-mer frequency <1.3; (3) complete or high-quality with high confidence for lytic viruses; (4) complete, high-, or medium-quality with high confidence for lysogenic viruses; (5) viral prediction by at least three of the five tools (DeepVirFinder, VIBRANT, Phigaro, geNomad and VirRep); and (6) absence of CheckV warnings indicating either “1.5× longer than expected genome length” or “no viral genes”. This yielded 48,840 initial contig-level vMAGs, comprising 40,274 high-confidence putative lysogenic genomes classified as complete, high-, or medium-quality, and 8566 high-confidence lytic genomes classified as complete or high-quality.

The remaining 1,854,261 contigs that did not meet the initial contig-level quality control (QC) criteria were further filtered prior to binning using the following criteria: (1) genome length >2 kbp; (2) k-mer frequency <1.5; (3) viral prediction by at least two tools; and (4) not flagged as a prophage by any of the four tools (Phigaro, VIBRANT, geNomad, and CheckV). This yielded 1,190,038 contigs for viral binning.

Because CheckV is not optimal to estimate contamination on multi-contig fasta, host flanking regions in each 1,190,038 contigs were manually removed based on CheckV host coordinates, and the contamination returns 0 after rerunning CheckV on the decontaminated 1,190,038 contigs before viral binning. Viral binning was performed using MetaBat2^25^, SemiBin2^26^ and vRhyme^27^ with default parameters, generating 251,630, 134,874, and 291,378 redundant bins, respectively, each containing at least two contigs. Contig sequencing depth was calculated using the *jgi_summarize_bam_contig_depths* module in MetaBAT2 following sample-wise read alignment to contigs with Bowtie2. Bin-level validation was conducted using normalized geNomad and VirRep scores, calculated as length-weighted averages across contigs within each bin. Bins satisfying all of the following criteria were retained: (1) geNomad viral score >0.7; (2) geNomad FDR < 0.05; (3) geNomad maximum universal bacterial single-copy genes <2; (4) geNomad minimum viral hallmark genes >1; (5) geNomad viral hallmark gene enrichment >1.5; and (6) VirRep viral score >0.7. This step retained 677,882 bins. Multiple contigs within each viral bin were concatenated with a 50-bp spacer of “N” characters between adjacent contigs prior to CheckV quality assessment. To prevent the spacer regions from biasing the quality metrics, we introduced minor modifications to the CheckV source code: (i) gene prediction was performed with Prodigal using the -m option, which prevents gene calls spanning runs of “N” characters; and (ii) genome length was recomputed by subtracting the total spacer length [(50 bp) × (number of contigs − 1)] from the concatenated sequence length. Final bin-level QC required genome length >5 kbp, k-mer frequency <1.3, and CheckV quality ≥ medium quality with high confidence, resulting in 114,320 redundant viral bins. To eliminate redundancy across binning tools, sample-wise bin consolidation was performed using UPGMA (Unweighted Pair Group Method with Arithmetic Mean) clustering based on the number of shared contigs as a distance metric. Representative bins were selected using a composite quality score defined as: *(Completeness − contamination* − *k-mer frequency + number of viral genes* + *1e* − *4 × genome size)*. This consolidation produced 39,467 non-redundant bin-level vMAGs comprising 287,782 contigs.

Bins failing final QC (563,562 bins containing 892,256 contigs) were disassembled back into individual contigs. To rescue putative medium-quality lytic viral genomes, contig-level filtering criteria were reapplied, yielding an additional 21,471 unbinned contig-level vMAGs.

In total, the Mouse Reference Gut Virome (MRGV) comprises 109,778 vMAGs, including 70,311 contig-level vMAGs and 39,467 bin-level vMAGs. The contig-level vMAGs consist of 48,840 genomes retained from the initial contig-level QC, including high-confidence putative lysogenic vMAGs classified as complete (707), high-quality (12,810), or medium-quality (26,757), and high-confidence putative lytic vMAGs classified as complete (2447) or high-quality (6119). Viral binning yielded 39,467 bin-level vMAGs, all classified as putative lytic, including complete (656), high-quality (13,689), and medium-quality (25,122) genomes. In addition, contig rescue from QC-failed bins recovered 21,471 medium-quality contig-level vMAGs, all classified as putative lytic (Supplementary Data 2-3).

Importantly, the thresholds and quality criteria applied throughout this study were largely derived from, or informed by, previous large-scale virome studies^12–17^ and were intentionally chosen to be conservative, prioritizing high confidence and minimizing false-positive viral genome recovery.

### Viral operational taxonomic unit (vOTU) clustering and taxonomic annotation

Clustering and taxonomic delineation of vOTUs were performed using a framework largely informed by criteria and procedures established in a previous large-scale gut virome study^13^. UPGMA clustering was applied to define species-, genus-, and family-level vOTUs. Species-level vOTUs were delineated using a threshold of 95% ANI and 85% AF, calculated with skANI (v0.1.4)^88^ using the options -c 30, -m 300, --robust, and --learned-ani. Representative genomes for each species-level vOTU were selected based on a composite quality score. Using this approach, 28,824 species-level vOTUs were identified from the 109,778 MRGV vMAGs.

To define thresholds for higher taxonomic ranks, pairwise average amino acid identity (AAI) and gene-sharing percentages were computed among 17,766 ICTV^28^ reference genomes from VMR_MSL40.v1 using DIAMOND (v2.0.15)^89^. Statistical performance of UPGMA clustering was systematically evaluated across combinations of AAI and gene-sharing cutoffs (Supplementary Data 13). For all genome pairs, true positives were defined as genome pairs from the same ICTV rank assigned to the same cluster, whereas false positives were defined as genome pairs from different ranks assigned to the same cluster; true negatives and false negatives were defined conversely. Optimal cutoffs for genus- and family-level delineation were selected based on the F1-score. Applying the selected thresholds to the 28,824 species-level representative genomes resulted in 11,578 genus-level vOTUs clustered using 38.5% AAI and 49.5% gene sharing (recall = 0.878; F1-score = 0.808), and 2629 family-level vOTUs clustered using 16% AAI and 25% gene sharing (recall = 0.820; F1-score = 0.845).

Taxonomic names were assigned to MRGV vOTUs by comparing the 109,778 MRGV genomes with 17,766 ICTV reference genomes. Species-level assignments were based on ANI and AF computed using skANI, while higher-rank assignments relied on AAI and gene-sharing metrics computed with DIAMOND. A taxonomic name was assigned to a cluster when at least 70% of its member genomes supported the same taxonomy.

For vOTUs without matches in ICTV VMR_MSL40.v1, genomic distances to viral sequences in the Unified Human Gut Virome (UHGV)^9^ were estimated at each taxonomic rank using the *uhgv-classify* module, with BLASTN replaced by skANI. Although ICTV-based assignments were prioritized, UHGV-based taxonomy was adopted when it provided a lower (more resolved) taxonomic rank and was concordant with the lowest common rank inferred by both methods. As a result, 85,682 genomes were classified at Class, 18 were at Order, 11,590 were at Family, 12,472 were at Genus, 15 were at species, and 1 genome was not classified (Supplementary Data 6)

### Rarefaction analysis of MRGV taxonomy clusters

Rarefaction analysis was implemented with 28,824 species-, 11,578 genus-, and 2629 family-level vOTUs. The multi-clusters were defined when a cluster had at least 2 genomes. The number of observed clusters was calculated by averaging the observed cluster number of 1000 random permutations of 100 genomes. The Heaps’ law and Asymptotic model^90^ were fitted using curve_fit module from python3 SciPy (v1.15.3) (Supplementary Data 5). Heaps’ law coefficient quantifies the rate at which new vOTUs are discovered with increasing vMAGs, with lower values indicating saturation and higher values indicating continued accumulation of rare taxa. The percentage of theoretical saturation was calculated by dividing the number of observed clusters by the number of theoretical maximum clusters that was estimated from the Asymptotic model on each taxonomic rank.

### Protein prediction and functional annotation of mouse gut viral genomes

Functional annotation of viral genomes was performed using a combination of sequence- and structure-based approaches implemented in Pharokka (v1.7.5)^91^ and Phold (v0.2.0)^39^, respectively. Pharokka predicted protein-coding sequences (CDSs) from viral genomes using PHANOTATE (v1.5.1)^92^ and all predicted proteins were subjected to homology-based annotation by alignment against the PHROG (v4)^40^, CARD (v3.2.8)^93^, and VFDB (downloaded on 19 January 2024)^94^ databases using MMseqs2 (v17)^95^ with Pharokka’s default search parameters: -e 1E-05 -s 8.5 for PHROGs, and –min-seq-id 0.8 -c 0.4 -s 8.5 for CARD and VFDB (--cov-mode 0 applied by MMseqs2 default). Non-coding functional elements were identified in parallel: tmRNA and tRNA were predicted using ARAGORN (v1.2.41)^96^ and tRNAscan-SE (v1.4)^97^, respectively, and CRISPR spacers were detected using minCED (v0.4.2)^98^.

For structure-based functional annotation, 3Di structural representations were generated using ProstT5 (v0.0.1)^99^, and structural alignment was performed with Foldseek^38^ against the Phold database with integrates PHROG, CARD, VFDB, DefenseFinder (available at January 31, 2024)^100^, acrDB^101,102^ and Netflax^103^. In total, 8,212,891 predicted viral proteins were hierarchically clustered into homologous groups using MMseqs2 linclust^95^ at multiple identity thresholds (90%, 70%, 50% and 30% AAI) with a minimum AF of 80% (--min-seq-id 0.9/0.7/0.5/0.3 with MMseqs2 default -c 0.8 –cov-mode 0 applied). This procedure yielded 954,585, 746,733, 652,176, and 625,774 protein clusters at the respective identity levels.

### Comparison of gut viral protein families between humans and mice

A total of 25,452,549 gut viral protein sequences from UHGV MQ plus genomes were retrieved and hierarchically clustered using MMseqs2 linclust at 90%, 70%, 50%, and 30% AAI (--min-seq-id 0.9/0.7/0.5/0.3 with MMseqs2 default -c 0.8 –cov-mode 0 applied), yielding 2,677,869, 1,646,052, 1,295,052, and 1,175,763 protein clusters, respectively. Protein clusters lacking PHROG annotations were subsequently annotated using Phold to improve functional coverage.

To quantify overlap at the gut viral protein family level between humans and mice, representative protein sequences from the mouse gut virome (625,774 proteins clustered at 30% AAI from MRGV) and the human gut virome (1,175,763 proteins clustered at 30% AAI from UHGV) were pooled and reclustered using MMseqs2 linclust with a 30% identity threshold AAI (--min-seq-id 0.3 with MMseqs2 default -c 0.8 –cov-mode 0 applied). The resulting shared and host-specific clusters were used to assess conservation and divergence of gut viral protein families across host species.

### Inference of lysogenic and lytic viral lifestyle

The lysogenic lifestyle of 109,778 MRGV viral genomes was inferred based on the presence of lysogeny-associated evidence. First, viral genomes integrated into their host genomes were assigned to the lysogenic lifecycle by computing ANI and AF between MRGV viral genomes and MRGM bacterial genomes using skANI (options: -c 30, -m 300, --learned-ani, --robust) and filtering with 99% ANI and 99% AF, resulting in a total of 65,269 lysogenic viral genomes.

For viral genomes without integrated evidence, lifestyle were classified as lysogenic if it satisfied at least two of the following three criteria: (1) prediction as a prophage by at least one of four viral detection tools (CheckV, VIBRANT, Phigaro, or geNomad); (2) presence of an integrase gene; (3) presence of integration-associated accessory genes, including excisionase and transcriptional regulator, excisionase, site-specific recombination directionality factor (RDF), or Cox-like excisionase and repressor, with additional 6709 viral genomes assigned to lysogenic. The remaining 37,800 viral genomes without integration and not meeting the above additional criteria were classified as lytic lifecycle. Collectively, a total of 71,978 viral genomes were classified as lysogenic, and 37,800 were classified as lytic lifecycle (Supplementary Data 7).

### Comparison of gut virome diversity

MRGV vOTUs (*n* = 28,824) were compared with 977 hcMGV and 1645 mqMGV vOTUs^10^ and 168,536 UHGV vOTUs^9^ using skANI (v0.1.4). To avoid overestimating the number of MRGV-unique vOTUs, an MRGV vOTU was considered overlapping with an external vOTU when at least one of all genomes, including the representative and its member genomes, satisfied an ANI ≥ 95% and aligned fraction ≥ 85% cutoff against that external vOTU.

### Metagenomic sequencing read alignment to genomic catalogs of gut virome

WMS data from 50 Diversity Outbred (DO) mouse samples (PRJNA1054518)^36^ and viral-like particle (VLP)-enriched sequencing data from 79 mouse gut samples (PRJEB32560)^37^ were downloaded (Supplementary Data 8) and preprocessed following the procedures described in the original studies. Preprocessed reads were aligned to representative vOTU genomes from three catalogs, MRGV (28,824 vOTUs), hcMGV^10^ (977 vOTUs), and UHGV^9^ (168,536 vOTUs), using Minimap2 (v2.28)^104^ with default parameters. To minimize spurious or ambiguous alignments (e.g., secondary, supplementary, or incorrectly oriented mappings), only primary, properly paired alignments were retained, corresponding to SAM flag pairs 99/147 and 163/83. Unique vOTUs within each catalog were defined as those for which none of the genomes belonging to the vOTU (including both representative and member genomes) showed overlap with genomes from other catalogs, based on species-level demarcation thresholds (<95% ANI or <85% AF). In contrast, shared vOTUs were defined as those in which at least one genome overlapped with a genome from another catalog at ≥95% ANI and ≥85% AF.

### Assignment of bacterial hosts for mouse gut viral genomes

Bacterial hosts for 109,778 MRGV viral genomes were inferred by integrating CRISPR spacer matching and k-mer-based detection of viral integration into bacterial genomes. First, 595,538 CRISPR spacers were extracted from 42,245 MRGM bacterial genomes using MinCED and aligned to the 109,778 MRGV viral genomes with BLASTN (BLAST+ v2.12.0; options: -dust no, -word_size 7)^105^. All spacer sequences are publicly available as described in the **Data Availability** section. From 54,181,500 initial hits, 9,541,946 high-confidence matches were retained by applying stringent filters: ≤1 mismatch, ≥95% sequence identity, ≥95% spacer alignment fraction, and gap openings <5% of spacer length. Second, integrated viral genomes were identified by computing ANI and AF between MRGV viral genomes and MRGM bacterial genomes using skANI (options: -c 30, -m 300, --learned-ani, --robust). Alignments meeting ≥99% ANI and ≥99% AF were retained, yielding 2,100,906 high-confidence alignments across 65,269 viral genomes integrated into 16,588 bacterial genomes. Finally, host assignments for vOTUs were determined by aggregating evidence across member genomes and assigning the lowest possible host taxonomic rank supported by ≥70% consensus. Using this approach, 22,758 vOTUs were assigned to bacterial hosts at least to the phylum level (Supplementary Data 9).

### Linkage evidence-based host breadth analysis

In total, 96,850 viral genomes spanning 22,758 vOTUs that were assigned to a bacterial host at least to the phylum level had at least one host connection via CRISPR spacer matching and/or prophage integration. To reliably classify the host breadth of each viral genome, a cross-rank host connection (Multiple genera, families, orders, classes, or phylum) was accepted only when the corresponding pair of host species at the boundary of that rank was independently linked by at least three distinct viral genomes via CRISPR spacer matching. Because integration evidence provides a direct sequence-level link between viral and host genomes, a single supporting viral genome was considered sufficient for an integration-based connection.

### Estimation of viral prevalence and abundance across mouse gut metagenomes

Short reads from 2638 mouse WMS datasets used for MRGV construction were aligned to the 109,778 MRGV genomes using Minimap2 (v2.28)^104^. Genome-level aligned coverage was calculated with CoverM (v0.7.0)^106^. To estimate viral prevalence across samples, only alignments covering more than 50% of the viral genome length were considered as positive detections. For abundance estimation, alignment counts were normalized by genome length, scaled by the total number of normalized counts per sample, and multiplied by 10^6, yielding relative abundance values comparable across samples.

### Phylogenetic analysis of MRGV species-level vOTUs

To infer evolutionary relationships among 28,824 MRGV species-level vOTUs, terminase large subunit (TerL) genes were used as phylogenetic markers. Representative TerL sequences were extracted from 22,679 vOTUs. When a TerL sequence was absent from the representative genome of a vOTU, the longest TerL sequence among its member genomes was selected instead. Multiple sequence alignment of the 22,679 TerL sequences was performed using MAFFT (v7.525)^107^ with the --localpair and --maxiterate 16 options. Alignment columns containing more than 50% gaps were removed using trimAL (v1.5)^108^. A phylogenetic tree was then constructed using FastTree (v2.1.11)^109^ under the WAG + Γ model with the -mlacc 2 and -slownni options. The resulting tree was visualized using iTOL (v6)^110^.

### Phylogenetic validation of crAss-like viral genomes

A total of 1569 putative crAss-like viral genomes were initially classified using the *uhgv-classify* module. For phylogenetic validation, TerL sequences were extracted from 76 and 631 reference crAssvirales genomes obtained from ICTV VMR_MSL40.v1 and Yutin et al.^51^, together with TerL sequences from the 1569 MRGV crAss-like viral genomes. All TerL sequences were aligned using MAFFT with the --localpair and --maxiterate 16 options, and alignment columns containing more than 50% gaps were removed using trimAL. Phylogenetic relationships were inferred using IQ-TREE (v2.4.0)^111^ under the Q.pfam+F+I+G4 model, with the best-fit model selected using -m TEST and branch support assessed by 1000 ultrafast bootstrap replicates (-bb 1000) and 1000 SH-aLRT tests (-alrt 1000). Based on phylogenetic placement within established crAssvirales clades, 1382 of the 1569 putative crAss-like viral genomes were validated.

### Functional profiling of PHROG categories across crAssvirales subtypes

All annotated proteins belonging to seven PHROG functional categories (DNA/RNA and nucleotide metabolism, connector, transcription regulation, tail, lysis, integration and excision, and moron/auxiliary metabolic gene/host takeover) were extracted from each crAssvirales subtype. For each subtype, gene counts were normalized by the number of genomes, yielding gene-per-genome values. Within each PHROG category, the top three most abundant genes were selected across subtypes. Gene-level z-scores were then computed based on normalized gene-per-genome values and visualized using a clustermap generated with the Seaborn v0.13.2 library in Python 3.

### Estimation of viral taxonomic abundance in DO mice

A total of 2997 fecal samples were collected from 913 DO mice^36^ (Supplementary Data 10). Viral taxonomic abundance profiles at the vOTU level were estimated using Kraken v2.1.3^65^ and Bracken v.3.0.1^64^ with a custom database constructed from the 28,824 MRGV vOTUs. Prior to Bracken-based abundance estimation, vOTUs were filtered by requiring >10% genome coverage, defined as the ratio of unique minimizers to total minimizers. Genus-level viral abundance profiles were then calculated by summing genome length-normalized counts of the filtered vOTUs assigned to each genus, followed by multiplicative centered log-ratio (CLR) transformation resulting in 5576 viral genera detected. For downstream comparative analyses to the mouse bacteriome, mouse bacteriome kraken2 database was constructed by 26,640 high-quality mouse pangenomes from MGBC^112^, upon which the 2997 mouse bacteriome abundances were estimated using Kraken v2.3.1 and Bracken v3.0.1, followed by 10% coverage filtering and transforming to multiplicative CLR values identical to virome profiling.

### Quantification of microbiome uniqueness and ageing clock modeling

Quantification of microbiome uniqueness and age prediction followed the analytical framework of the original DO mouse study^36^. Microbiome uniqueness across the 2997 DO mouse samples was quantified using Kendall’s tau, computed with *kendalltau* from SciPy (v1.15.3). Pairwise Kendall’s tau correlations were calculated for all sample pairs, and the minimum Kendall’s tau value for each sample was defined as its Kendall uniqueness, representing maximal dissimilarity from all other samples.

Age prediction was performed on all 2997 samples using an XGBoost regressor (v3.0.5). The dataset was partitioned into training (80%) and test (20%) sets, with an additional validation set comprising 10% of the training data for model tuning. To prevent data leakage, samples from the same mouse (unique mouse ID) and the same cage (unique cage ID) were assigned exclusively to a single split (training, validation, or test), and age distributions were balanced across splits using StratifiedGroupKFold from scikit-learn (v1.7.2). Model hyperparameters were optimized using a greedy search strategy. To mitigate overfitting, feature subsampling was applied, retaining up to 40% of features when the total number exceeded 2000, and up to 80% otherwise.

### Identification of diet-independent viral ageing markers

The 776 viral ageing markers were identified through the following procedure. First, MaAsLin2 (v1.8.0)^113^ was applied to all 2997 mouse samples, with age specified as a fixed effect and mouse ID, cohort, cage ID, and sequencing batch included as random effects. Genera with an absolute coefficient >0.3 and *q*-value < 0.01 were retained, yielding 803 age-associated viral genera. To exclude diet-specific ageing markers, MaAsLin2 was then run separately within each dietary subgroup using identical settings except that age was the only fixed effect. The four diet groups comprised 1D (one fasting day per week; *n* = 584), 2D (two consecutive fasting days per week; *n* = 583), 20 (20% daily caloric restriction; *n* = 634), and 40 (40% daily caloric restriction; *n* = 623). This analysis identified 896, 791, 1089, and 1631 significant genera in the 1D, 2D, 20, and 40 groups, respectively (Supplementary Data 14). Diet-specific ageing markers were defined as genera that were significant in only a single dietary subgroup, resulting in 75, 51, 74, and 427 unique genera for the 1D, 2D, 20, and 40 diets, respectively. After excluding these diet-specific genera from the initial 803 age-associated genera, a final set of 776 diet-independent viral ageing marker genera was obtained (Supplementary Data 12).

### Statistics and Reproducibility

No statistical method was used to predetermine sample size; all available publicly accessible metagenomic datasets meeting the inclusion criteria were incorporated into the analyses. No data were excluded from the analyses. The experiments were not randomized, as this study consists entirely of computational analyses of existing metagenomic datasets without experimental intervention. The investigators were not blinded to allocation during experiments and outcome assessment, as this study involves computational analyses without experimental intervention.

### Reporting summary

Further information on research design is available in the Nature Portfolio Reporting Summary linked to this article.

## Supplementary information

Supplementary information

Description of Additional Supplementary Files

Supplementary Data S1-14

Reporting summary

Transparent Peer Review file

## Source data

Source data

## Data Availability

The 20 mice gut metagenomic data generated in this study have been deposited in the European Nucleotide Archive (ENA) database under accession code PRJNA1276045. All other raw metagenomic datasets analyzed in this study are publicly available under the accession codes listed in Supplementary Data 1. All MRGV-associated resources, including viral genomes, protein sequences, metadata, CRISPR spacers, and the Kraken2 database, are publicly accessible at https://zenodo.org/records/2011598910.5281/zenodo.20115989^114^. Source data are provided with this paper.

## Code availability

All custom scripts used for data processing, statistical analysis, and figure generation, together with the corresponding source data and trained prediction models, are publicly available at https://github.com/netbiolab/MRGV and archived at https://zenodo.org/records/2101225910.5281/zenodo.21012259^115^.
