# Supplementary material for: A genomic catalog of the mouse gut virome reveals features associated with ageing
Source: Nat Commun. 2026 Jul 21;17:8910. doi: 10.1038/s41467-026-75836-6 (PMC13503771; doi:10.1038/s41467-026-75836-6)
Supplement: Supplementary file 1 [file 41467_2026_75836_MOESM1_ESM.pdf]

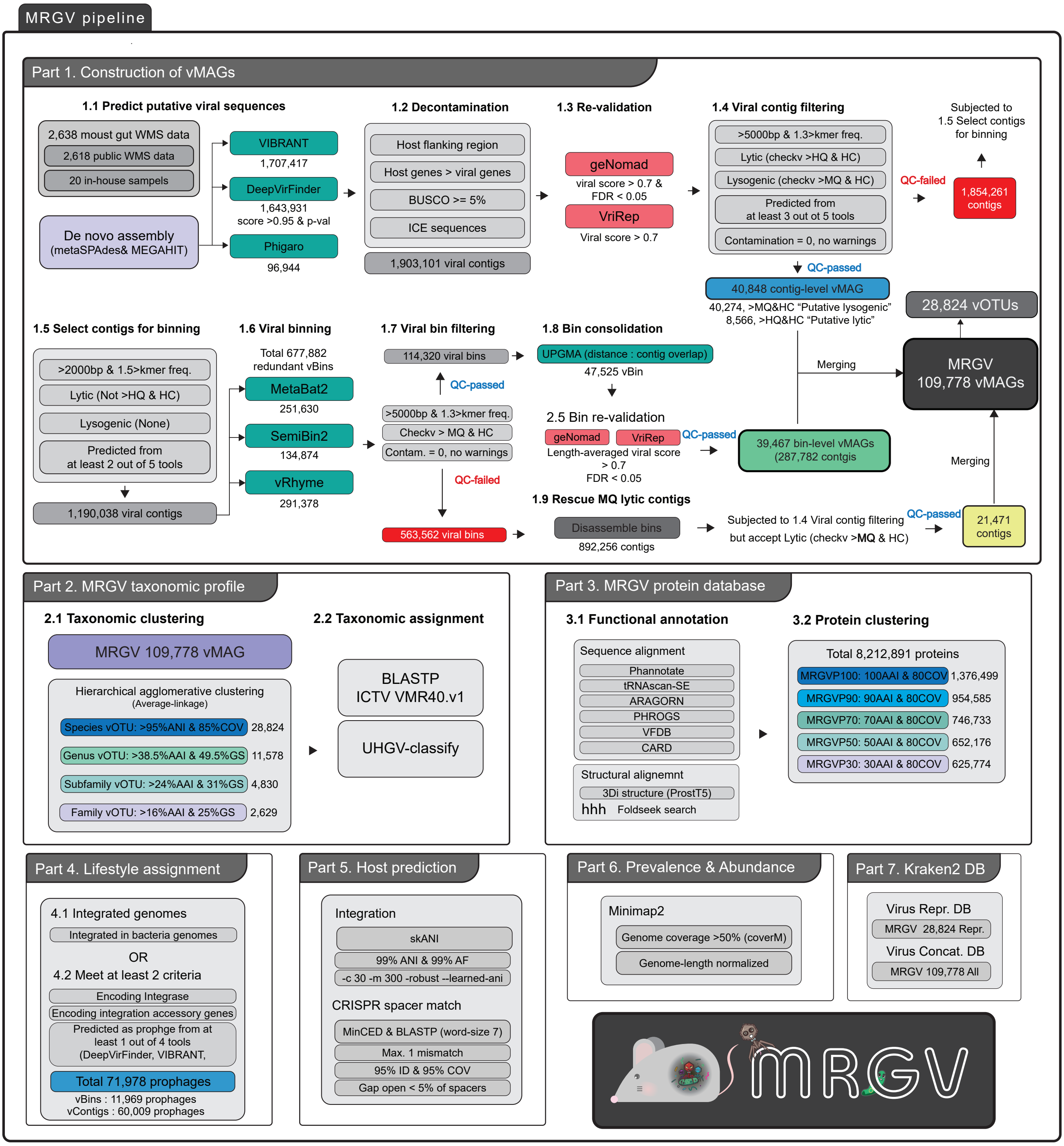

Supplementary Fig. 1 | Summary of MRGV construction pipeline. **(Part.1)** Construction of 109,778 MRGV high-confident vMAGs. **(Part.2)** Defining viral operational taxonomic units (vOTUs) and taxonomic annotations. Hierarchical agglomerative clustering with average linkage was applied to all viral taxonomic ranks. Viral taxonomic nomenclature was assigned based on ANI (species-level) or AAI (genus, subfamily, and family-level) according to ICTV VMR.v1 and uhgv-classify module. **(Part.3)** Functional profiling workflow. All CDS were structurally aligned to PHROG database using Phold. Protein clusters of MRGV were hierarchically clustered from 100, 90, 70, 50 and 30% of AAI using MMseqs2. **(Part.4)** Workflow to assign the lifestyle of viruses. **(Part.5)** Host-virus linkage analysis. **(Part.6)** Viral prevalence and abundance analysis workflow. **(Part.7)** Construction of MRGV kraken2 database. MRGV Repr. DB was built with only 28,824 MRGV representative vOTU genomes, and MRGV Concat. DB was built with 109,778 MRGV genomes
