# Supplementary material for: A genomic catalog of the mouse gut virome reveals features associated with ageing
Source: Nat Commun. 2026 Jul 21;17:8910. doi: 10.1038/s41467-026-75836-6 (PMC13503771; doi:10.1038/s41467-026-75836-6)
Supplement: Supplementary file 2 [file 41467_2026_75836_MOESM2_ESM.pdf]

Supplementary Data Legends

File Name: Supplementary Data 1

Description: Accession information for all 2,638 mouse gut whole-metagenome sequencing (WMS) datasets used for MRGV construction, including study, sample, run, and experiment accessions.

File Name: Supplementary Data 2

Description: Summary statistics of the 109,778 MRGV viral genomes, categorized by recovery stage (contig-level or bin-level), assembly level, putative lifecycle (lytic or lysogenic), and CheckV quality classification (complete, high-quality, or medium-quality).

File Name: Supplementary Data 3

Description: Genome-level metadata for all 109,778 MRGV viral genomes, including CheckV quality classification, MIUVIG quality tier, completeness and contamination estimates, completeness inference method, k-mer frequency, species-level vOTU assignment, putative lifecycle, recovery stage, and assembly level.

File Name: Supplementary Data 4

Description: Taxonomic clustering assignments for all 109,778 MRGV viral genomes at three taxonomic ranks: species-level vOTUs (28,824), genus-level vOTUs (vGENs; 11,578), and family-level vOTUs (vFAMs; 2,629). For each rank, the cluster identifier, cluster size, and cluster type (singleton or multi-member) are provided.

File Name: Supplementary Data 5

Description: Results of rarefaction curve fitting for species-, genus-, and family-level vOTUs using Heaps' Law and Asymptotic models. Parameters reported include the Heaps' Law exponent (gamma) and coefficient (kappa), the asymptotic maximum (A), the asymptotic rate (b), and the estimated percent saturation (%) at each taxonomic rank.

File Name: Supplementary Data 6

Description: Taxonomic annotations for all 109,778 MRGV viral genomes based on comparison with 17,766 ICTV reference genomes from VMR_MSL40.v1, including genome identifier, species-level vOTU assignment, and assigned ICTV taxonomy.

File Name: Supplementary Data 7

Description: Lifestyle classification (lytic or lysogenic) for all 109,778 MRGV viral genomes, including genome identifier, species-level vOTU assignment, inferred lifestyle, and supporting lysogeny-associated evidence used for classification.

File Name: Supplementary Data 8

Description: Accession metadata for the 50 bulk WMS datasets (BioProject PRJNA1054518) and 79 VLP-enriched sequencing datasets (BioProject PRJEB32560) used for taxonomic profiling benchmarking.

File Name: Supplementary Data 9

Description: Bacterial host assignments for 22,758 MRGV species-level vOTUs inferred by integrating CRISPR spacer matching and prophage integration evidence. For each vOTU, the assigned bacterial host taxonomy from the Mouse Reference Gut Microbiome (MRGM; R220), consensus support level, and type of supporting evidence (CRISPR only, integration only, or both) are provided.

File Name: Supplementary Data 10

Description: Accession metadata for all 2,997 fecal samples from 913 Diversity Outbred (DO) mice used for virome-based ageing clock analysis, including study, sample, run, and experiment accessions.

File Name: Supplementary Data 11

Description: Age prediction performance metrics for virome-based, bacteriome-based, and combined models, including mean absolute error (MAE) from 10-fold cross-validation (10CV) and held-out test sets, stratified by data type, dietary group, prediction stage, feature type (viral genus or bacterial genus), taxonomic rank, and cage-controlled conditions.

File Name: Supplementary Data 12

Description: A total of 776 diet-independent viral ageing marker genera and 27 diet-unique ageing-associated genera (803 significant genera in total) identified by MaAsLin2. Statistical analysis was performed using MaAsLin2 (multivariable linear mixed-effects model, two-sided), with age as a fixed effect and mouse ID, cohort, cage ID, and sequencing batch as random effects. Coef, coefficient (effect size); Stderr, standard error of the coefficient; P value, unadjusted p value; Q value, BH-adjusted p value (Benjamini-Hochberg correction for multiple comparisons). Significance thresholds: $|Coef| > 0.3$ and Q value < 0.01.

File Name: Supplementary Data 13

Description: Performance evaluation of taxonomic clustering at genus and family ranks using 17,766 ICTV representative species genomes, showing the top 10 highest F1-score parameter combinations per rank. Metrics reported include AAI cutoff, gene-sharing cutoff, sensitivity, precision, specificity, F1-score, accuracy, negative predictive value (NPV), adjusted Rand index (ARI), adjusted mutual information (AMI), and Fowlkes-Mallows score (FM-score).

File Name: Supplementary Data 14

Description: Age-associated viral genera identified by MaAsLin2 within each dietary subgroup (1D: one fasting day per week; 2D: two consecutive fasting days per week; 20: 20% daily caloric restriction; 40: 40% daily caloric restriction), including MRGV genus identifier, metadata variable, effect value, diet group, degree of overlap across diet groups, number of diet groups in which the genus was significant, and diet-unique classification. Statistical analysis was performed using MaAsLin2 (multivariable linear mixed-effects model, two-sided), with age as the sole fixed effect within each dietary subgroup.
