# Supplementary material for: A genomic catalog of the mouse gut virome reveals features associated with ageing
Source: Nat Commun. 2026 Jul 21;17:8910. doi: 10.1038/s41467-026-75836-6 (PMC13503771; doi:10.1038/s41467-026-75836-6)
Supplement: Supplementary file 3 [file 41467_2026_75836_MOESM4_ESM.pdf]

nature portfolio

Corresponding author(s): *Double-anonymous peer review submissions: write DAPR and your manuscript number here instead of author names.*

Last updated by author(s): *YYYY-MM-DD*

Reporting Summary

Nature Portfolio wishes to improve the reproducibility of the work that we publish. This form provides structure for consistency and transparency in reporting. For further information on Nature Portfolio policies, see our Editorial Policies and the Editorial Policy Checklist.

Statistics

For all statistical analyses, confirm that the following items are present in the figure legend, table legend, main text, or Methods section.

n/a	Confirmed	
☐	☒	The exact sample size (*n*) for each experimental group/condition, given as a discrete number and unit of measurement
☐	☒	A statement on whether measurements were taken from distinct samples or whether the same sample was measured repeatedly
☐	☒	The statistical test(s) used AND whether they are one- or two-sided<br>*Only common tests should be described solely by name; describe more complex techniques in the Methods section.*
☐	☒	A description of all covariates tested
☒	☐	A description of any assumptions or corrections, such as tests of normality and adjustment for multiple comparisons
☐	☒	A full description of the statistical parameters including central tendency (e.g. means) or other basic estimates (e.g. regression coefficient) AND variation (e.g. standard deviation) or associated estimates of uncertainty (e.g. confidence intervals)
☐	☒	For null hypothesis testing, the test statistic (e.g. *F*, *t*, *r*) with confidence intervals, effect sizes, degrees of freedom and *P* value noted<br>*Give P values as exact values whenever suitable.*
☒	☐	For Bayesian analysis, information on the choice of priors and Markov chain Monte Carlo settings
☒	☐	For hierarchical and complex designs, identification of the appropriate level for tests and full reporting of outcomes
☒	☐	Estimates of effect sizes (e.g. Cohen's *d*, Pearson's *r*), indicating how they were calculated

Our web collection on statistics for biologists contains articles on many of the points above.

Software and code

Policy information about availability of computer code

Data collection	Fastq files for 20 fecal samples of C57BL/6J mouse were generated on Illumina Hiseq 4000 system.
Data analysis	Read processing and assembly: Trimmomatic v0.39, Bowtie2, MetaSPAdes v3.15.3, MEGAHIT v1.2.9.<br>Viral identification and QC: DeepVirFinder v1.0, VIBRANT v1.2.1, Phigaro v2.3.0, Vclust v1.3.0, CheckV v1.0.1 (database v1.5), BUSCO v5 (bacteria_odb10), geNomad v1.5.0, VirRep v1, Prodigal. Viral binning: MetaBAT2 v2, SemiBin2 v2, vRhyme.<br>Clustering/taxonomy: skANI v0.1.4, DIAMOND v2.0.15, uhgv-classify module.<br>Functional annotation: Pharokka v1.7.5, PHANOTATE v1.5.1, Phold v0.2.0, MMseqs2 v17, ARAGORN v1.2.41, tRNAscan-SE v1.4, minCED v0.4.2, ProstT5 v0.0.1, Foldseek. Host linkage: BLASTN (BLAST+ v2.12.0), skANI v0.1.4.<br>Read mapping/abundance: Minimap2 v2.28, CoverM v0.7.0, Kraken2 v2.1.3/v2.3.1, Bracken v3.0.1. Phylogenetics: MAFFT v7.525, trimAL v1.5, FastTree v2.1.11, IQ-TREE v2.4.0, iTOL v6.<br>Statistics/modeling: Python 3 with SciPy v1.15.3, Seaborn v0.13.2, scikit-learn v1.7.2, XGBoost v3.0.5, MaAsLin2 v1.8.0.

For manuscripts utilizing custom algorithms or software that are central to the research but not yet described in published literature, software must be made available to editors and reviewers. We strongly encourage code deposition in a community repository (e.g. GitHub). See the Nature Portfolio guidelines for submitting code & software for further information.

Data

Policy information about availability of data

All manuscripts must include a data availability statement. This statement should provide the following information, where applicable:

- Accession codes, unique identifiers, or web links for publicly available datasets
- A description of any restrictions on data availability
- For clinical datasets or third party data, please ensure that the statement adheres to our policy

Data availability
The 20 mice gut metagenomic data generated in this study have been deposited in the European Nucleotide Archive (ENA) database under accession code PRJNA1276045 [https://www.ebi.ac.uk/ena/browser/view/PRJNA1276045]. All other raw metagenomic datasets analyzed in this study are publicly available under the accession codes listed in Supplementary Table 1. All MRGV-associated resources, including viral genomes, protein sequences, metadata, CRISPR spacers, and the Kraken2 database, are publicly accessible at https://zenodo.org/records/20115989 (DOI: 10.5281/zenodo.20115989).

Code availability
All custom scripts used for data processing, statistical analysis, and figure generation, together with the corresponding source data and trained prediction models, are publicly available at https://github.com/netbiolab/MRGV and archived at https://zenodo.org/records/21012259 (DOI: 10.5281/zenodo.21012259)

Research involving human participants, their data, or biological material

Policy information about studies with human participants or human data. See also policy information about sex, gender (identity/presentation), and sexual orientation and race, ethnicity and racism.

Reporting on sex and gender	Not applicable.
Reporting on race, ethnicity, or other socially relevant groupings	Not applicable.
Population characteristics	Not applicable.
Recruitment	Not applicable.
Ethics oversight	Not applicable.

Note that full information on the approval of the study protocol must also be provided in the manuscript.

Field-specific reporting

Please select the one below that is the best fit for your research. If you are not sure, read the appropriate sections before making your selection.

☒ Life sciences ☐ Behavioural & social sciences ☐ Ecological, evolutionary & environmental sciences

For a reference copy of the document with all sections, see nature.com/documents/nr-reporting-summary-flat.pdf

Life sciences study design

All studies must disclose on these points even when the disclosure is negative.

Sample size	No statistical methods were used to predetermine sample size. The 20 newly generated fecal samples were obtained from male C57BL/6J mice (6 weeks old, >20 g; n = 10 per group: vehicle and nanoplastic-treated). These samples were incorporated to expand metagenomic diversity for catalog construction rather than for group-level statistical comparisons; the available sample size was considered sufficient in the context of the larger 2,638-sample dataset. For catalog construction, all 2,638 mouse gut WMS datasets (20 newly generated and 2,618 publicly available) were included to maximize recovery of viral diversity; rarefaction analysis confirmed that family-level vOTU accumulation approached saturation while species- and genus-level diversity remained unsaturated, indicating the dataset captures the dominant higher-rank diversity while continued sampling would recover additional rare taxa. For taxonomic-profiling benchmarking, sample size was determined by the availability of publicly accessible evaluation cohorts (50 WMS and 79 VLP-enriched mouse gut samples); all available samples were used without subsampling. For the aging clock, all 2,997 longitudinal fecal samples from 913 Diversity Outbred mice in the source cohort were used; this is the complete published cohort and was not subsampled.
Data exclusions	No sample-level exclusions were applied. Viral contigs, bins, and genomes were filtered using pre-established, conservative quality criteria defined prior to analysis and described in full in the Methods (e.g., CheckV quality and contamination thresholds, geNomad/VirRep viral scores and FDR, k-mer frequency, minimum genome length, multi-tool prediction agreement, and removal of bacterial/MGE contamination). For abundance estimation, genomes were retained only above a pre-specified ≥50% genome-coverage threshold to minimize false positives. These criteria were applied uniformly and were informed by previous large-scale virome studies.
Replication	This study is based on computational analysis of existing metagenomic datasets; no wet-lab experiments were performed by the authors, and thus conventional replication in the experimental sense is not applicable. Reproducibility is ensured through the following measures: (1) all custom scripts, trained models, and source data are publicly available at GitHub (https://github.com/netbiolab/MRGV) and archived at Zenodo

(DOI: 10.5281/zenodo.21012259); (2) all third-party tools were applied with explicitly stated parameters as described in the Methods; (3) the MRGV catalog and associated resources are publicly available at Zenodo (DOI: 10.5281/zenodo.20115989), enabling independent replication of all analyses. For the 20 newly generated fecal samples, library preparation and sequencing were performed once per sample; no technical replicates were generated, as the primary use of these samples was catalog construction rather than experimental hypothesis testing.

Randomization	For the 20 newly generated fecal samples, male C57BL/6J mice were randomly allocated into two groups (n = 10 per group: vehicle and nanoplastic-treated) following a one-week adaptation period. Mice were orally gavaged with polystyrene nanoplastics (0.5 mg/day) or saline for 28 days. For all other datasets used in this study, randomization is not applicable as they are computational analyses of publicly available metagenomic data.
Blinding	Blinding was not applicable to this study, as all analyses were computational. For the 20 newly generated fecal samples, group allocation (vehicle vs. nanoplastic-treated) was known during analysis; however, as these samples were used solely for catalog construction rather than group-level comparisons, blinding was not required.

Reporting for specific materials, systems and methods

We require information from authors about some types of materials, experimental systems and methods used in many studies. Here, indicate whether each material, system or method listed is relevant to your study. If you are not sure if a list item applies to your research, read the appropriate section before selecting a response.

Materials & experimental systems

n/a	Involved in the study	
☒	☐	Antibodies
☒	☐	Eukaryotic cell lines
☒	☐	Palaeontology and archaeology
☐	☒	Animals and other organisms
☒	☐	Clinical data
☒	☐	Dual use research of concern
☒	☐	Plants

Methods

n/a	Involved in the study	
☒	☐	ChIP-seq
☒	☐	Flow cytometry
☒	☐	MRI-based neuroimaging

Animals and other research organisms

Policy information about studies involving animals; ARRIVE guidelines recommended for reporting animal research, and Sex and Gender in Research

Laboratory animals	Twenty fecal samples were newly generated from male C57BL/6J mice (6 weeks old, >20 g; Orient Bio, Korea) treated with polystyrene nanoplastics (BioProject PRJNA1276045). Mice were housed under controlled conditions (22 ± 2°C, 55 ± 5% humidity, 12 h light/dark cycle) with ad libitum access to standard chow (Harlan Teklad 2019S) and water. All remaining data were obtained from publicly available datasets listed in Supplementary Tables 1, 8, and 10.
Wild animals	The study did not involve wild animals.
Reporting on sex	All 20 newly generated samples were obtained from male C57BL/6J mice. Sex was not considered as a variable in the study design or analysis, as all animals were male and these samples were used solely for catalog construction. Sex-disaggregated analysis was therefore not applicable. Sex information for all publicly available datasets used in this study was not systematically collected or analyzed.
Field-collected samples	The study did not involve samples collected from the field.
Ethics oversight	All animal procedures for the 20 newly generated samples were reviewed and approved by the Institutional Animal Care and Use Committee of the Korea Food Research Institute (approval number: KFRI-M-22046). All publicly available datasets used in this study were collected under ethical oversight as described in their respective primary publications.

Note that full information on the approval of the study protocol must also be provided in the manuscript.

Plants

Seed stocks	Not applicable.
Novel plant genotypes	Not applicable.
Authentication	Not applicable.
