# Supplementary material for: A genomic catalog of the mouse gut virome reveals features associated with ageing
Source: Nat Commun. 2026 Jul 21;17:8910. doi: 10.1038/s41467-026-75836-6 (PMC13503771; doi:10.1038/s41467-026-75836-6)
Supplement: Supplementary file 4 [file 41467_2026_75836_MOESM5_ESM.pdf]

nature portfolio

Peer Review File

A genomic catalog of the mouse gut virome reveals features associated with ageing

Corresponding Author: Professor Insuk Lee

This file contains all reviewer reports in order by version, followed by all author rebuttals in order by version.

Version 0:

Reviewer comments:

Reviewer #1

(Remarks to the Author)
The manuscript presents a large-scale mouse reference database for gut virome, comprising more than 100,000 gut viromes and substantially expanding the known diversity of the mouse gut virome through the incorporation of viral genome binning. Building on this catalog, the authors perform a series of downstream analyses, including structure-based protein annotation and host linkage inference. The study further explores lineage-specific features of crAss-like phages and the potential of virome-based features for host age prediction. Overall, the reference resource is valuable, the analyses are generally well executed, and most of the methods appear solid. However, several aspects could still be improved before publication.

Major comments

1.The authors removed prophage sequences prior to viral binning, which is a thoughtful and important step. However, the Methods section does not clearly describe how this was done. Was Phigaro the only tool used to identify prophages at this stage? This step is particularly important to reduce the risk of generating artificially long bins, including false-positive jumbo phages, from prophage-containing contigs derived from bacterial genomes. A more stringent filtering strategy may be warranted to remove potential prophage-containing contigs before binning. Tools such as geNomad, VirSorter, PHASTEST, or PIDE could also be considered for this purpose. If reanalysis is not feasible, this limitation at least deserves a more in-depth discussion.

2.The comparison of functional overlap between mouse and human gut viromes may be biased and should be interpreted more cautiously. The authors conclude that mouse and human gut viromes share very few functional terms. However, the current analysis may be strongly influenced by rare, low-abundance functional terms, which could obscure the broader functional similarities between the two viromes. The analysis should consider the abundance distribution of functional terms rather than treating all functions equally. In particular, abundant and recurrent viral functions may provide a more biologically meaningful basis for cross-host comparison than the total set of rare annotated terms.

3.The viral aging clock would benefit from more stringent validation against environmental confounding. Although the authors partitioned samples by mouse ID to prevent repeated-sample leakage, this does not fully exclude shared cage effects, which may influence the gut virome and potentially inflate predictive performance. A leave-one-cage-out cross-validation analysis would provide a stronger test of robustness. Validation in an independent external cohort, if feasible, would further strengthen the conclusion.

4.The biological interpretation of the virome-based aging clock requires further discussion. A substantial proportion of the viral signal used for age prediction may originate from temperate phages integrated into bacterial genomes. Because prophages are often highly strain-specific, the improved predictive performance over bacterial composition may partly reflect increased strain-level resolution rather than a truly independent virome-specific effect. To provide a fairer comparison, the authors might consider combining bacterial and phage reference databases and quantifying both using the same framework, for example Kraken-based abundance estimation (like Phanta, PMID: 37231259), followed by parallel modeling. In addition, a recent study has reported increased prophage abundance in the aging human gut across multiple bacterial taxa (PMID: 41275070), not only facultative anaerobes and pathobionts, which may be related to changes in bacterial diversity and ecology (PMID: 26982729). It is therefore possible that age-associated enrichment of temperate phages contributes substantially to the predictive power of the viral aging clock, and this possibility should be discussed more explicitly.

5.The manuscript would also benefit from improved balance between the Results and Discussion. The Discussion section currently does not cite any references, which limits the depth and context of the interpretation. In contrast, some parts of the Results section contain extended speculative discussion. For example, Lines 360–398 correspond to a single figure but include several paragraphs of interpretive discussion that would fit better in the Discussion section. Rebalancing these sections would improve readability and strengthen the overall presentation.

Minor comments

1.The parameters used for MMseqs2 should be described more clearly in the Methods. MMseqs2 is used multiple times throughout the manuscript, but the relevant identity and coverage cutoffs are not always clearly stated. These parameters should be explicitly reported for each analysis.

2.The abbreviation "vOTU" is used throughout the main text before being formally defined in the Methods section. For clarity and readability, the authors should define "viral operational taxonomic unit (vOTU)" at its first appearance in the main text.

3.At Line 1252, Part 7 (Kraken2 DB) of Extended Data Fig. 1 is not mentioned in the figure caption and should be added for completeness.

(Remarks on code availability)

Reviewer #2

(Remarks to the Author)
The manuscript by Kim et al., entitled "Incorporating viral genome binning in a mouse gut virome catalog enables accurate age prediction", presented the Mouse Reference Gut Virome (MRGV), a comprehensive viral catalog that substantially expanded the currently known diversity of mouse gut viruses. The authors applied viral genome binning and structure-based annotation, which respectively improved estimated genome completeness and functional coverage. Furthermore, they reported that gut virome features predicted host ageing more accurately than bacterial features, with most predictive markers originating from binned viral genomes. These aspects constituted the principle novelty of this study. The comprehensive mouse gut virom catalog is a valuable contribution to the field. However, I have several comments regarding the current version of the manuscript.

Major comments:
1.According to the developers, CheckV is not optimally suited for multi-contig viral genomes (PMID: 33349699). CheckV can estimate completeness but not contamination for viral MAGs. Given this limitation, how were viral bins assessed to meet the reported criteria of "Contam. = 0, no warnings" and other CheckV-based thresholds (Extended Data Fig. 1; Part 1; 1.7, Viral bin filtering)?
2.The representative contigs of high-confidence mouse gut vOTUs (hcMGV, n = 977; misrepresented in Line 52 of this manuscript) were all at least 90% complete, as viral candidates with flanking bacterial regions, low completeness (< 90%) or CheckV warnings were removed during catalogue construction to ensure high reliability (PMID: 40542420). The same study also reported a set of medium-quality mouse gut vOTUs (mqMGV, 50% = < completeness < 90%, n = 1645), defined independently from hcMGV.
Given that the MRGV in the present manuscript (n = 28,824) included viral genomes with at least 50% completeness, it would be more appropriate to include hcMGV and mqMGV together, rather than hcMGV alone, in the comparisons shown in Fig. 2a, 2h, and 2i, to provide a more comprehensive and balanced reference to existing mouse gut virome resources.
In addition, clarification is needed for Fig. 2a. The authors reported that 23.05% of MRGV (n = 6,644) overlapped with 76.66% of hcMGV (n = 749). These percentages appeared asymmetric. Please reconcile and provide a rationale for the apparent discrepancy in counts.
3.The authors stated that 31.83%, 12.76%, 11.22%, and 19.36% of linked viruses respectively spanned multiple genera, families, orders, and even phyla. This conclusion requires better support. Are these connections supported by 1 spacer match? Given that the accuracy of CRISPR array prediction, especially in metagenomic data, is less than perfect, what are the chances that these matches are spurious? It is recommended to improve the reliability of these connections by increasing the threshold to multiple viruses (three or more) exhibiting connections with the two host species on both ends.

Minor comments:
Line 43, this ratio has been updated to phage: bacteria ~1:1 or 1:10 by many studies. A very old paper is being cited here. Please update.
How to distingish the lytic vMAG and lysogenic vMAGs?
1. Is there a specific rationale for selecting DeepVirFinder, VIBRANT, and Phigaro as the primary viral identification tools, with geNomad and VirRep applied as additional filters?
According to the developers, VirRep is dedicated to human gut metagenomic samples, the effectiveness of VirRep to other biomes could be further elevated by fine-tuning it on that biome-specific data (PMID: 38965579). Was any such fine-tuning performed before applying VirRep to mouse gut metagenomes in this study?
2. More details should be provided when describing the construction and analysis workflow.
3. L821: web portal (https://www.decodebiome.org/MRGV/) is not available.
4. Extended Data Fig. 1: Part 1, 1.5, "Lytic" instead of "Lityc"; Part 4, 4.2 "Meet" instead of "Meed"; Part 7, legend is missing.

(Remarks on code availability)

Version 1:

Reviewer comments:

Reviewer #1

(Remarks to the Author)
All my comments have been addressed.

(Remarks on code availability)

Reviewer #2

(Remarks to the Author)
This reviewer has carefully seen the responses made by the authors. All the concerns have been successfully addressed. Thanks for your efforts.

(Remarks on code availability)

Open Access This Peer Review File is licensed under a Creative Commons Attribution 4.0 International License, which permits use, sharing, adaptation, distribution and reproduction in any medium or format, as long as you give appropriate credit to the original author(s) and the source, provide a link to the Creative Commons license, and indicate if changes were made.
In cases where reviewers are anonymous, credit should be given to 'Anonymous Referee' and the source.
The images or other third party material in this Peer Review File are included in the article's Creative Commons license, unless indicated otherwise in a credit line to the material. If material is not included in the article's Creative Commons license and your intended use is not permitted by statutory regulation or exceeds the permitted use, you will need to obtain permission directly from the copyright holder.
To view a copy of this license, visit https://creativecommons.org/licenses/by/4.0/

Responses to referees (our responses in bold or colored text)

Manuscript ID: NCOMMS-26-007098-T

Attached please find our revised manuscript. We do appreciate the reviewers' critical but valuable comments, which have been a guideline for revising the manuscript. We believe that the revisions improved the manuscript, particularly in delivering our analysis to the readers clearly. We hope that our revised manuscript is suitable for the publication.

= = = = = = = = =

Reviewers' Comments:

Reviewer #1:

The manuscript presents a large-scale mouse reference database for gut virome, comprising more than 100,000 gut viromes and substantially expanding the known diversity of the mouse gut virome through the incorporation of viral genome binning. Building on this catalog, the authors perform a series of downstream analyses, including structure-based protein annotation and host linkage inference. The study further explores lineage-specific features of crAss-like phages and the potential of virome-based features for host age prediction. Overall, the reference resource is valuable, the analyses are generally well executed, and most of the methods appear solid. However, several aspects could still be improved before publication.

Major comments

Q1) The authors removed prophage sequences prior to viral binning, which is a thoughtful and important step. However, the Methods section does not clearly describe how this was done. Was Phigaro the only tool used to identify prophages at this stage? This step is particularly important to reduce the risk of generating artificially long bins, including false-positive jumbo phages, from prophage-containing contigs derived from bacterial genomes. A more stringent filtering strategy may be warranted to remove potential prophage-containing contigs before binning. Tools such as geNomad, VirSorter, PHASTEST, or PIDE could also be considered for this purpose. If reanalysis is not feasible, this limitation at least deserves a more in-depth discussion.

Response. We agree that the original Methods section did not sufficiently describe the prophage removal step, and we have revised the manuscript accordingly. Prophage identification was not restricted to Phigaro. We used four complementary tools in combination, namely Phigaro, VIBRANT, CheckV, and geNomad, which rely on related but non-identical algorithms for prophage and proviral region prediction. To minimize the risk of retaining prophage-containing contigs that could artificially elongate bins or generate false-positive predictions, we applied a deliberately conservative union-based criterion. Any contig flagged by at least one of the four tools was excluded prior to binning. This filtering workflow has now been described explicitly in the Methods *"Filtering and validation of high-confidence viral contigs"* as follows.

Line 585-586: ***"…and (4) not flagged as a prophage by any of the four tools (Phigaro, VIBRANT, geNomad, and CheckV)."***

Q2) The comparison of functional overlap between mouse and human gut viromes may be biased and should be interpreted more cautiously. The authors conclude that mouse and human gut viromes share very few functional terms. However, the current analysis may be strongly influenced by rare, low-abundance functional terms, which could obscure the broader functional similarities between the two viromes. The analysis should consider the abundance distribution of functional terms rather than treating all functions equally. In particular, abundant and recurrent viral functions may provide a more biologically meaningful basis for cross-host comparison than the total set of rare annotated terms.

Response. We thank the reviewer for raising this point and have re-examined our overlap analysis accordingly. Our additional analyses confirm that the 9.4% functional overlap reported in the main text is *not* an artifact of rare, low-abundance functional terms.

Choice of functional units. We deliberately did not adopt PHROG functional categories as the unit of analysis, because each PHROG category is a super-group encompassing a broad range of sub-functions and is therefore inappropriate as a single unit for assessing rareness. Instead, we used the 625,774 protein clusters defined at 30% sequence identity, each of which represents a more granular and biologically coherent functional unit suitable for prevalence stratification.

Definition of rareness. For each protein cluster (functional term) *f*, we defined a vOTU-size-normalized prevalence score as:

$$(1)\ prevalence\_score_f = \frac{1}{N_t}\sum_{v=1}^{N_t}\left(\frac{n_{f,v}}{G_v}\right)$$

$Where,$

$N_t: total\ number\ of\ MRGV\ vOTUs$

$n_{f,v}: number\ of\ genomes\ in\ vOTU\ v\ containing\ protein\ cluster\ f$

$G_v: total\ number\ of\ genomes\ in\ vOTU\ v$

Stratification by prevalence. We ranked all protein clusters by prevalence and identified breakpoints in the rank-prevalence curve using weighted segmented regression. Five candidate elbow points were detected; the two breakpoints showing the most pronounced changes in slope were selected (**Fig. Q2a**). These define three prevalence regions: a high-prevalence region, a transition zone, and a low-prevalence tail. As a sanity check, we confirmed that protein clusters with larger cluster sizes are preferentially located in the high-prevalence region (**Fig. Q2b**), consistent with these being broadly conserved viral functions.

Fig.Q2a

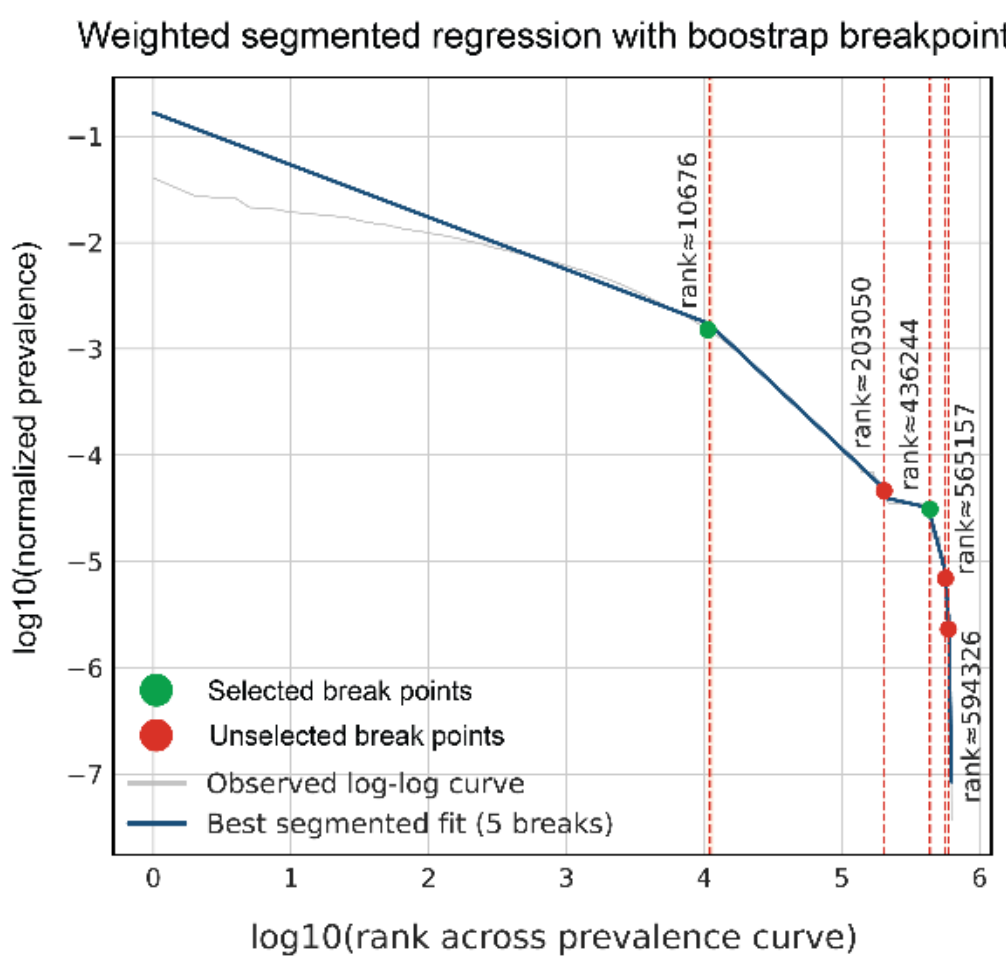

Fig.Q2b

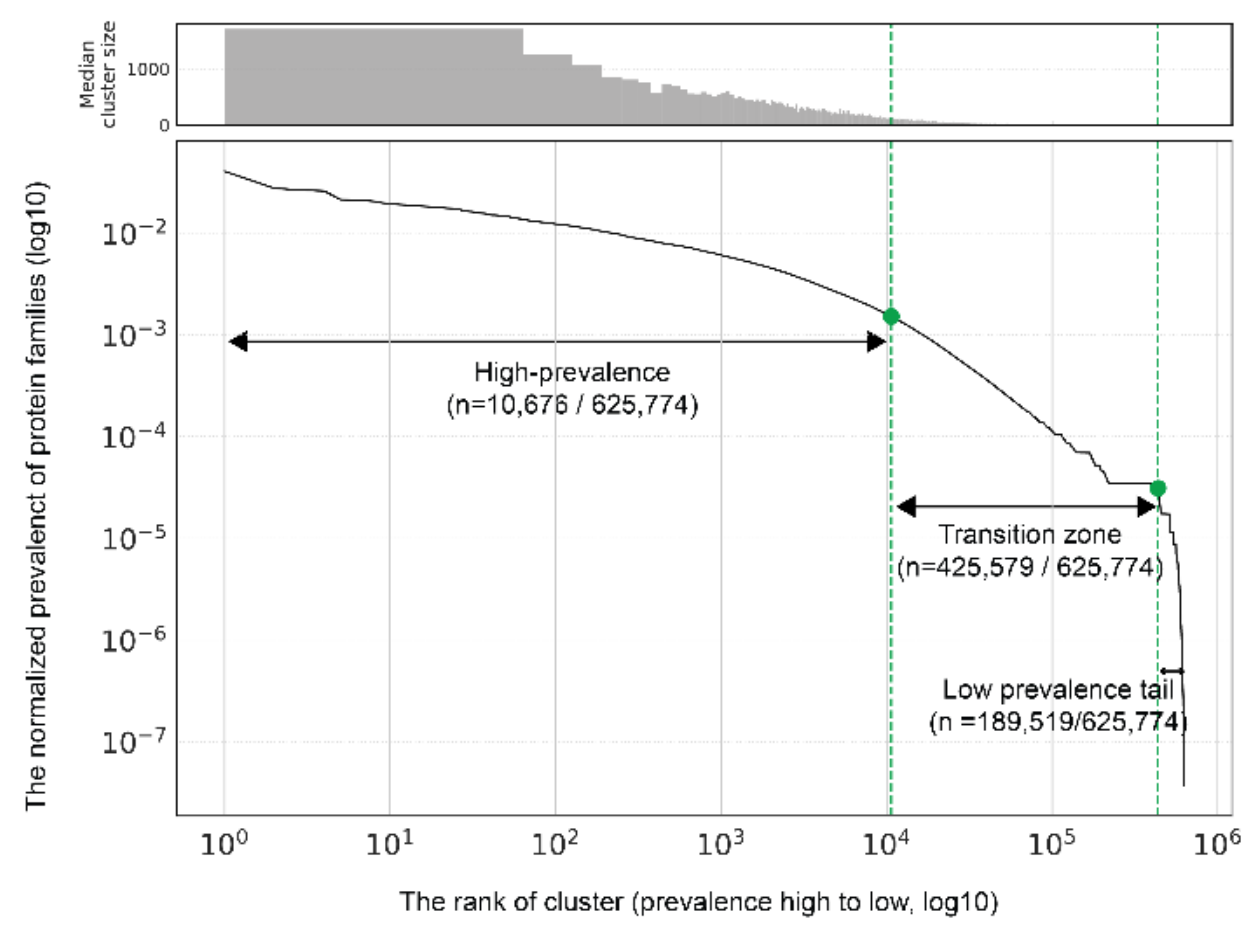

When **we compared the mouse and human gut viral protein clusters separately within each prevalence region**, we observed strong differences in cross-species overlap (**Fig. Q2c–d**). The **high-prevalence region, which represents the broadly conserved viral functional core** suggested by the reviewer as a more biologically meaningful basis for comparison, contained only 10,676 clusters, **corresponding to 1.7% of all 625,774 clusters**. Nevertheless, 33.2% of these clusters overlapped with human gut viral clusters (3,546 / 10,676), indicating that this region accounts for a substantial fraction of cross-species functional conservation. By contrast, **the transition zone, which contains medium-prevalence protein clusters and excludes the low-prevalence tail, comprised 425,579 clusters, corresponding to 68.0% of the total, but shows only 11.1% overlap with human gut viral clusters (47,177 / 425,579)**. This result is consistent with the low-overlap pattern reported in the main text.

Fig.Q2c

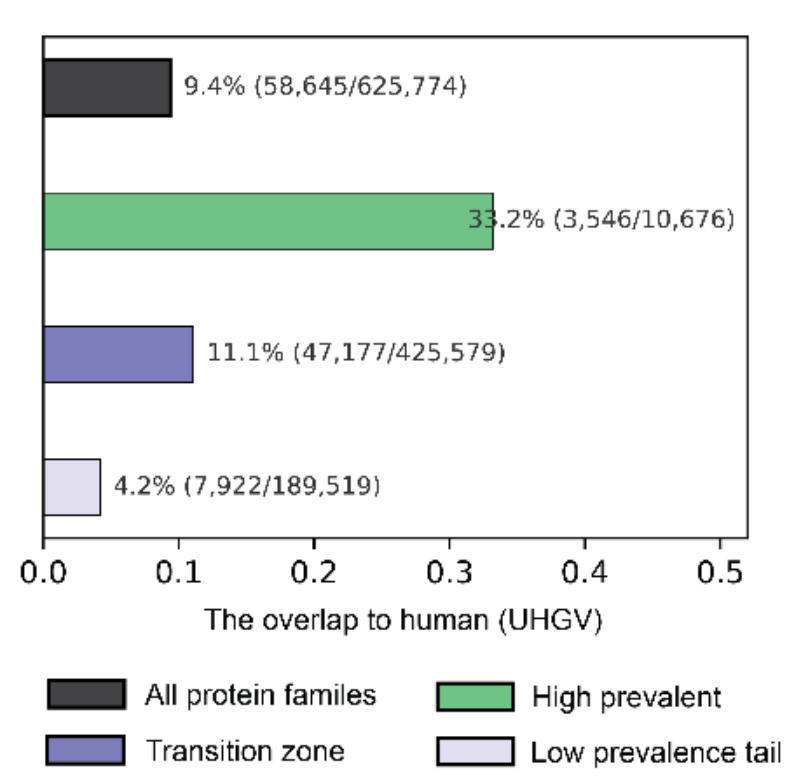

Fig.Q2d

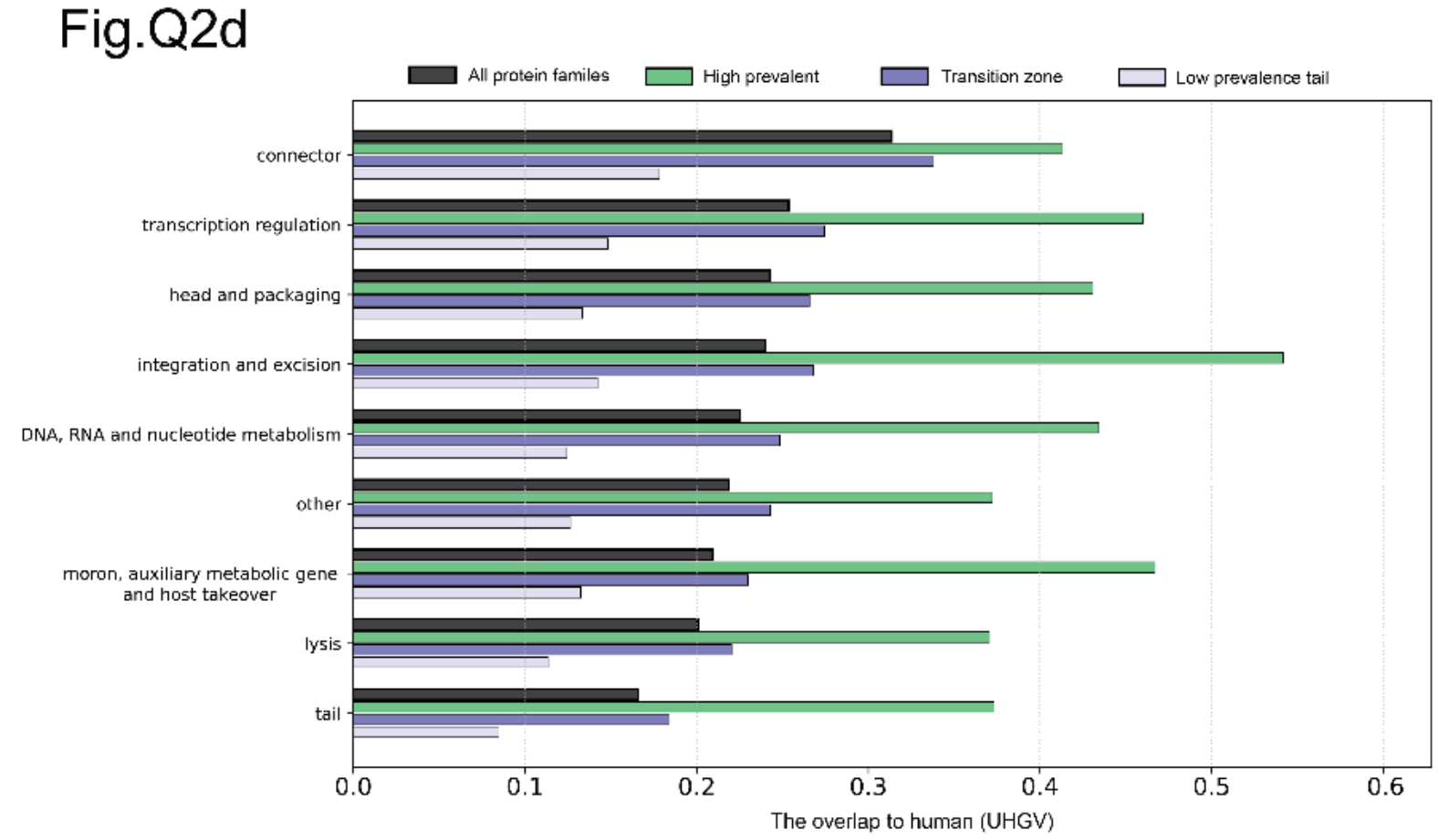

Taken together, these results indicate that the low cross-species overlap reported in the main text is not driven by bias from rare or low-abundance functional terms. **Broadly conserved, high-prevalence clusters do show higher cross-species overlap but they constitute only 1.7% of**

the catalog and cannot account for the overall trend. The remaining ~98%, ranging from the transition zone to the low-prevalence tail, consistently show low overlap, indicating that the reported pattern reflects a genuine biological feature of the cross-host comparison.

Q3) The viral aging clock would benefit from more stringent validation against environmental confounding. Although the authors partitioned samples by mouse ID to prevent repeated-sample leakage, this does not fully exclude shared cage effects, which may influence the gut virome and potentially inflate predictive performance. A leave-one-cage-out cross-validation analysis would provide a stronger test of robustness. Validation in an independent external cohort, if feasible, would further strengthen the conclusion.

Response. We thank the reviewer for this important suggestion and agree that incorporating cage as a grouping variable provides a more robust and conservative evaluation. We did not adopt strict leave-one-cage-out cross-validation because each of the 120 cages in our cohort contains on average ~25 samples (~0.8% of the dataset); leaving out a single cage per fold would yield validation sets too small for stable per-fold MAE estimates and would result in disproportionately large training sets that fail to reflect realistic generalization conditions. Instead, **we used cage-aware cross-validation** (StratifiedGroupKFold with cage as the group variable), preserving the conventional ~20% test partition size while **strictly preventing any cage from being shared between training and validation in any fold or in the holdout split**. This scheme provides the same cage-disjoint guarantee as leave-one-cage-out while retaining adequate statistical power per fold.

In parallel, to ensure methodological consistency with our viral abundance pipeline (relevant to Q4), we re-profiled bacterial abundance for all 2,997 DO mouse samples *de novo* using a Kraken2 database built from the MGBC pan-genome concatenation, rather than using the published bacterial abundance table from a prior study (PMID: 40164832). This ensures that both the bacterial and viral profiles in this manuscript are derived from internally consistent classifications.

After applying cage-aware cross-validation and the re-profiled bacterial abundance, we observed a significant decrease in predictive accuracy for the virus-only, bacteria+virus, and viral-marker models. However, the performance trend was preserved: the viral profile continued to yield significantly better age prediction than the bacterial profile (**Fig. Q3a**)

Fig.Q3a

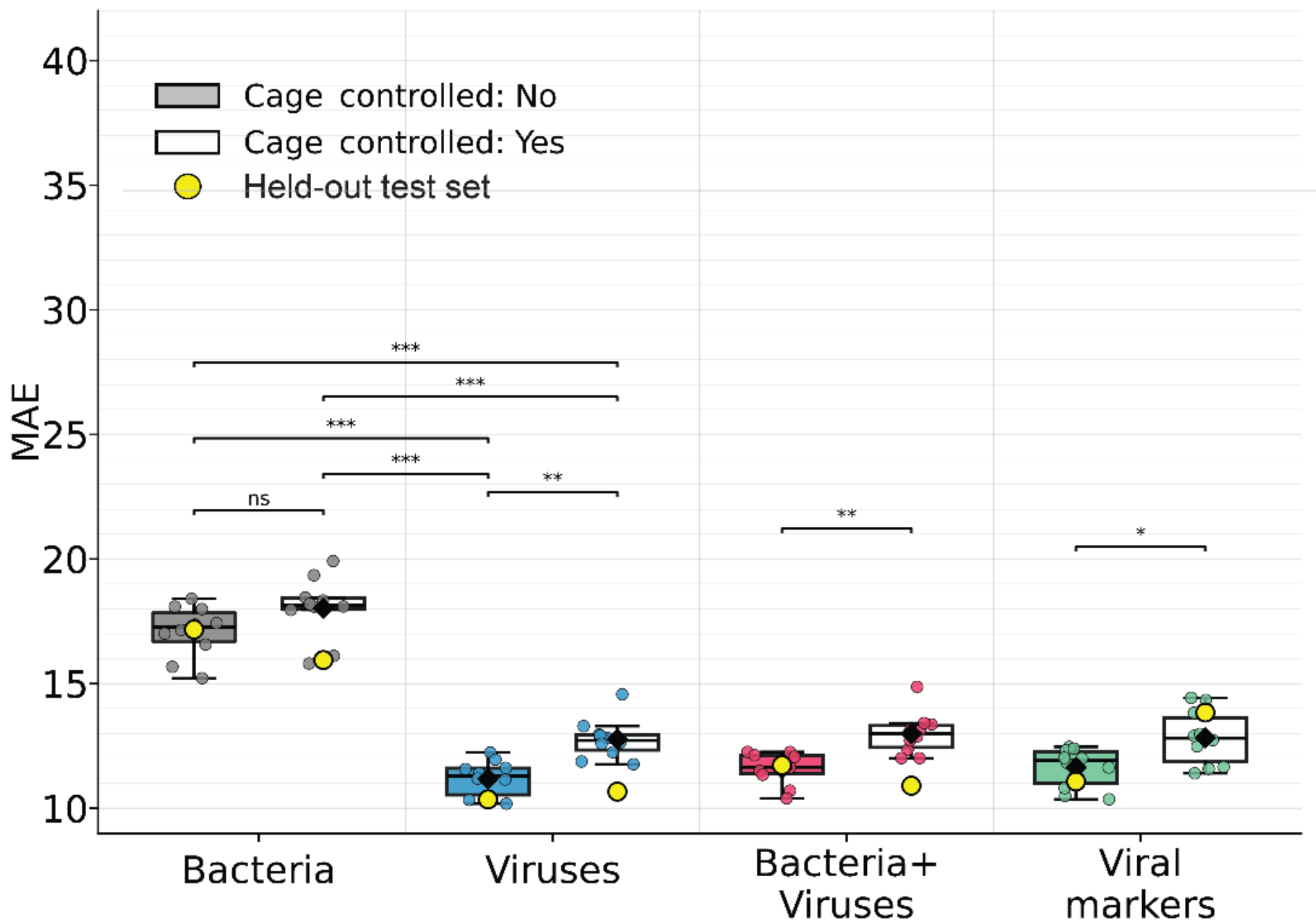

The change in the bacterial profile pipeline itself also affected bacterial-only model performance. Under cage-aware evaluation, the previously used bacterial abundance table yielded mean CV MAE = 16.96 / holdout MAE = 18.44 (**Fig. Q3b**), whereas the re-profiled MGBC-Kraken2 bacterial abundance yielded mean CV MAE = 18.03 / holdout MAE = 15.94 (**Fig. Q3c**). Accordingly, we have replaced the original **Main Figure 6b with Fig. Q3c** in the revised manuscript to reflect the methodologically consistent bacterial profile

Fig.Q3b

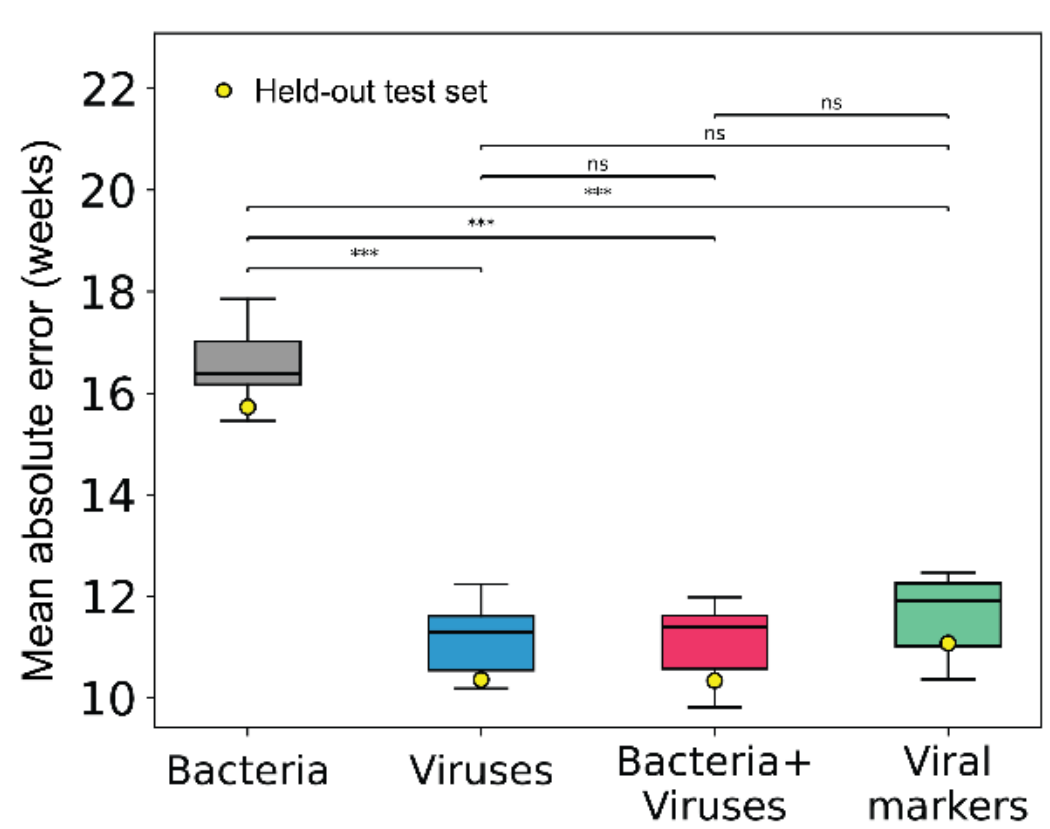

Fig.Q3c

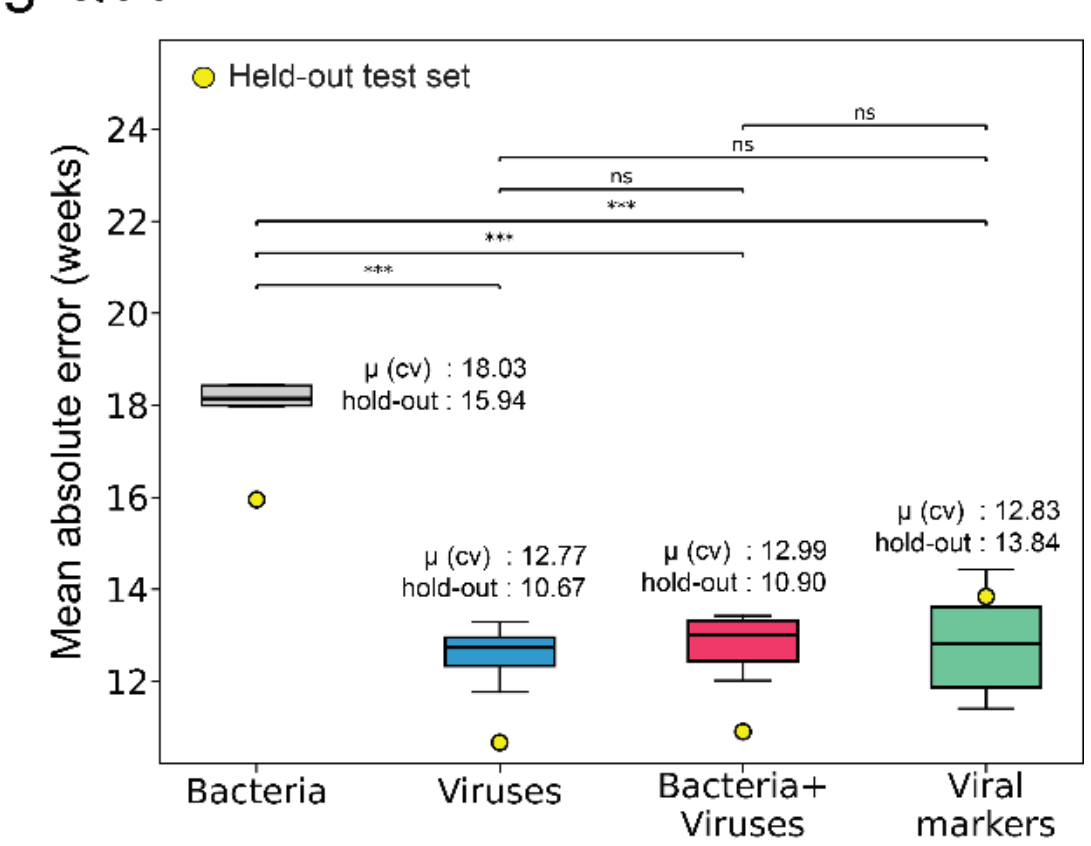

We also revised the manuscript as follows.

Line 407-408, 409, 412: *"The viral model outperformed the previously reported bacterial aging clock, achieving a lower mean absolute error (MAE) of 10.67 weeks compared to 15.94*

weeks on held-out test data (Fig. 6b; Supplementary Table 11). Integrating bacterial and viral features yielded negligible improvement (MAE = 10.90 weeks), indicating that the dominant aging signal is captured by the viruses. Moreover, a model based on 776 diet-independent viral aging markers (Methods; Supplementary Table 12) retained high predictive accuracy (MAE = 13.84 weeks), demonstrating that specific viral lineages serve as robust, diet-independent biomarkers of biological aging and can function as a non-invasive aging clock."

Line 830-834: ***"For downstream comparative analyses to mouse bacteriome, mouse bacteriome kraken2 database was constructed by 26,640 high-quality mouse pangenomes from MGBC112, upon which the 2,997 mouse bacteriome abundance was estimated using Kraken v2.3.1 and Bracken v3.0.1, followed by 10% coverage filtering and transforming to multiplicative CLR values identical to virome profiling."***

Line 846: ***"To prevent data leakage, samples from the same mouse (unique mouse ID) and the same cage (unique cage ID) were assigned exclusively to a single split (training, validation, or test)…"***

Fig.6 caption, Line 1285-1286: ***"b. Mice age predictions by 163 bacterial genera from MGBC pangenome catalog (Bacteria)…"***

We also revised **Supplementary table S11.**

External cohort validation was considered but not pursued, because publicly available mouse gut metagenomic datasets that meet the requirements for a meaningful test of an age prediction model are not currently available. In most candidate datasets, age information is either missing or restricted to a narrow range of young animals, and the underlying study designs are oriented toward disease or treatment rather than aging.

Q4) The biological interpretation of the virome-based aging clock requires further discussion. A substantial proportion of the viral signal used for age prediction may originate from temperate phages integrated into bacterial genomes. Because prophages are often highly strain-specific, the improved predictive performance over bacterial composition may partly reflect increased strain-level resolution rather than a truly independent virome-specific effect. To provide a fairer comparison, the authors might consider combining bacterial and phage reference databases and quantifying both using the same framework, for example Kraken-based abundance estimation (like Phanta, PMID: 37231259), followed by parallel modeling. In addition, a recent study has reported increased prophage abundance in the aging human gut across multiple bacterial taxa (PMID: 41275070), not only facultative anaerobes and pathobionts, which may be related to changes in bacterial diversity and ecology (PMID: 26982729). It is therefore possible that age-associated enrichment of temperate phages contributes substantially to the predictive power of the viral aging clock, and this possibility should be discussed more explicitly.

Response. We thank the reviewer for raising this important point. As described in our response to Q3, we re-profiled the bacterial abundance using a Kraken2 database built from the MGBC pan-genome, which incorporates bacterial strain-level resolution in the manner the reviewer suggests should be considered, while we maintained the virus profile as it is for fair comparison.

While the unified-database approach proposed by the reviewer (Phanta; PMID: 37231259) partially addresses the concern about bacterial strain-level resolution being reflected through prophage sequences, it is difficult to verify directly whether reads sequestered at the cross-domain root rank by Kraken2's LCA algorithm originate from prophage regions shared between the two reference sets, and whether Bracken redistribution subsequently reassigns these reads back to viral species in a way that reintroduces bacterial strain-level information into the viral profile. Because individual read provenance cannot be tracked through this pipeline, we evaluated **three complementary profiling schemes** whose differences directly bracket this uncertainty.

In the Independent scheme, reads were classified against the bacterial and viral Kraken2 databases separately. **In the Combined scheme**, reads were classified against a unified bacterial and viral database in the Phanta framework. **In the Sequential scheme**, reads were first classified against the bacterial database, and only unclassified reads were then classified against the viral database; this design assigns every cross-domain read (including all prophage-derived reads, which by definition match both reference sets) to the bacterial profile, so that the viral profile retains only signal that no bacterial reference can explain.

Under the Sequential scheme, the viral profile alone achieved age prediction performance comparable to that of the bacterial profile that captures all cross-domain prophage-derived signal by construction (**Fig. Q4a**). The bacterial profile augmented with this prophage-depleted viral profile yielded a further significant improvement in predictive accuracy over the bacterial profile alone. Taking together, these results indicate that the viral catalog carries aging-relevant predictive information that is not reducible to bacterial pan-genome content, and that viral profiling alone captures both the cross-domain prophage-derived aging signal and a viral-specific aging signal that is invisible to bacterial profiling.

Fig.Q4a

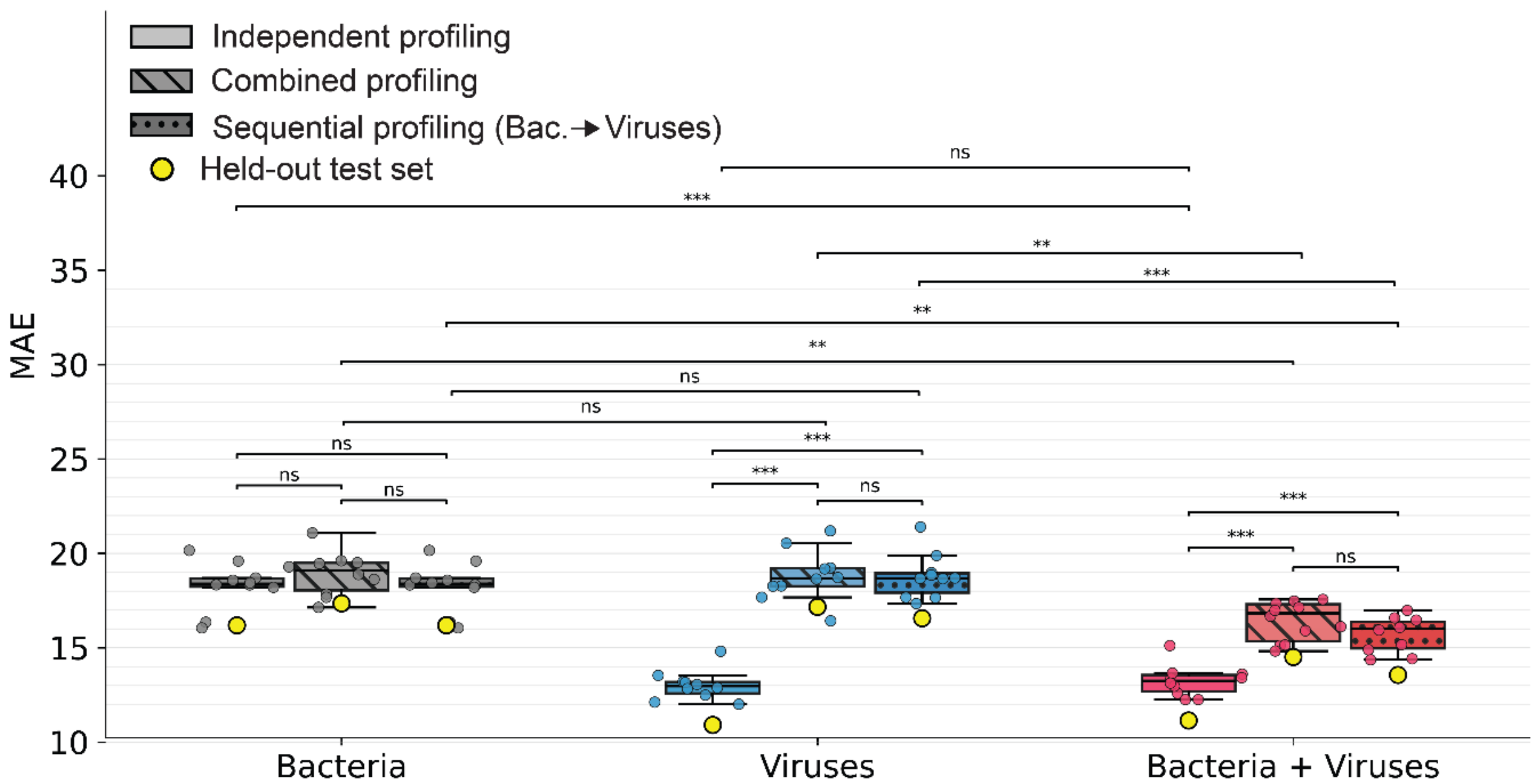

We agree with the reviewer's point that age-associated prophage expansion may occur across multiple bacterial taxa rather than being restricted to facultative anaerobes and pathobionts. To examine this in our data, we mapped the predicted bacterial hosts (GTDB R220 taxonomy, supported by CRISPR and/or integration evidence) of the 776 viral aging markers and compared them to the 4,800 non-marker genera with host assignments. Family-level enrichment analysis (Fisher's exact test, Benjamini-Hochberg FDR < 0.05) showed that the marker hosts include the facultative anaerobic and pathobiont lineages that were previously highlighted as expanding with age, namely Lactobacillaceae ($\log_2$OR = 2.92, P_adj = 4.4×10^{-15}) and Desulfovibrionaceae ($\log_2$OR = 2.80, P_adj = 6.9×10^{-4}), as well as several strict-anaerobic commensal and probiotic lineages that fall outside this category, including Bifidobacteriaceae ($\log_2$OR = 4.02, P_adj = 2.7×10^{-4}), Muribaculaceae ($\log_2$OR = 1.66, P_adj = 9.1×10^{-16}), Bacteroidaceae ($\log_2$OR = 1.29, P_adj = 3.5×10^{-3}), and Butyricicoccaceae ($\log_2$OR = 1.47, P_adj = 0.037) (Fig. Q4b). Given this taxonomically broad host distribution, we have accordingly revised our interpretation of the viral aging signal to reflect prophage-mediated dynamics across a wide range of commensal microbiota, rather than the original framing centered on facultative anaerobes and pathobionts alone.

Line 428-434: ***"Given that diverse bacterial lineages undergo compositional shifts with age due to the loss of colonization resistance and broader ecological remodeling of the gut microbiota[66,69], their resident prophages may concomitantly expand. This pattern is consistent with "Piggyback-the-Winner" dynamics, whereby high host density favors temperate phage propagation through lysogen expansion or density-dependent induction[61]. Accordingly, the***

viral aging clock likely captures amplified prophage signals from multiple age-shifting bacterial lineages, potentially serving as a sentinel of declining mucosal homeostasis."

Fig.Q4b

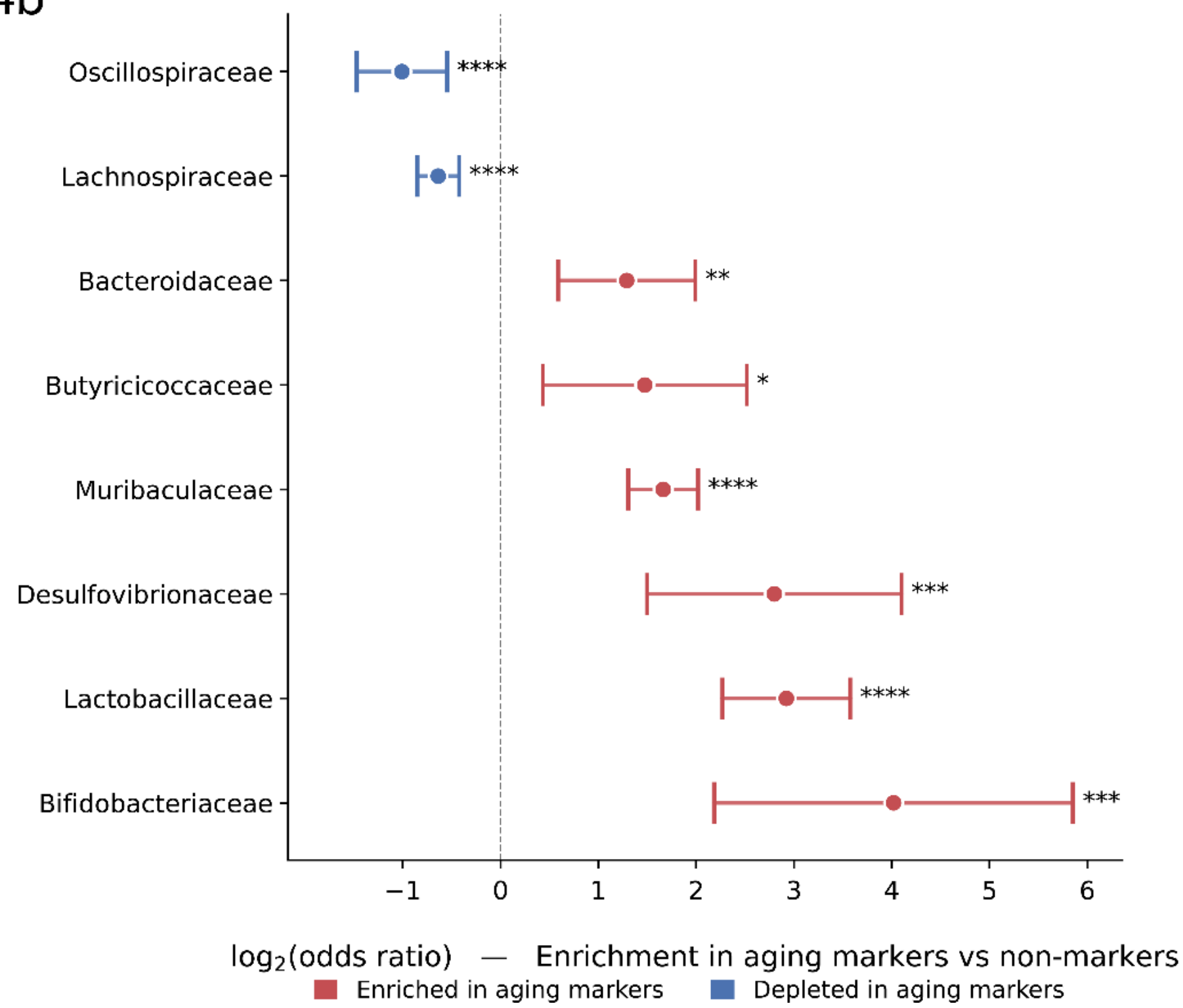

Q5) The manuscript would also benefit from improved balance between the Results and Discussion. The Discussion section currently does not cite any references, which limits the depth and context of the interpretation. In contrast, some parts of the Results section contain extended speculative discussion. For example, Lines 360–398 correspond to a single figure but include several paragraphs of interpretive discussion that would fit better in the Discussion section. Rebalancing these sections would improve readability and strengthen the overall presentation.

Response. We thank the reviewer for this helpful suggestion. We agree that the Results and Discussion sections require better balance. Accordingly, we revised the Results section to focus more clearly on the observed functional enrichment patterns, while toning down speculative or interpretive statements as follows.

Line 363-383: ***"Functional gene repertoires differ across mouse gut Crassvirales lineages***

Functional profiling revealed marked lineage-specific differences in accessory gene content (Fig. 5d). Steigviridae showed enrichment of lytic- and host-impact-associated annotations, including endolysin, toxin, and transcriptional regulator genes, whereas integrase-related modules were rarely detected. This pattern contrasted with Intestiviridae, which displayed

enrichment of integrase, excisionase, and repressor modules, together with anti-restriction, replication-support, and structural genes, consistent with a stronger representation of temperate-associated functions.

The Zeta subtype showed a similar enrichment of lysogeny-associated and regulatory modules, including CI-like repressors, integrase, transcriptional regulators, and an RNAP sigma factor. In addition, Zeta genomes were enriched for replication and nucleic-acid processing genes, including DNA primase, RNA ligase, endonuclease/exonuclease, homing endonuclease, and reverse transcriptase annotations, suggesting greater representation of genome-remodeling functions in this subtype. By contrast, the Epsilon subtype showed a host-specialist-associated profile, with enrichment of host-recognition modules, particularly tail fiber genes, together with amidase, transcriptional repressor, and nuclease annotations, but fewer genome-plasticity-associated functions.

Together, these results show that mouse gut Crassvirales lineages differ substantially in functional gene repertoires related to lysis, lysogeny, host recognition, replication, and genome remodeling. These differences provide genomic evidence consistent with lineage-specific variation in host breadth and predicted lifestyle across mouse gut crAss-like phages.”

We moved the broader biological interpretation of these findings to the Discussion section and added relevant references to provide appropriate context.

Line 475-505: ***“Despite this functional dark matter, the subset of annotated MRGV proteins revealed lineage-specific functional signatures within mouse gut Crassvirales, providing a genomic framework for interpreting variation in host breadth and predicted lifestyle among its lineages. Steigviridae was enriched for lysis- and host-impact-associated functions, including endolysin, toxin, and transcriptional regulator annotations, while showing little evidence of integrase-related modules. This pattern is consistent with a predominantly lytic genomic profile and agrees with previous observations that Steigviridae are frequently recovered under plaque-based experimental conditions and often encode elevated tRNA repertoires, which may support productive infection and partial translational autonomy during replication[70-72].***

In contrast, Intestiviridae and the Zeta subtype showed enrichment of integrase, excisionase, CI-like repressor, and transcriptional regulatory modules, suggesting a stronger association with temperate infection strategies. Such modules are central to lysogeny establishment, maintenance, and induction in diverse phage systems[73-76]. The concurrent enrichment of anti-restriction, replication-support, and structural genes in Intestiviridae may help explain its broad host range by supporting persistence across diverse host backgrounds while retaining the capacity for productive replication. Similarly, the enrichment of nucleic-acid processing genes, homing endonucleases, and reverse transcriptase in the Zeta subtype suggests increased genome-remodeling potential, which may contribute to lineage diversification and host-range flexibility[77,78].

The Epsilon subtype showed a contrasting pattern, with enrichment of host-recognition genes, particularly tail fiber annotations, together with fewer genome-plasticity-associated functions. Because tail fiber proteins are major determinants of host adsorption and specificity in tailed phages[79], this pattern is compatible with a more host-specialized ecological profile. The presence of amidase, transcriptional repressor, and nuclease annotations further suggests that Epsilon phages may retain efficient infection and replication functions within a narrower host niche[80]. Together, these findings support a model in which mouse gut Crassvirales lineages differ along a continuum from lytic-associated specialists to temperate broad-host-range lineages and host-specialized subtypes. Nevertheless, these lifestyle assignments are based on genome-derived functional signatures and should be validated in future studies using isolate-based infection assays, prophage induction experiments, or in situ virus–host linkage approaches."

We believe these revisions improve readability and more clearly separate the presentation of results from their interpretation.

Minor comments

Q1) The parameters used for MMseqs2 should be described more clearly in the Methods. MMseqs2 is used multiple times throughout the manuscript, but the relevant identity and coverage cutoffs are not always clearly stated. These parameters should be explicitly reported for each analysis.

Response. We agree with the reviewer that explicit reporting of MMseqs2 parameters is essential for reproducibility. The Methods section has been revised to clearly state the identity and coverage cutoffs applied in each instance where MMseqs2 was used as follows.

Line 684–686: ***"…with Pharokka's default search parameters: -e 1E-05 -s 8.5 for PHROGs, and –min-seq-id 0.8 -c 0.4 -s 8.5 for CARD and VFDB (--cov-mode 0 applied by MMseqs2 default)."***

Line 695–696: ***"…(--min-seq-id 0.9/0.7/0.5/0.3 with MMseqs2 default -c 0.8 –cov-mode 0 applied)."***

Line 701–702: ***"…(--min-seq-id 0.9/0.7/0.5/0.3 with MMseqs2 default -c 0.8 –cov-mode 0 applied),…"***

Line 709: ***"…(--min-seq-id 0.3 with MMseqs2 default -c 0.8 –cov-mode 0 applied)."***

Q2) The abbreviation "vOTU" is used throughout the main text before being formally defined in the Methods section. For clarity and readability, the authors should define "viral operational taxonomic unit (vOTU)" at its first appearance in the main text.

Response. We thank the reviewer for this helpful suggestion. The full term "viral operational taxonomic unit (vOTU)" has been added at its first appearance in the main text.

Line 110: ***"Species-level viral operational taxonomic units (vOTUs)…"***

Q3) At Line 1252, Part 7 (Kraken2 DB) of Extended Data Fig. 1 is not mentioned in the figure caption and should be added for completeness.

Response. We thank the reviewer for catching this omission. A description of Part 7 (Kraken2 DB) has been added to the caption of Extended Data Fig. 1

Line 1309-1311: ***"(Part.7) Construction of MRGV kraken2 database. MRGV Repr. DB was built with only 28,824 MRGV representative vOTU genomes, and MRGV Concat. DB was built with all 109,778 MRGV genomes"***

Reviewer #2

The manuscript by Kim et al., entitled "Incorporating viral genome binning in a mouse gut virome catalog enables accurate age prediction", presented the Mouse Reference Gut Virome (MRGV), a comprehensive viral catalog that substantially expanded the currently known diversity of mouse gut viruses. The authors applied viral genome binning and structure-based annotation, which respectively improved estimated genome completeness and functional coverage. Furthermore, they reported that gut virome features predicted host ageing more accurately than bacterial features, with most predictive markers originating from binned viral genomes. These aspects constituted the principle novelty of this study. The comprehensive mouse gut virom catalog is a valuable contribution to the field. However, I have several comments regarding the current version of the manuscript.

Major comments:

Q1) According to the developers, CheckV is not optimally suited for multi-contig viral genomes (PMID: 33349699). CheckV can estimate completeness but not contamination for viral MAGs. Given this limitation, how were viral bins assessed to meet the reported criteria of "Contam. = 0, no warnings" and other CheckV-based thresholds (Extended Data Fig. 1; Part 1; 1.7, Viral bin filtering)?

Response. We thank the reviewer for raising this important point, which we acknowledge was not described in sufficient detail in our original manuscript. As correctly noted, CheckV is not optimally designed for multi-contig viral genomes. We addressed this limitation as follows.

Concatenation strategy and source-code modification. Two approaches are currently in common use for applying CheckV to multi-contig vMAGs: concatenation with a fixed-length "N" spacer (PMID: 35544285), or direct concatenation without "N" sequences (PMID: 35181661). Because the former can introduce distortions during Prodigal gene calling, we adopted the latter and concatenated contigs using a 50-bp "N" spacer for all MRGV bin-level vMAGs. To prevent gene-calling artifacts arising from the inserted "N" stretches, we additionally modified the CheckV source code so that Prodigal is invoked with the masking option (-m) during gene prediction.

Contamination assessment. Although CheckV can compute a contamination value for multi-contig concatenated sequences https://bitbucket.org/berkeleylab/checkv/issues/71/how-to-run-checkv-on-prospective-viral), it cannot precisely delineate the host-flanking regions in this setting; we therefore considered bin-level CheckV contamination to be unsuitable as a primary metric. Accordingly, contamination was assessed primarily at the contig level: all host-flanking regions were removed from every contig prior to binning, and re-running CheckV on these processed contigs returns contamination = 0. When such contigs were subsequently concatenated

in bin-level vMAGs and re-evaluated, CheckV returned contamination = 0 in nearly all cases. For the small number of vMAGs in which contamination > 0 was still reported at the bin level, we considered that reliable quality assessment was not feasible and flagged these as QC-failed. Consequently, all retained MRGV genomes report contamination = 0, reflecting the fact that the analysis was performed on contigs whose host-flanking regions had already been removed at the contig level.

Completeness measurement. An important consideration is that "N"×50 concatenation adds a total of ("N"×50) × (number of contigs − 1) bp to the query. Because CheckV uses query length as a key determinant of completeness, we observed that the inserted spacers led to inflated completeness values. To correct this, we modified the CheckV source code so that the added spacer length is excluded when completeness is computed.

k-mer frequency. CheckV internally employs kcounter (https://github.com/apcamargo/kcounter) for k-mer-based estimation. Because "N" is not a valid nucleotide, these positions are excluded from k-mer counting and therefore introduce no bias.

Warning handling. With respect to CheckV warning messages, *"low-confidence DTR"* and *"low-confidence ITR"* indicate that the terminal repeat sequences required for a complete-genome call could not be predicted with high confidence; in such cases CheckV does not assign a complete-genome status but instead classifies the vMAG as High-quality, so these warnings do not compromise the quality estimate itself, and the corresponding vMAGs were retained. All other warnings, most notably *"query sequence is 1.5× longer than reference"* and *"no viral genes"*, were considered to indicate impaired genome quality, and every vMAG carrying such a warning was discarded.

We have also added the relevant methodological details to the Methods section as follows**.**

Line 588-591: ***"Because CheckV is not optimal to estimate contamination on multi-contigs fasta, host flanking regions in each 1,190,038 contigs were manually removed based on CheckV host coordinates, and checked contamination returns 0 after rerunning CheckV on the decontaminated 1,190,038 contigs before viral binning."***

Line 600-606: ***"Multiple contigs within each viral bin were concatenated with a 50-bp spacer of "N" characters between adjacent contigs prior to CheckV quality assessment. To prevent the spacer regions from biasing the quality metrics, we introduced minor modifications to the CheckV source code: (i) gene prediction was performed with Prodigal using the -m option, which prevents gene calls spanning runs of "N" characters; and (ii) genome length was recomputed by subtracting the total spacer length [(50 bp) × (number of contigs − 1)] from the concatenated sequence length."***

Q2) The representative contigs of high-confidence mouse gut vOTUs (hcMGV, n = 977; misrepresented in Line 52 of this manuscript) were all at least 90% complete, as viral candidates with flanking bacterial regions, low completeness (< 90%) or CheckV warnings were removed during catalogue construction to ensure high reliability (PMID: 40542420). The same study also reported a set of medium-quality mouse gut vOTUs (mqMGV, 50% = < completeness < 90%, n = 1645), defined independently from hcMGV.

Given that the MRGV in the present manuscript (n = 28,824) included viral genomes with at least 50% completeness, it would be more appropriate to include hcMGV and mqMGV together, rather than hcMGV alone, in the comparisons shown in Fig. 2a, 2h, and 2i, to provide a more comprehensive and balanced reference to existing mouse gut virome resources.

In addition, clarification is needed for Fig. 2a. The authors reported that 23.05% of MRGV (n = 6,644) overlapped with 76.66% of hcMGV (n = 749). These percentages appeared asymmetric. Please reconcile and provide a rationale for the apparent discrepancy in counts.

Response. We thank the reviewer for this important clarification. We agree that, for assessing the expansion of mouse gut virome diversity, it is more appropriate to compare MRGV against both high-confidence mouse gut vOTUs (hcMGV) and medium-quality mouse gut vOTUs (mqMGV). We therefore repeated the vOTU overlap analysis using the combined hcMGV and mqMGV set. As a result, 32.44% of MRGV vOTUs (9,351/28,824) overlapped with 66.52% of hc+mqMGV vOTUs (1,744/2,622), while 67.56% of MRGV vOTUs (19,473/28,824) remained unique **(Fig.Q2a,b, Table Q2a)**.

Fig.Q2a

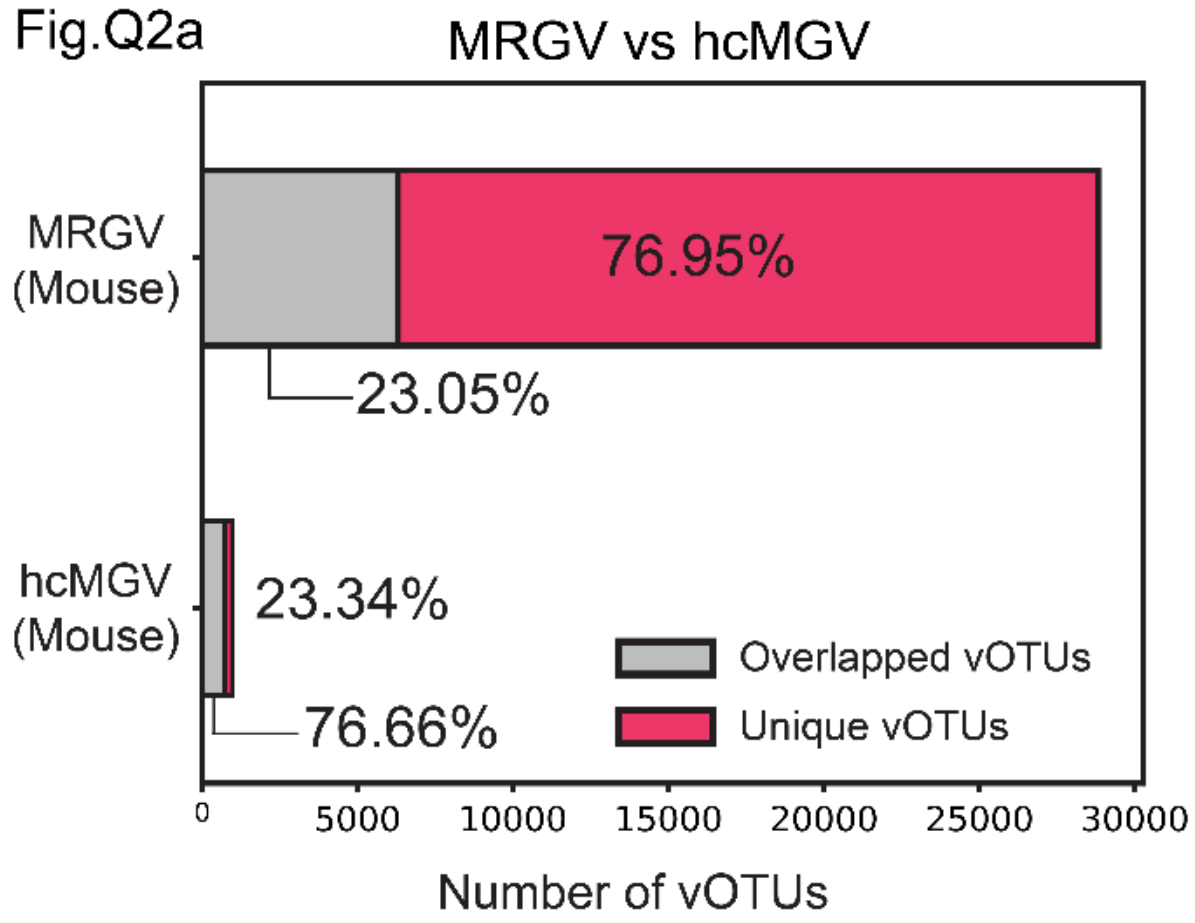

Fig.Q2b

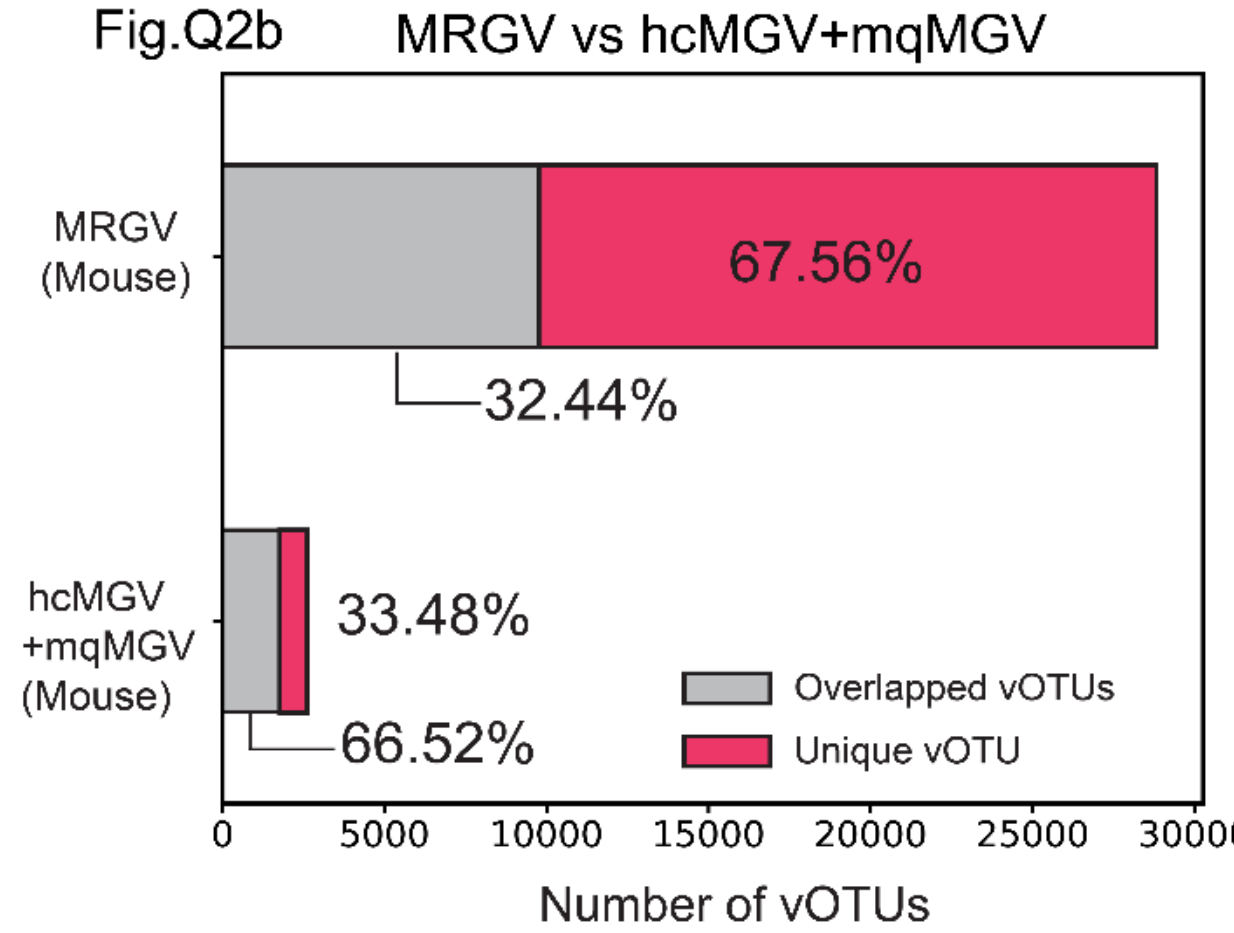

[Table.Q2a]

Database	Overlap (vOTUs)	Unique (vOTUs)	Total vOTUs
MRGV	1,744	878	2,622
hcMGV	9,351	19,473	28,824

We have revised **Main Fig. 2a with Fig.Q2b** and the corresponding text accordingly and corrected the misreported description of hcMGV+mqMGV.

Line 29: *"…expanding known mouse gut viral diversity by ~67.6%."*

Line 61: *"MRGV expands known mouse gut viral diversity by ~67.6% at the species level compared to hcMGV."*

Line 132-133: *"Approximately 67.6% (19,473 of 28,824) species-level vOTUs did not cluster with species from the former mouse virome catalog"*

Line 143-144: *"…reported 977 high-confidence (hcMGV) and 1645 medium-quality (mqMGV) species-level vOTUs derived from 407 mouse WMS datasets."*

Line 145: "… between hqMGV+mqMGV and MRGV at the…"

Line 146-148: *"Although 67.56% (19,473 of 28,824) of MRGV species-level vOTUs were not represented in hcMGV+mqMGV, 66.52% (1,744 of 2,622) of hcMGV+mqMGV species-level vOTUs were recovered by MRGV…"*

For the read-classification analyses in **Main Fig. 2h,i**, however, we retained hcMGV as the reference database for comparison. These analyses were intended to evaluate database-level classification performance rather than catalogue-level diversity expansion. The original study constructed and benchmarked hcMGV as a conservative high-confidence genome database, whereas mqMGV was reported as an independent medium-quality vOTU set. Combining hcMGV and mqMGV for read classification would require constructing a new non-redundant hc+mqMGV database, including re-clustering the two independently defined vOTU sets and selecting new representative genomes, to avoid redundancy-driven biases in read assignment. Because such a newly constructed database would no longer correspond to the original conservative hcMGV resource the author intended, we considered hcMGV as the appropriate reference for database-performance comparisons in **Main Fig. 2h,i.**

The apparent asymmetry in overlap percentages is due to the different catalogue sizes and our conservative comparison strategy. To avoid overestimating MRGV-specific vOTUs, we assessed overlap at the vOTU level using both representative and member genomes of MRGV. Specifically, an MRGV vOTU was considered non-unique if the representative genome of an hc+mqMGV vOTU matched either the representative genome of that MRGV vOTU or at least one of its member genomes at ≥95% ANI and ≥85% coverage. Thus, even a single qualifying match to any genome within an MRGV vOTU was sufficient to classify that MRGV vOTU as overlapping with hc+mqMGV. This conservative criterion reduces the risk of overestimating MRGV-specific vOTUs. Accordingly, we revised and specified in method section as follows.

Line 730-735: *"Comparison of gut virome diversity*

MRGV vOTUs (n=28,824) were compared with 977 hcMGV and 1,645 mqMGV vOTUs[10] and 168,536 UHGV vOTUs[9] using skANI (v0.1.4). To avoid overestimating the number of MRGV-unique vOTUs, an MRGV vOTU was considered overlapping with an external vOTU when at least one of all genomes including representative and its member genomes satisfied an ANI ≥ 95% and aligned fraction ≥ 85% cutoff against that external vOTU."

Q3) The authors stated that 31.83%, 12.76%, 11.22%, and 19.36% of linked viruses respectively spanned multiple genera, families, orders, and even phyla. This conclusion requires better support. Are these connections supported by 1 spacer match? Given that the accuracy of CRISPR array prediction, especially in metagenomic data, is less than perfect, what are the chances that these matches are spurious? It is recommended to improve the reliability of these connections by increasing the threshold to multiple viruses (three or more) exhibiting connections with the two host species on both ends.

Response. We thank the reviewer for this important suggestion. We agree that broad host-breadth assignments based on CRISPR spacer matches require additional support, even though we used a more stringent CRISPR spacer-matching cutoff than that used in previous virome cataloguing studies. In the original manuscript, host breadth was primarily presented from a virus-centric perspective, and therefore did not explicitly test whether broad-rank connections were supported by multiple viruses linking the same host species pairs.

To address this concern, we performed an additional bacteria-centric host species-pair analysis following the reviewer's recommendation. Specifically, **for CRISPR-supported links, two bacterial host species were considered to form a valid cross-rank connection only when they were connected by at least three distinct viral genomes.** This threshold was applied to CRISPR-derived broad-host connections at the cross-genus, cross-family, cross-order, cross-class, and cross-phylum levels. **Integration signals were treated separately because they represent a distinct and stronger form of virus–host association than CRISPR spacer matches; therefore, integration-supported host links were not subjected to the CRISPR-specific three-virus threshold**.

During this re-analysis, we also identified and corrected a minor annotation-handling issue in the original procedure. In GTDB R220, some bacterial lineages lack classification at intermediate ranks, such as family or genus. The original code grouped such missing-annotation entries together at the affected rank, which could incorrectly merge species from different upper taxonomic ranks. We corrected this by assigning cross-rank relationships only when both host species had non-missing annotations at the rank being compared. This correction caused only minor changes in the reported fractions and did not alter the overall conclusion **(Fig. Q3a).**

Fig.Q3a

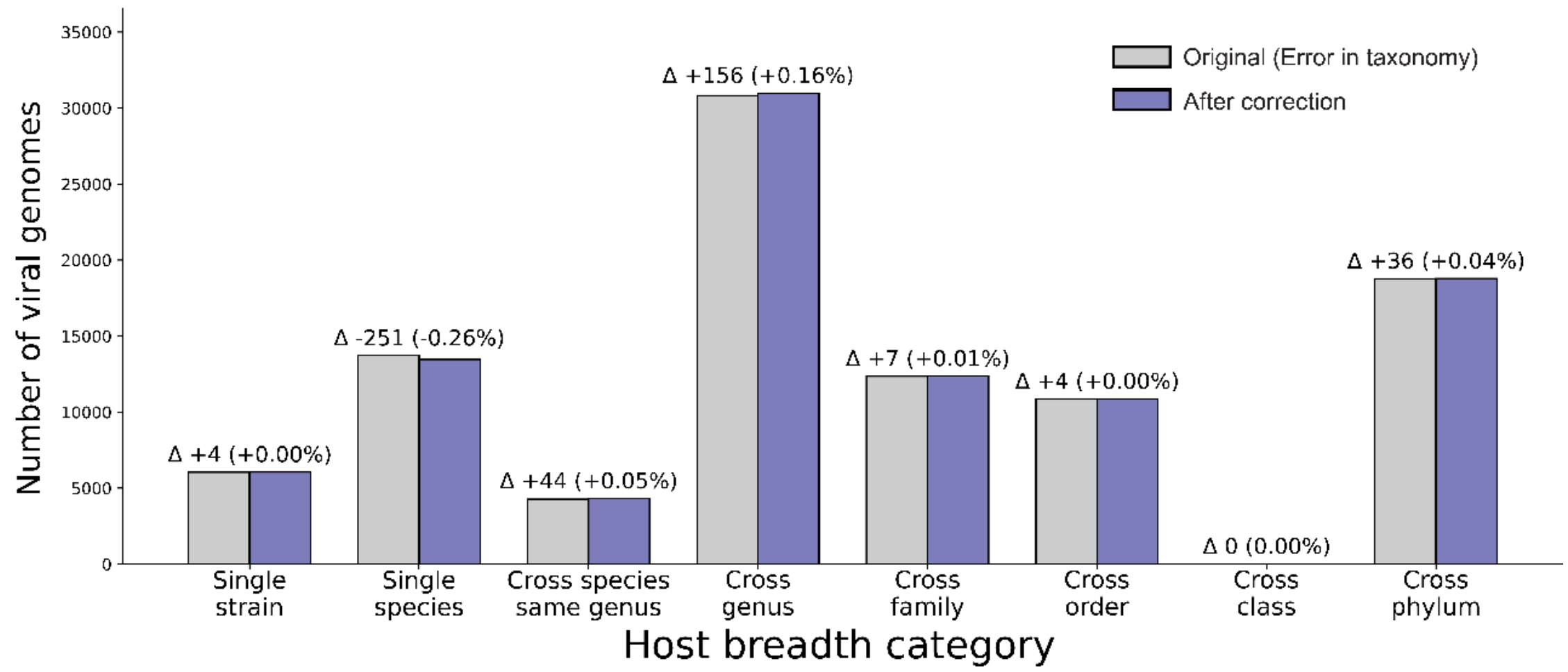

After applying the reviewer-suggested threshold to CRISPR-supported broad-rank connections, a total of 6,406 viral connections no longer met the required support. Specifically, 3,101 viral genomes at the cross-genus level, 1,335 at the cross-family level, 981 at the cross-order level, and 988 at the cross-phylum level did not pass the three-virus species-pair threshold **(Fig. Q3b). The reduction was driven mainly by viral genomes supported by both CRISPR and integration evidence, because CRISPR-derived endpoints in this group were filtered by the reviewer-guided criterion while integration-supported endpoints were retained separately (Fig. Q3c,d).**

Fig.Q3b

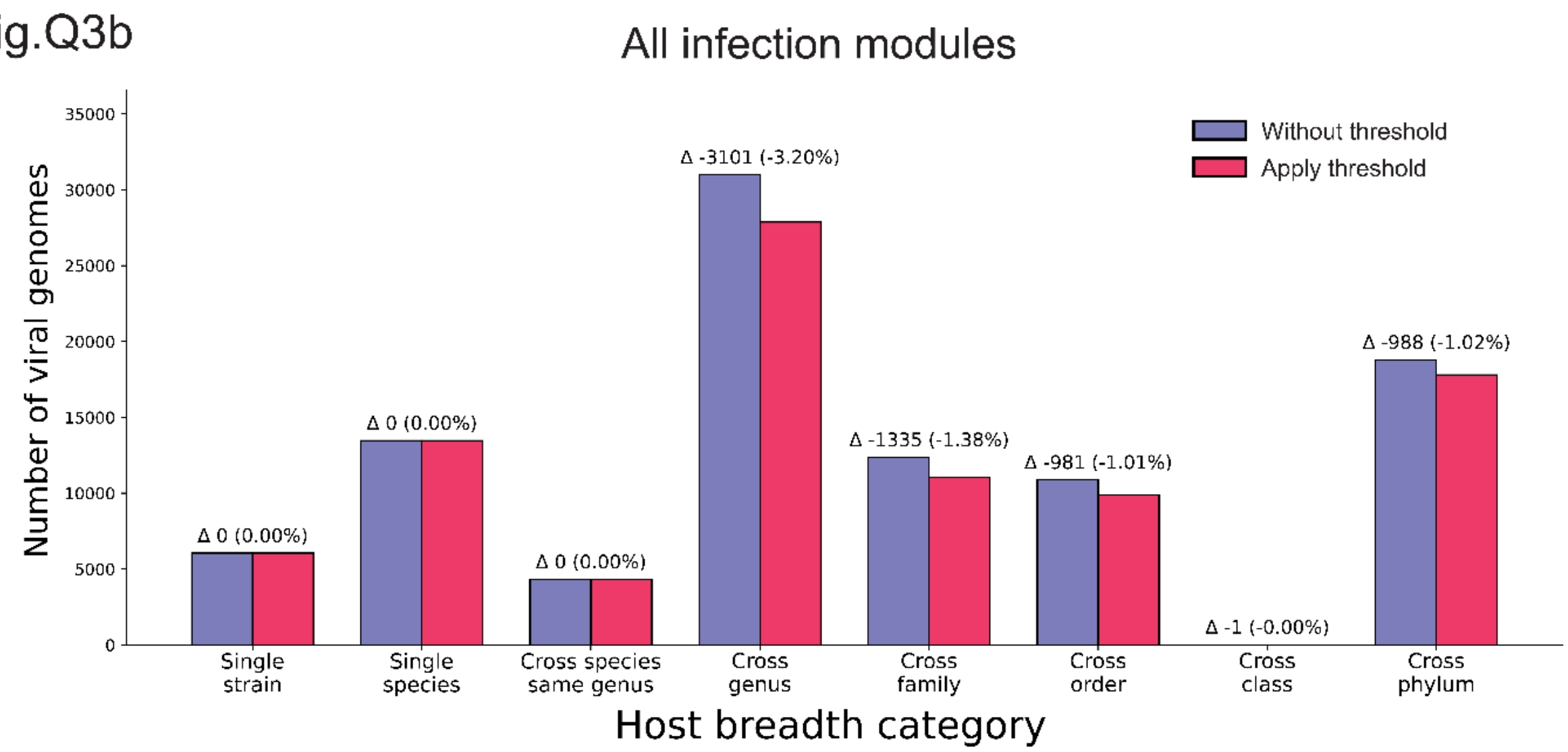

FIg.Q3c

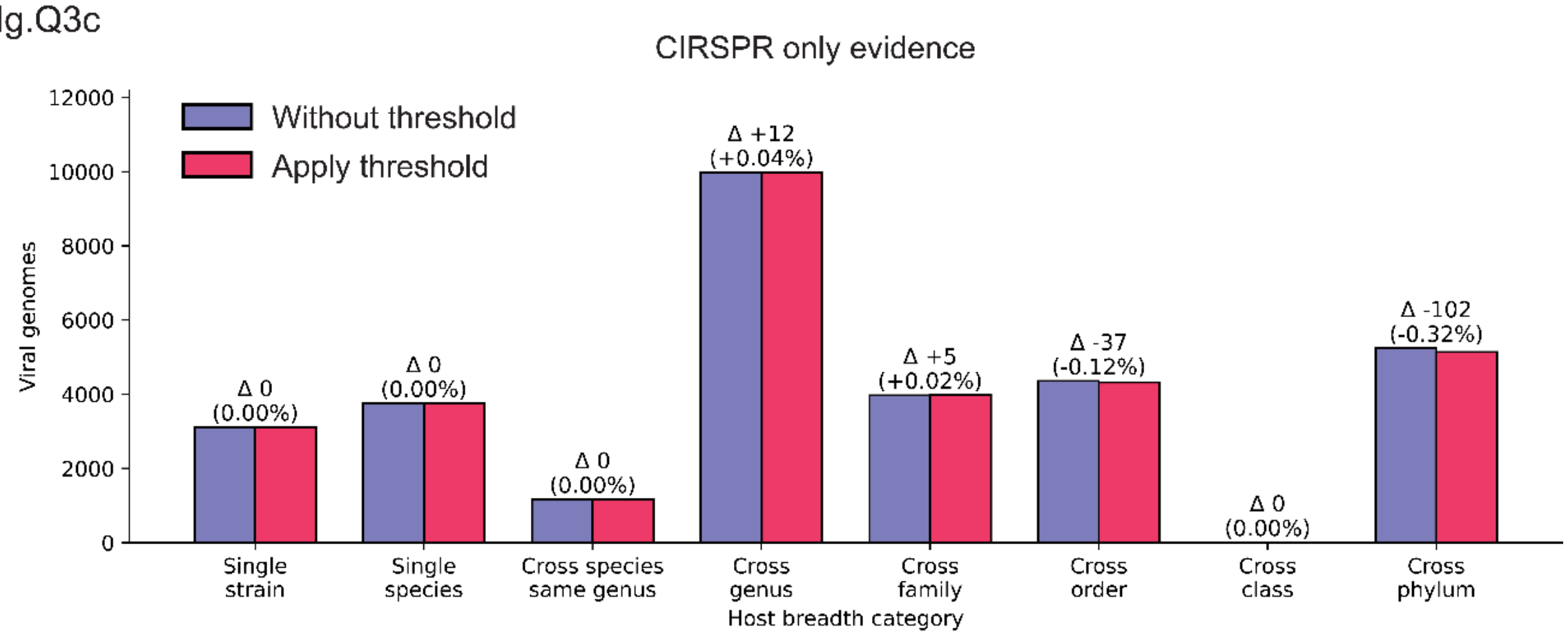

FIg.Q3d

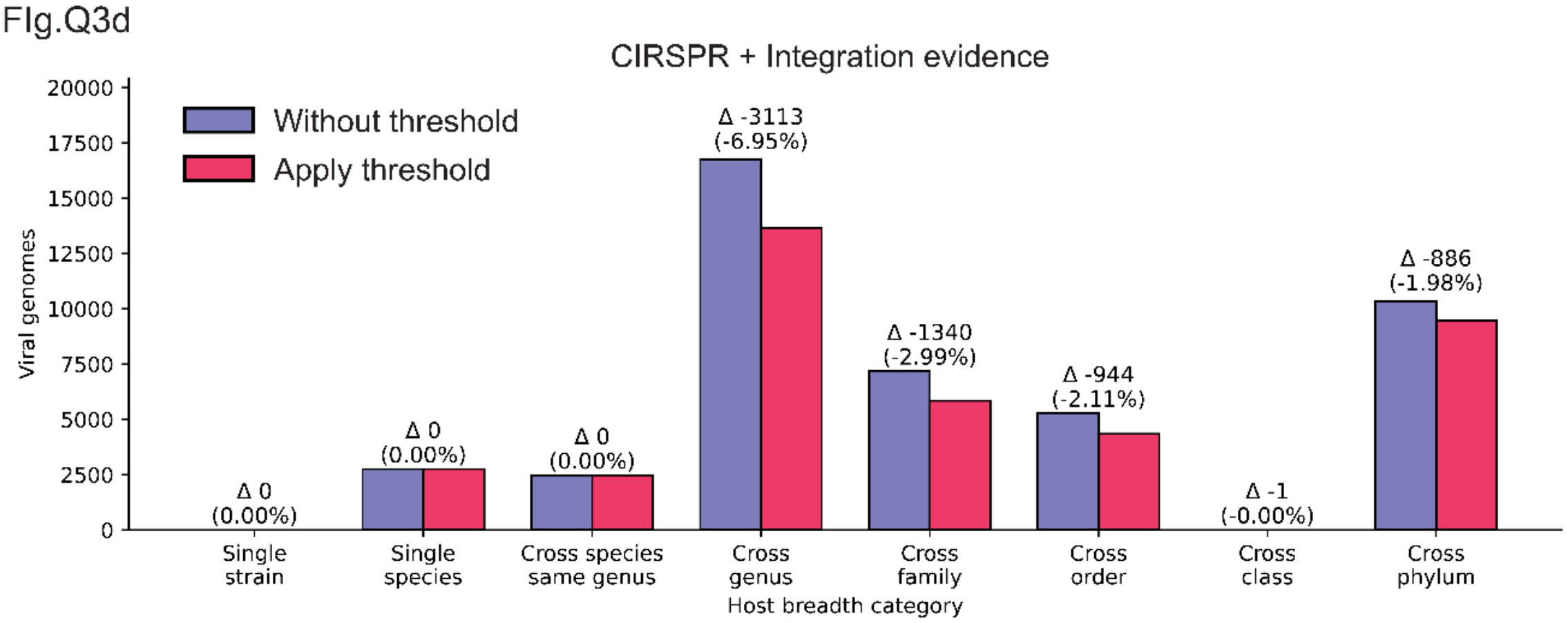

Overall, the broad host-breadth pattern remained consistent after applying this more conservative criterion. To reflect the revised analysis, we updated the host-breadth results using the threshold-filtered CRISPR-supported connections and replaced **Main Fig. 4e** accordingly. We also revised the manuscript to describe the species-pair threshold analysis, the treatment of CRISPR and integration evidence**.**

Line 290-291: ***"Across the 96,850 host-linked viral genomes, we filtered 90,444 host-linkage genomes based on evidence support filtering (Methods)."***

Line 292-294: ***"Only 6,052 genomes (6.69%) were linked to a single strain and 13,456 (14.89%) to a single species, whereas many spanned multiple genera (30.83%), families (12.19%), orders (10.94%), or even phyla (19.68%)."***

Line 769-777: ***"Linkage evidence-based host breadth analysis***

In total, 96,850 viral genomes spanning 22,758 vOTUs that were assigned to a bacterial host at least to the phylum level had at least one host connection via CRISPR spacer matching and/or prophage integration. To reliably classify the host breadth of each viral genome, a cross-rank host connection (Multiple genera, families, orders, classes, or phylum) was

accepted only when the corresponding pair of host species at the boundary of that rank was independently linked by at least three distinct viral genomes via CRISPR spacer matching. Because integration evidence provides a direct sequence-level link between viral and host genomes, a single supporting viral genome was considered sufficient for an integration-based connection."

Minor comments:

Q1) Line 43, this ratio has been updated to phage: bacteria ~1:1 or 1:10 by many studies.very old paper is being cited here. Please update**.**

Response. We thank the reviewer for pointing out that the originally cited reference (Abeles and Pride, 2014) reflects outdated estimates derived largely from environmental rather than gut-specific measurements. We have updated the corresponding sentence based on the recent quantitative study by Lopez et al. (2025, *Current Biology*; PMID: 40300605), which integrated quantitative imaging, metagenomic sequencing, and mathematical modeling of phage-host dynamics to estimate gut phage abundance. Their analysis indicates that, in the human gut, the ratio of phage genomes to bacterial genomes (including integrated prophages and phage-plasmids) is approximately 4:1. We have therefore revised the statement to reflect this updated estimate and replaced the outdated reference with Lopez et al. (2025) accordingly as follows.

Line 41–43: ***"This bacterial-centric view overlooks bacteriophages, whose abundance in the human gut varies substantially from approximately 1:100 to 4:1 relative to bacteria, reflecting the balance between lytic and lysogenic lifestyles[3]."***

Q2) How to distinguish the lytic vMAG and lysogenic vMAGs?

Response. Lifecycle assignment was performed using a two-step hierarchical approach. In the first step, we used skANI, which supports multi-contig genome alignment, to detect direct integration signals between viral MAGs and host bacterial genomes. A viral MAG was classified as a prophage (lysogenic) when it showed ≥99% ANI and ≥99% alignment coverage with a host bacterial genome under skANI, indicating integration within the host genome. In the second step, for viral MAGs that did not show alignment-based integration signals in the first step, lifecycle was inferred based on three additional criteria: (1) prediction as a prophage by at least one of four viral detection tools (CheckV, VIBRANT, Phigaro, or geNomad); (2) presence of an integrase gene; and (3) presence of integration-associated accessory genes. A viral MAG was classified as lysogenic when it satisfied at least two of these three criteria, and as lytic otherwise. This procedure is described in the Methods section *"Inference of lysogenic and lytic viral lifestyle"* of the original manuscript (**Line 713–728).**

We also note that the original manuscript erroneously listed DeepVirFinder as one of the prophage prediction tools in this step. This was a typographical error, as CheckV was the tool actually used, and the manuscript has been corrected accordingly (**Line 721).**

Q3) Is there a specific rationale for selecting DeepVirFinder, VIBRANT, and Phigaro as the primary viral identification tools, with geNomad and VirRep applied as additional filters? According to the developers, VirRep is dedicated to human gut metagenomic samples, the effectiveness of VirRep to other biomes could be further elevated by fine-tuning it on that biome-specific data (PMID: 38965579). Was any such fine-tuning performed before applying VirRep to mouse gut metagenomes in this study?

Response. Our tool selection reflects both the chronological availability of viral identification methods at the time of analysis and the distinct functional roles of these tools within our workflow.

When this project was initiated, neither geNomad nor VirRep had been released. We therefore selected the primary viral identification tools based on benchmarking results available at the time (PMID: 37085924). To capture novel viral sequences with limited reference homology, we selected DeepVirFinder, which uses a deep learning approach. To capture previously characterized viral lineages with high sensitivity, we selected VIBRANT, which integrates homology searches with machine learning. To enhance prophage detection sensitivity, we additionally included Phigaro. These three tools were chosen to provide complementary detection across distinct categories of viral sequences.

Following the subsequent release of geNomad and VirRep, we incorporated both tools as validation filters for viral sequences already identified by the primary detection workflow, rather than as additional *de novo* detection tools. This design choice was motivated by the previously reported risk that combining multiple viral identification tools for detection, without rigorous validation, can lead to overestimation of viral diversity (PMID: 39019907). By restricting geNomad and VirRep to a validation role, we aimed to constrain false positives while preserving the sensitivity contributions of the original tool combination.

Regarding fine-tuning of VirRep, we did not perform biome-specific fine-tuning on mouse gut metagenomic data, for two reasons. First, VirRep was applied in this study for validation of pre-identified viral sequences rather than for *de novo* detection, so the cost-benefit balance differs from a detection-focused application in which biome-specific sensitivity is the primary goal. Second, the reference dataset of mouse gut-specific vMAGs is substantially smaller and less comprehensively curated than the human gut virome dataset on which VirRep was originally trained. In our assessment, fine-tuning on such a sparse and incompletely characterized reference

set would risk introducing bias into the model's confidence estimates rather than improving them, thereby compromising the reliability of validation.

Q4) More details should be provided when describing the construction and analysis workflow.

We thank the reviewer for this constructive comment. Although we sought to build upon established methodologies and to refine those that we found insufficient, we agree that several aspects of the workflow were not communicated with sufficient clarity in the original submission. We have accordingly revised the Methods section to fill in missing details and to refine minor descriptions throughout, with particular attention to viral binning quality assessment as suggested by the reviewer.

Q5) L821: web portal (https://www.decodebiome.org/MRGV/) is not available.

Response. We apologize for the inconvenience. The portal was temporarily inaccessible during a scheduled server-infrastructure upgrade at our host institution and has since been fully restored at https://www.decodebiome.org/MRGV/. To ensure long-term, persistent accessibility, we have deposited all data and code in Zenodo **(https://zenodo.org/records/20115989, DOI: 10.5281/zenodo.20115989)** and GitHub **(https://github.com/netbiolab/MRGV.),** which now serve as the primary distribution channels and provide version-controlled, DOI-anchored access independent of any institutional server. The decodebiome.org portal continues to be maintained as a complementary interactive interface. The Data and Code Availability section has been revised accordingly.

Line 870-873: ***"All MRGV-associated resources, including viral genomes, protein sequences, metadata, and the Kraken2 database, are publicly accessible at https://zenodo.org/records/20115989 (DOI: 10.5281/zenodo.20115989). The analysis code, source data, and trained prediction models are publicly available at https://github.com/netbiolab/MRGV."***

Q6) Extended Data Fig. 1: Part 1, 1.5, "Lytic" instead of "Lityc"; Part 4, 4.2 "Meet" instead of "Meed"; Part 7, legend is missing.

Response. We thank the reviewer for the careful reading. The typographical errors have been corrected ("Lityc" to "Lytic" in Part 1.5, and "Meed" to "Meet" in Part 4.2), and the missing legend for Part 7 has been added.
